# Definition of constitutive and stage-enriched promoters in the rodent malaria parasite, *Plasmodium yoelii*

**DOI:** 10.1186/s12936-020-03498-w

**Published:** 2020-11-23

**Authors:** Laura M. Bowman, Logan E. Finger, Kevin J. Hart, Scott E. Lindner

**Affiliations:** Department of Biochemistry and Molecular Biology, The Huck Center for Malaria Research, Pennsylvania State University, University Park, PA USA

**Keywords:** Stage-enriched promoter, Gene editing, *Plasmodium yoelii*, Malaria parasite

## Abstract

**Background:**

Well-defined promoters are essential elements for genetic studies in all organisms, and enable controlled expression of endogenous genes, transgene expression, and gene editing. Despite this, there is a paucity of defined promoters for the rodent-infectious malaria parasites. This is especially true for *Plasmodium yoelii*, which is often used to study the mosquito and liver stages of malarial infection, as well as host immune responses to infection.

**Methods:**

Here six promoters were selected from across the parasite’s life cycle (*clag-a*, *dynein heavy chain delta*, *lap4*, *trap*, *uis4*, *lisp2*) that have been invoked in the literature as controlling their genes in a stage-specific manner. A minimal promoter length for the constitutive *pybip* promoter that confers strong expression levels was also determined, which is useful for expression of reporters and gene editing enzymes.

**Results:**

Instead, it was observed that these promoters confer stage-enriched gene control, as some parasites also effectively use these promoters in other stages. Thus, when used alone, these promoters could complicate the interpretation of results obtained from promoter swaps, stage-targeted recombination, or gene editing experiments.

**Conclusions:**

Together these data indicate that achieving stage-specific effects, such as gene editing, is likely best done using a two-component system with independent promoter activities overlapping only in the intended life cycle stage.

## Background

Malaria remains one of the greatest global health issues today, with hundreds of millions of new infections and nearly half a million fatalities occurring annually [1]. Control of this disease has been challenging due to the nature of the eukaryotic *Plasmodium* parasites that cause it, the *Anopheles* mosquitoes that transmit it, and the dynamics of human populations and governments. Despite this, great strides have been made in discovering and developing new drugs that can selectively target the parasite, in developing insecticide-treated bed nets that can prevent mosquitoes from biting people, and in advancing subunit and live-attenuated vaccine candidates that can reduce the overall number of malarial infections. However, more interventions are needed to reduce the incidence of malaria as the parasite is able to rapidly evolve drug resistance, and the worldwide diversity of parasite field isolates limits vaccine efficacy.

In order to find key weaknesses of the parasite that can be exploited by new therapeutics, it is important to interrogate the entire *Plasmodium* life cycle in both mammals and mosquitoes. While these studies can be done with human-infectious *Plasmodium* parasites (e.g., *Plasmodium falciparum*, *Plasmodium vivax*), they are cumbersome to do, require ex vivo culture conditions, access to patient samples (for *P. vivax*), and/or use costly humanized mice or non-human primates [2, 3]. Instead, many laboratories turn to the use of rodent-infectious *Plasmodium* species, such as *Plasmodium yoelii*, *Plasmodium berghei*, and *Plasmodium chabaudi*. Studies with these parasites are routine and also allow for observations of host/pathogen interactions. However, key discoveries made in these model species must then be validated in human-infectious species. Among these model species, *P. yoelii* in some ways aligns more closely to human-infectious *P. falciparum* than do others. For instance, the duration of the mosquito stage of development is of equal length (~ 14 days), and the infectivity of sporozoites for host cells is less promiscuous than seen in *P. berghei* (which can productively infect and develop in non-hepatocytes) [4, 5].

Reverse genetic methods to study rodent-infectious *Plasmodium* species are well established and robust but suffer from several technical limitations. First, only one positive drug selectable marker (DHFR) is commonly used for the selection of transgenic parasites [6, 7]. While the use of fluorescent proteins for selection has also been described, this method can be difficult to implement and eliminates their use for future studies [8]. This limits gene editing of a parasite line to one or few events, unless marker recycling processes such as GIMO or recombination-based approaches are used [9–11]. However, these approaches require significant effort and additional mice to remove the marker and recreate a drug sensitive line. The adoption of CRISPR-based gene editing sidesteps this problem, unless the editing plasmid(s) inadvertently integrate into the genome and thus leaves the drug selectable marker behind [12–14]. In these cases, negative selectable markers can be used to select against parasites with integrated plasmids, but this also requires significant time and mice to achieve the desired parasite line [15].

Second, the transfection efficiency of all studied *Plasmodium* species is notoriously low, often resulting in only 0.002–0.1% of parasites taking up and establishing a plasmid in order to become drug resistant [6, 16]. Finally, in some *Plasmodium* species such as *P. yoelii*, very few gene control elements have been robustly defined and minimized. This has led to the common practice of producing unnecessarily large plasmids with multiple instances of identical promoters and UTRs, which often leads to recombination-based loss of plasmid elements during propagation in *Escherichia coli* [13]. While all of these problems warrant concerted efforts to study and solve them, here perhaps the most straightforward of these limitations was addressed: the definition of gene control elements.

Here, the stage specificity of commonly used promoters from across the life cycle of rodent-infectious malaria parasites *P. berghei* and *P. yoelii* was interrogated. Promoters from constitutive and stage-specific genes are commonly used for transgene expression to achieve gene editing, recombination, protein/RNA labeling, and restricted expression conditions [17–19]. It was found that these promoters are not specific per se, but rather are enriched in the previously defined “specific” stage with additional expression occurring at appreciable levels in other stages of the life cycle. In some cases, this non-stage specific expression may simply be a nuisance and increase noise when these promoters are used as markers. However, when restriction of expression to a specific stage is required, such as in some genome editing strategies, expression in unintended stages can confound the interpretation of those results and limit the utility of those parasites.

## Materials and methods

### Experimental animals

Six- to eight-week old Swiss Webster female mice, acquired from Envigo, were used for each experiment performed. *Anopheles stephensi* mosquitoes, originally acquired from the Center for Infectious Disease Research (Seattle, WA, USA), were reared at 24 °C and 70% humidity and were used to experimentally produce *P. yoelii* 17XNL strain parasites.

### Generation of plasmids

Primers (provided in Additional File 1) were designed to amplify portions of the *pybip* promoter consisting of either ~ 300 or ~ 500 bp upstream of the translational start site. Alternatively, promoter regions of *pyclag-a*, *pydynein heavy chain delta* (*pydd*), *pylap4* (also called *pyccp2*), *pytrap*, *pyuis4*, or *pylisp2* were also designed by selecting 1500–1800 bp upstream of their translational start sites. PCR amplicons were produced by Phusion polymerase (NEB), and were gel extracted (QIAquick Gel Extraction Kit, Qiagen, Cat# 28706), precipitated with ethanol, and ligated into pCR-Blunt (Life Technologies). Promoter sequences (provided in Additional File 2) were verified via Sanger Sequencing (Penn State Sequencing Core), digested with restriction enzymes, and ligated into pSL0489 to replace the *P. berghei eef1a* (*pbeef1a*) promoter. This plasmid backbone contains a Green Fluorescence Protein mutant 2 (GFPmut2) cassette for visualization and a human dihydrofolate reductase (HsDHFR) cassette for drug selection. Plasmids were linearized by cutting between the two arms of the *p230p* targeting sequences. The linearized plasmid was then precipitated with ethanol, resuspended in water, and transfected into the parasites as described below.

### Transfection of *Plasmodium yoelii* 17XNL strain parasites

Transfections were carried out as previously described with a few modifications [6]. Briefly, *P. yoelii* (17XNL strain) infected mice were exsanguinated through cardiac puncture. The blood was placed in 5 ml complete RPMI [cRPMI: 20% FBS in RMPI 1640 with gentamicin (50 mg/ml, Invitrogen Cat #15750-060)]. The blood was centrifuged at 200 *xg* for 8 min to pellet cells and allow the removal of serum. Blood cells were then resuspended in a closed T75 flask with 30 ml per mouse cRPMI and was mixed with a gas mixture consisting of 5% CO_2_, 10% O_2_, and 85% N_2_. The parasites were cultured for 12 h at 37 °C on a gradual incline and slightly shaken by an orbital shaker at 50–60 rpm. Following the 12-h incubation, thin-blood smears were stained with Giemsa to ensure that the parasites had been synchronized to mature schizonts.

After the verification by Giemsa staining, 10 ml of 17% w/v Accudenz dissolved in 5 mM Tris pH 7.5@RT, 3 mM KCl and 0.3 mM EDTA in 1 × PBS without calcium and magnesium was layered beneath the 30 ml layer of the parasite culture in a 50 ml conical tube. The mixture was then spun at 200×*g* for 20 min with no brake. Schizonts that migrated to the interface of the two layers were collected, pelleted by centrifugation at 200×*g* for 10 min, and resuspended in 50–200 µl cRPMI. Ten micrograms of 1 mg/ml linearized plasmid in ddH_2_O was added to 100 µl cytomix (120 mM KCl, 0.15 mM CaCl_2_, 2 mM EDTA, 5 mM MgCl_2_, 8.66 mM K_2_HPO_4_ pH 7.6, 1.34 mM KH_2_PO_4_ pH 7.6 and 25 mM HEPES pH 7.6 @RT). Ten microliters of purified schizonts were added and mixed with a wide-bore pipette, and then transferred to a cuvette. Electroporation was carried out using an Amaxa Nucleofector 2b with program T-016. Fifty microliters of cRPMI was added to the electroporated parasites, which were then immediately injected intravenously into mice. The recipient mice were placed on pyrimethamine (0.007% w/v, final concentration, Fisher Scientific, Cat# ICN19418025), administered in the drinking water, one day post transfection and remained on drug for three days. Medicated water was then replaced with standard water, and parasites were allowed to reach a parasitemia of 1%. Infected blood (100 µl) was used to infect a naïve mouse by intraperitoneal injection, and the drug cycling was repeated. Upon reaching 1% parasitemia, the mouse was exsanguinated, a portion of the infected blood was stored in cryovials in liquid nitrogen, with the remainder was used to extract genomic DNA for genotyping PCR. Mixed populations containing transgenic parasites were used for all analyses.

### Transmission of *Plasmodium yoelii* parasites to *Anopheles stephensi* mosquitoes

Infected blood containing the transgenic parasites-of-interest was injected intraperitoneally into naïve mice to initiate an infection. The number of exflagellation centers within confluent fields of erythrocytes (centers of movement, “COMs”) were assessed daily to determine peak transmissibility from mouse to mosquito. When COMs were determined to be at their peak (generally > 1 COM/40 × microscopic field), the mice were fed to mosquitoes. Mice received an intraperitoneal (IP) injection of an anesthetic (100 mg/kg ketamine, 10 mg/kg xylazine mixture in 1 × PBS without calcium and magnesium), with two mice infected with the same transgenic line being fed to a cage of starved mosquitoes for 15 min with rotation occurring every five minutes to allow for even feeding. The midguts of the mosquitoes were dissected seven days post-feed to determine oocyst numbers and the proportion of infected mosquitoes. Three days later (day 10), mosquito midguts were dissected, ground and the oocyst sporozoites within were counted using a Hausser Bright Line-Phase hemocytometer (Fisher Scientific, Cat# 02-671-6) and stained for IFA as described below. Fourteen days post-feed, the salivary glands of the mosquitoes were dissected then ground to release the sporozoites. The sporozoites were then counted using a hemocytometer, and stained for IFA as described below.

### Flow cytometry

Wild-type or transgenic parasites containing portions of the *pybip* or *pbeef1a* promoter driving GFPmut2 expression were synchronized to schizonts (to reduce variability in GFP abundance associated with different asexual blood stages), and were Accudenz purified as described above. An LSR Fortessa (BD) in tube mode was used to measure the samples’ fluorescence and the data was analysed via FlowJo.

### Indirect immunofluorescence assay (IFA) of blood stage parasites

Indirect immunofluorescence assays (IFAs) of asexual and sexual blood stage parasites were conducted essentially as described previously [20, 21]. All centrifugation steps occurred at room temperature at 200×*g* for a duration of 30 s.

The blood of infected mice was collected and pelleted. The cells were washed twice with 1 × PBS followed by fixation (4% v/v paraformaldehyde, 0.00625% v/v glutaraldehyde in 1 × PBS) for 3 h at room temperature. The cells were then permeabilized, at room temperature, in permeabilization solution (0.1% Triton X-100 v/v in PBS) for 10 min. Following permeabilization, the cells were mixed with blocking solution (3% w/v bovine serum albumin (BSA) in 1 × PBS) and allowed to sit for 1 h at room temperature. The primary antibodies (rabbit anti-PyACP (1:1000, Pocono Rabbit Farm and Laboratory, Custom polyclonal antibody), rabbit anti-PyCITH (1:1000, Pocono Rabbit Farm and Laboratory, Custom polyclonal antibody), mouse anti-alpha tubulin (1:1000, Sigma Aldrich Catalog #T5168), mouse anti-GFP (1:1000, DSHB, Clone 4C9), rabbit anti-GFP (1:1000, Invitrogen, A11122) were diluted in blocking solution, added to the cells and allowed to bind for an hour as previously described [22, 23]. Cells were then washed twice with 1 × PBS and the secondary antibodies (Alexa Fluor-conjugated (AF488, AF594) specific to rabbit or mouse IgG (Invitrogen, Cat# A11001, A11005, A11008, A11012) were diluted 1:500 in blocking solution, and added to the cells in the dark for one hour. The cells were washed once with 1 × PBS and then stained with 1 µg/ml DAPI (4′,6-diamidino-2-phenylindole) in 1 × PBS for 5 min at room temperature. The cells were washed twice with 1 × PBS and mixed 1:1 with VectaShield Hard Set (Vector Laboratories), applied to a glass slide with a coverglass slip, and sealed with nail polish.

### Indirect immunofluorescence assay (IFA) of sporozoites

Indirect immunofluorescence assays (IFAs) of oocyst sporozoites and salivary gland sporozoites were conducted essentially as described previously [24]. Sporozoites collected from the midguts or salivary glands of mosquitoes were fixed in ~ 100 µl of 10% v/v formalin and allowed to incubate for 10–15 min at room temperature. They were then centrifuged 15 k×*g* in the microcentrifuge for 3 min and aspirated. The sporozoites were resuspended in an adequate amount of 1 × PBS and were quantified via a hemocytometer. Twenty-five thousand sporozoites or greater were loaded in each well on a 12-well, PTFE printed slide (Electron Microscopy Sciences) and the slides were allowed to air dry. Sporozoites were washed with 1 × PBS for 2 min, permeabilized with 0.1% v/v Triton X-100 in 1 × PBS for 10 min, washed with 1 × PBS, and then blocked for 1 h with 10% w/v BSA in 1 × PBS. The mixture was then exposed to mouse anti-PyCSP (Clone 2F6) and rabbit anti-GFP (Invitrogen, A11122) primary antibodies diluted 1:1000 in 10% w/v BSA in 1 × PBS for 30 min. Sporozoites were then washed three times with 10% w/v BSA in 1 × PBS, and then treated with Alexa Fluor-conjugated (AF488, AF594) secondary antibodies specific to rabbit or mouse IgG (Invitrogen, Cat# A11005, A11008) for 30 min. Parasites were then washed twice with 1 × PBS and incubated in the dark with 1 μg/ml of DAPI in 1 × PBS for 5–10 min. Finally, parasites were washed three more times in 1 × PBS, combined with an equal volume of VectaShield Hard Set (Vector Laboratories), and applied to glass slides as above.

### Indirect immunofluorescence assay (IFA) of liver-stage parasites

Indirect immunofluorescence assays (IFAs) of liver stage parasites were conducted essentially as described previously [25]. Livers from mice infected with *P. yoelii* were sliced with a microtome (DSK MicroSlicer Zero1). Two to three liver slices were washed with 1 × PBS and placed in a solution of 3% v/v hydrogen peroxide and 0.25% v/v Triton X-100 in 1 × PBS on an orbital shaker for 30 min. They were washed again for 10 min with 1 × PBS and blocked with 5% w/v dried milk in 1 × PBS for 1 h on an orbital shaker. The slices were washed for 10 min with 5% w/v dried milk in 1 × PBS and exposed to primary antibodies diluted 1:1000 in 5% w/v dried milk in 1 × PBS [mouse anti-PyCSP (Clone 2F6), a custom rabbit anti-PyACP antibody, mouse anti-GFP (DSHB, Clone 4C9), rabbit anti-GFP (Invitrogen, A11122)] for 1 h. The slices were then washed with 5% w/v dried milk for 10 min and exposed to secondary antibodies diluted 1:500 in 5% w/v dried milk in 1 × PBS [Alexa Fluor-conjugated (AF488, AF594) specific to rabbit or mouse IgG (Invitrogen, Cat# A11001, A11005, A11008, A11012)] for 2 h in the dark at room temperature with shaking. Liver slices were then treated with 1 µg/ml DAPI in 1 × PBS for 5–10 min in the dark at room temperature with shaking. Samples were then washed twice with 1 × PBS for 10 min, exposed to 0.06% w/v potassium permanganate for 20 s and washed a final time in 1 × PBS. The treated liver slices were then placed on a poly-lysine coated microscope slide with VectaShield (Vector Laboratories) and were sealed under a coverglass slip with nail polish.

### Fluorescence microscopy

A Zeiss Axioscope A1 with 8-bit AxioCam ICc1 camera, using a 63 × air or 100 × oil objective, was used to perform all live fluorescence and IFA imaging. Live fluorescence was performed to monitor GFP expression in transgenic blood-stage parasites containing the GFP-expression plasmid driven by the 300 and 500 bp *pybip* promoter, as well as transgenic day 7 oocysts. Fluorescence microscopy was used to monitor the other parasite life cycle stages after the appropriate IFA was performed. Zen imaging software (Zeiss) was used to analyse the images.

## Results

### Generation of reporter parasite lines

**Fig. 1:**
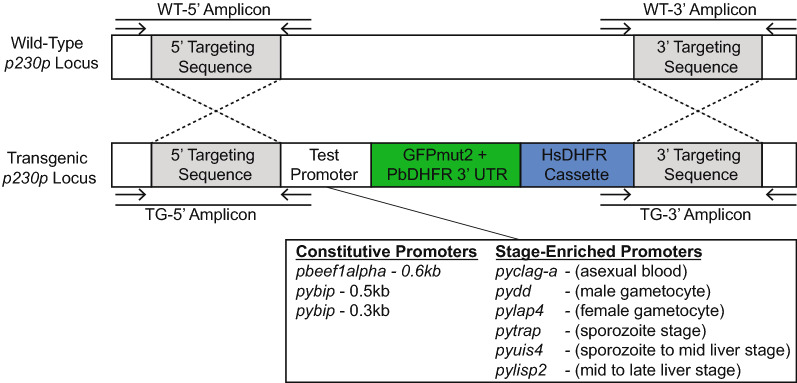
Integration of test promoters into the *p230p* safe harbor locus of *Plasmodium yoelii*. Plasmids bearing a single test promoter (see inset) driving GFPmut2 expression were linearized and integrated into the *pyp230p* locus by double homologous recombination. Transgenic parasites were selected by resistance to pyrimethamine via constitutive expression of human dihydrofolate reductase (HsDHFR). Populations containing transgenic parasites were used for all experiments. Promoter sequences were used from *pyclag-a* (PY17X_1402200), *pydd* (PY17X_0418900), *pylap4* (PY17X_1323300), *pytrap* (PY17X_1354800), *pyuis4* (PY17X_0502200), *pylisp2* (PY17X_1004400), *pybip* (PY17X_0822200), and *pbeef1a* (PBANKA_1133300)

Genetic studies often leverage well-defined promoters to control for expression of genes-of-interest and transgenes. In organisms with complex development and life cycles such as the malaria parasite (genus *Plasmodium*), there are cascades of gene expression that are crucial for stage progression [26, 27]. Therefore, defining constitutive promoters that are active throughout the life cycle, as well as stage-specific promoters, will yield useful tools for reverse genetic approaches. As there are few well-defined promoter elements for *P. yoelii*, a simple plasmid (pSL0489) was created for insertion of promoter candidates upstream of GFPmut2 that can be integrated into the *p230p* dispensable (safe harbor) locus (Fig. 1) [28]. This base plasmid uses the “gold standard” strong, constitutive *Plasmodium berghei* elongation factor 1 alpha (*pbeef1a*) promoter to drive GFP expression as is commonly used in studies with rodent-infectious *Plasmodium* parasites. GFP expression in this line is evident throughout their entire life cycle as per live fluorescence and indirect immunofluorescence assays [28].

From the literature, promoters that have been described to be strong and constitutive or stage specific as observed in other *Plasmodium* species were selected for study. To produce as diverse of a promoter collection as possible, individual promoters with expression profiles that span the complete life cycle of *Plasmodium* parasites were chosen. The *clag-a* promoter (PY17X_1402200) has been defined as restricting transcription to the asexual blood stage of the related rodent malaria parasite *P. berghei* [19]. The *dynein heavy chain delta* (“*dd*”, male, PY17X_0418900) and *lap40* (also called *ccp2*, female, PY17X_1323300) promoters have been commonly used in the “820” male/female fluorescent protein reporter line (820cl1m1cl1) [29, 30]. The *trap* promoter (PY17X_ 1354800) is known to be active throughout sporozoite development and is critical for salivary gland invasion, gliding motility, and infectivity [31], whereas the *uis4* promoter (PY17X_0502200) flips from being weakly active in oocyst sporozoites to becoming one of the strongest promoters in salivary gland sporozoites [32, 33]. Finally, the *lisp2* promoter (PY17X_1004400) becomes active in mid-liver stage and remains so throughout liver stage development [34]. Finally, the *bip* promoter (PY17X_0822200) was also selected, as it is known to be a strong and constitutive promoter in eukaryotes due to the integral role that the BiP protein plays in the translocation of nascent, unfolded proteins into the ER [35]. In previous work, a 1.5 kb portion of sequence upstream of the *bip* coding sequence was used to serve as a promoter for CRISPR-based gene editing [13]. Here, two truncated variants were created in an attempt to produce a strong, minimal constitutive promoter.

Promoters tested here typically included 1.6–1.8 kb of sequence upstream of (and including) the start codon, and were designed to place a unique restriction site 3′ of the start codon to avoid effects upon translation that may occur if placed upstream of it. Complete primer and promoter sequences (Additional Files 1 and 2) are described in the supporting materials. Sequence-verified promoters were used to replace the *pbeef1a* promoter of pSL0489, which also contains a separate HsDHFR expression cassette to provide antifolate resistance, and the resulting plasmids were linearized and transfected into *P. yoelii* 17XNL strain parasites. The resulting transfected parasites were selected with pyrimethamine and genotyped by PCR (Additional Files 3, 4, 5, 6, 7, 8, 9, 10), which indicated that transgenic parasites were produced as intended. Experiments were carried out with mixed populations of parasites containing the desired transgenic parasites to determine when the promoter was active and to qualitatively assess expression levels at these stages. Here indirect immunofluorescence assays (IFAs) were used for most stages due to its greater sensitivity, but use live fluorescence microscopy for some comparisons as well.

### Characterization of a strong minimal pybip promoter

Constitutive promoters are essential *cis* elements for most reverse genetic approaches. However, few are well defined for use in rodent-infectious *Plasmodium* species. Commonly used plasmids make use of the elongation factor 1 alpha promoter from *P. berghei* (“*pbeef1a*”), which is short, can be used in a bidirectional format, and allows for high levels of transcription throughout the life cycle. However, this promoter is often placed in multiple locations on a single plasmid (e.g., pDEF, B3d backbones), which can lead to recombination of the plasmid and loss of the sequences between them. Additionally, the RNA polymerase III U6 promoter is now frequently used for expression of single guide RNAs (sgRNAs) for CRISPR-based gene editing due to the definition of its 5′ and 3′ ends [14, 36]. Recently, it was also shown that very long (~ 1.5 kb) segments of DNA upstream of the coding sequences of *pygapdh*, *pydhfr*, and *pybip* are also transcriptionally active in asexual blood stage parasites [13].

**Fig. 2:**
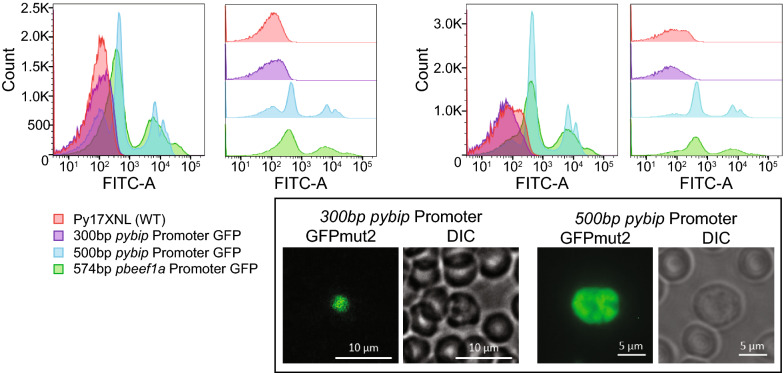
Definition of a strong *pybip* promoter. Transgenic parasites bearing a 300 bp or 500 bp portion of the region upstream of the *pybip* coding sequence were compared to the commonly used strong, constitutive *ef1alpha* promoter from *Plasmodium berghei*. Flow cytometric analyses of purified asexual blood stage schizonts from two independent experiments (left: overlay, right: separated) are shown. Live fluorescence micrographs of representative parasites for each parasite line are provided in the inset

In order to create a compact, constitutive promoter and to monitor its transcriptional strength in a population of parasites, one of two short (~ 300 bp, ~ 500 bp) versions of the *pybip* promoter were fused upstream of GFPmut2, as was previously done for the *pbeef1a* promoter [28]. Asexual blood stage parasites were synchronized to schizonts in an ex vivo culture to reduce differences in protein levels that could be attributable to the smaller ring and trophozoite stage parasites. As seen in biological duplicate by flow cytometry assays (Fig. 2, left and right), the control *pbeef1a* promoter and 500 bp *pybip* promoter constructs allowed transcription above background fluorescence of untransfected Py17XNL parasites, while the 300 bp *pybip* promoter construct did not. Transcription from the *pbeef1a* promoter produced a broader and stronger expression distribution, including two discernable populations of high/highest expression. Similarly, the 500 bp version of the *pybip* promoter allowed robust transcription with two discernable peaks of gene expression. While no appreciable expression was detected by flow cytometry for the 300 bp *pybip* promoter, a few cells with GFP above background levels were detected by live fluorescence microscopy (Fig. 2, inset). Together this indicates that *cis* elements critical for robust transcription reside in the − 500 to − 300 region of this promoter, and that a compact and strong *pybip* promoter can be defined.

### Characterization of blood stage promoters—clag-a*,* dd*,* lap40.

**Fig. 3:**
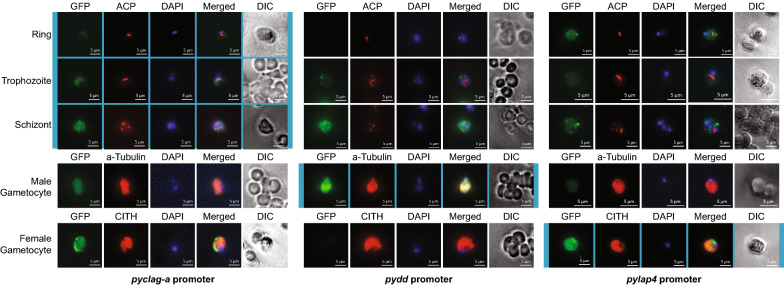
Indirect immunofluorescence assay of *P. yoelii* parasites using blood-stage promoters to express GFPmut2. Asexual and sexual blood stage parasites were stained with anti-GFP antibodies and stage-defining, counterstaining antibodies (ACP, alpha-tubulin, CITH). Previously defined stage-specific expression is indicated by light blue shading behind the respective micrographs. Scale bars are 5 µm

Stage-restricted promoters are often used to study genes in the blood stage of the *Plasmodium* life cycle, and often are intended to allow expression in only its asexual blood stages (ring, trophozoite, schizont), or in either of the sexual gametocyte stages. Promoters were selected that have been previously used in *P. berghei* for each of these stages, and have placed them upstream of a GFP coding sequence as described above for the *pybip* and *pbeef1a* promoters. GFP expression was assessed by indirect immunofluorescence assays (IFA) across the entire life cycle of *P. yoelii*, with representative micrographs from the blood stages (Fig. 3) and all stages (Additional Files 11, 12, 13) presented. Stages that were previously noted to have specific expression have background shading behind the micrographs.

In addition to their previously described expression times, it was also observed that some parasites had an expanded expression window. The *pyclag-a* promoter, which has been used in promoter swap experiments as an asexual blood stage specific promoter to prevent expression in gametocytes, does also allow expression in both male and female gametocytes (Fig. 3, left panels), as well as late liver stage parasites as seen in published RNA-seq data (Additional File 11). Inversely, the *dynein heavy chain delta* (*dd*) and *lap4* promoters have been used for male-specific or female-specific expression respectively, such as in the 820 line of *P. berghei* [29, 30]. In agreement with this, it was observed here that the strongest expression does occur in these stages, but that some low-level expression is also present in asexual blood stages (Fig. 3, middle and right panels). Thus, use of these promoters for reporter lines should be studied in those cells selected to have the highest expression in order to more strictly study male and female gametocytes.

### Characterization of mosquito and liver stage promoters*—*trap*,* uis4*,* lisp2

**Fig. 4:**
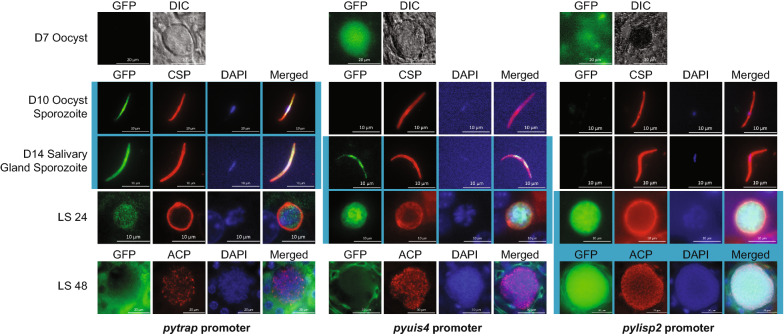
Indirect immunofluorescence assay of *P. yoelii* parasites using mosquito- or liver-stage promoters to express GFPmut2. Oocysts were visualized by live fluorescence and DIC microscopy. Oocyst sporozoite, salivary gland sporozoite, and 24-h and 48-h liver stage parasites were stained with anti-GFP antibodies and stage-defining, counterstaining antibodies (CSP, ACP). Previously defined stage-specific expression is indicated by light blue shading behind the respective micrographs. Scale bars are 10 or 20 µm

Similarly, promoters with activity that is restricted to specific portions of the mosquito or liver stage are useful tools for studying these more technically challenging stages. Three promoters were selected that have been defined as having mosquito stage (*pytrap*), late mosquito stage/early liver stage (*pyuis4*), or mid-to-late liver stage (*pylisp2*) expression for characterization as above. Representative IFA micrographs from mosquito and liver stage (Fig. 4) and complete IFA panels from across the life cycle (Additional Files 14, 15, 16) are provided. Those stages where expression has been previously noted are background shaded.

While these analyses found that the published expression profiles do represent the stages with the strongest activity of these promoters (Fig. 4), it was observed that some parasites used these promoters robustly in additional stages. The *trap* promoter has been thoroughly described as being active during sporozoite development and important to invasion of the salivary gland [31, 37]. This promoter is active in approximately half of female gametocytes and some male gametocytes as well, in contrast to what was observed in the Malaria Cell Atlas for the related parasite *P. berghei* (Additional File 14) [38].

The *upregulated in infectious sporozoites 4 (uis4)* promoter has been shown to have very low activity in oocyst sporozoites but produces one of the most abundantly transcribed mRNAs in salivary gland sporozoites [32, 33]. Expression of *uis4* continues through early and mid-liver stage, where the UIS4 protein serves an essential role [39]. Moreover, the *uis4* mRNA is known to be translationally repressed until transmission to the mammalian host. However, the *cis* element that provides for translational repression activity is known to not be provided by the promoter/5′UTR, and thus this promoter is useful for exceptionally high expression of transgenes in salivary gland sporozoites/early-mid liver stage [40]. In agreement with previous work, robust expression in salivary gland sporozoites through mid-liver stage was observed. Additionally, a few asexual blood stage schizonts, male and female gametocytes, and oocysts also had robust expression (Fig. 4; Additional File 15).

Finally, the *lisp2* promoter in other *Plasmodium* species is a well-defined, mid-to-late liver stage promoter that produces a protein that is essential for late liver stage parasites [34, 41–43]. Robust activity in mid-to-late liver stages was detected, but also some female gametocytes with moderate expression were also observed (Fig. 4; Additional File 16). Together, the general expression profiles observed for these promoters in other *Plasmodium* parasites apply to *P. yoelii*, but additional activity is present in other stages as well. Therefore, caution is warranted when using them for tightly regulated expression in a parasite population.

## Discussion

With the advent of CRISPR-based genome editing and other more traditional reverse genetic approaches for *Plasmodium* parasites, there is a fundamental need for reliable and defined gene expression to study parasite biology and to develop transgenic parasite lines. Moreover, with the desire to edit parasite genomes at precise moments in its life cycle, now robustly possible with readily re-programmable nucleases, having a suite of defined promoter elements is especially important. However, this need has been largely overlooked, with many studies using the same gene control elements for a variety of purposes. Worse yet, these elements (promoters, terminators) are often used multiple times in single plasmids, which leads to unexpected recombination of the DNA sequences in *E. coli* and in the parasite itself.

Here, constitutive and non-constitutive promoters of *P. yoelii* were characterized through IFA and live fluorescence microscopy, whereby representative images of the highest expression as seen across the entire life cycle are provided. In this work, specific promoter sequences from seven genes were defined, and in the case of *pybip*, expression of a minimal strong promoter variant in individual parasites of a population was quantified. These defined promoters will aid the design of plasmids for reverse genetic experiments with *P. yoelii*, and due to the high levels of sequence conservation across rodent-infectious *Plasmodium* parasites (Additional File 17), they likely can be directly applied to studies with *P. berghei* or *P. chabaudi* as well.

**Fig. 5:**
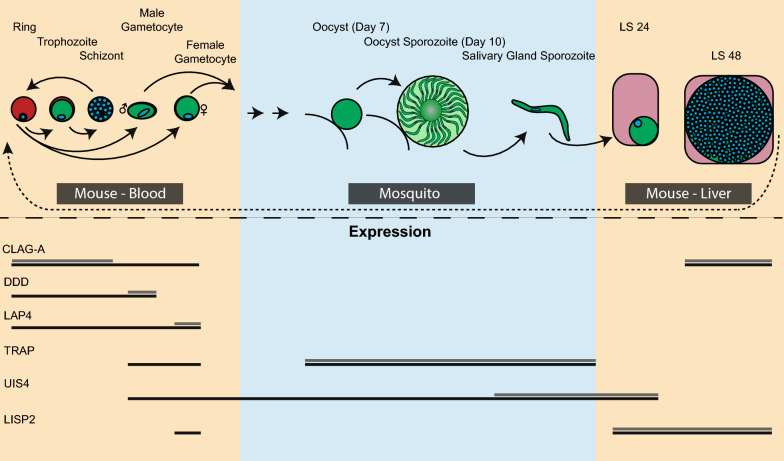
Summary of stage-enriched and overall promoter activities. Promoter activities where the highest expression in most parasites is observed is depicted with a light gray line, whereas additional expression observed in other parasite life cycle stages is denoted with a black line. Restricting stage-specific activities may best be achieved via two component systems where independent promoter activities only intersect at the desired life cycle stage

These characterized promoters primarily drive transcription in a stage-enriched manner and this is not tightly stage restricted in all parasites. The enriched and total expression profiles of these promoters is summarized in Fig. 5, their complete sequences are provided in Additional File 2, and a comparison of these sequences across *P. yoelii*, *P. berghei*, and *P. chabaudi* is provided in Additional File 17. These findings nicely match the predominant expression profiles that have been seen in previous bulk RNA-seq and single cell RNA-seq experiments (available in PlasmoDB.org), but find that some parasites also have abundant expression from these promoters in other stages [23, 28, 32, 38]. These spurious events could potentially confound interpretation of the data or when used for CRISPR approaches, could lead to gene editing events at unintended times in a subset of the parasites. That these promoters are not strictly stage restricted is perhaps not surprising based upon recent single cell RNA sequencing (scRNA-seq) data provided within the Malaria Cell Atlas for the related parasite *P. berghei*, which also identified outlier parasites where expression of these genes was observed in other life cycle stages [38]. While scRNA-seq has not yet been applied to *P. yoelii* across its life cycle, it is anticipated those results and their interpretations of those experiments would be largely the same.

## Conclusion

What is apparent is that these promoters are active in stages outside of what they are commonly described to be “specific” for. This can potentially complicate their common applications in promoter swap experiments, or for tightly controlled transgene expression throughout an entire population of parasites. It is therefore recommended that two-component expression systems (split/multiple reporters, Cas9 and sgRNAs, etc.) be used with promoters that overlap in only the intended stage to aid in specificity. For instance, to restrict expression to mid-liver stage, the combined use of the *uis4* and *lisp2* promoters would be advised (Fig. 5). In that context, stage-specific activity could theoretically be achieved as desired.

## Supplementary information

**Additional File 1**: Oligonucleotides used in this study. Lower case letters indicate non-homologous bases added for cloning purposes

**Additional File 2**: Promoter sequences used in this study

**Additional File 3**: Integration of *pyclag-a* promoter and GFPmut2 reporter into the *p230p* safe harbor locus of *Plasmodium yoelii*

**Additional File 4**: Integration of *pydd* promoter and GFPmut2 reporter into the *p230p* safe harbor locus of *Plasmodium yoelii*

**Additional File 5**: Integration of *pylap4* promoter and GFPmut2 reporter into the *p230p* safe harbor locus of *Plasmodium yoelii*

**Additional File 6**: Integration of *pytrap* promoter and GFPmut2 reporter into the *p230p* safe harbor locus of *Plasmodium yoelii*

**Additional File 7**: Integration of *pyuis4* promoter and GFPmut2 reporter into the *p230p* safe harbor locus of *Plasmodium yoelii*

**Additional File 8**: Integration of *pylisp2* promoter and GFPmut2 reporter into the *p230p* safe harbor locus of *Plasmodium yoelii*

**Additional File 9**: Integration of a minimally active *pybip* promoter and GFPmut2 reporter into the *p230p* safe harbor locus of *Plasmodium yoelii*

**Additional File 10**: Integration of a minimal, yet strong, *pybip* promoter and GFPmut2 reporter into the *p230p* safe harbor locus of *Plasmodium yoelii*.

**Additional File 11**: Complete Live Fluorescence and IFA panels for *pyclag-a* promoter::GFPmut2 parasites. Panels provide signals attributed to GFP, stage-defining proteins (ACP, alpha-tubulin, CITH, and CSP), or DAPI. DIC images are also provided. Scale bar lengths are defined within each panel.

**Additional File 12**: Complete Live Fluorescence and IFA panels for *pydd* promoter::GFPmut2 parasites. Panels provide signals attributed to GFP, stage-defining proteins (ACP, alpha-tubulin, CITH, and CSP), or DAPI. DIC images are also provided. Scale bar lengths are defined within each panel

**Additional File 13**: Complete Live Fluorescence and IFA panels for *pylap4* promoter::GFPmut2 parasites. Panels provide signals attributed to GFP, stage-defining proteins (ACP, alpha-tubulin, CITH, and CSP), or DAPI. DIC images are also provided. Scale bar lengths are defined within each panel

**Additional File 14**: Complete Live Fluorescence and IFA panels for *pytrap* promoter::GFPmut2 parasites. Panels provide signals attributed to GFP, stage-defining proteins (ACP, alpha-tubulin, CITH, and CSP), or DAPI. DIC images are also provided. Scale bar lengths are defined within each panel

**Additional File 15**: Complete Live Fluorescence and IFA panels for *pyuis4* promoter::GFPmut2 parasites. Panels provide signals attributed to GFP, stage-defining proteins (ACP, alpha-tubulin, CITH, and CSP), or DAPI. DIC images are also provided. Scale bar lengths are defined within each panel

**Additional File 16**: Complete Live Fluorescence and IFA panels for *pylisp2* promoter::GFPmut2 parasites. Panels provide signals attributed to GFP, stage-defining proteins (ACP, alpha-tubulin, CITH, and CSP), or DAPI. DIC images are also provided. Scale bar lengths are defined within each panel.

**Additional File 17**: Alignments of the promoter sequences between *P. yoelii* and *P. berghei* or *P. chabaudi*. Alignments of these sequences were conducted using the blastn tool embedded in PlasmoDB.org.

## Data Availability

The datasets supporting the conclusions of this article are included within the article and its additional files.
