# Supplementary material for: Definition of constitutive and stage-enriched promoters in the rodent malaria parasite, Plasmodium yoelii
Source: Malar J. 2020 Nov 23;19:424. doi: 10.1186/s12936-020-03498-w (PMC7685602; doi:10.1186/s12936-020-03498-w)
Supplement: Supplementary file 1 — Additional File 1: Oligonucleotides used in this study. Lower case letters indicate non-homologous bases added for cloning purposes [file 12936_2020_3498_MOESM1_ESM.docx]

**Additional File 2: Promoter sequences used in this study.**

**Promoter: *pyclag-a***

**Gene ID: PY17X_1402200**

**Plasmid ID: pSL1058**

**Promoter Sequence (5’ to 3’):** TATATCATGTTTTTACTTGGCTTGTAAACAAATCTTTAGAATGATTAAAGTAGGAAAATATGTTGTCAAATGCTTTTATAAGGGTTAGGTAAAAAATATGATAAGAAAATATAAGTGTAGTTTCTTTTAATCTTTTATTAAAATATATTTTTGTGTTATTAGTTTTTATGGTATAATACAATTTTTATTAGAATGATTTATATGCATAACATGGGGAAATTCATTTCTTTAAAATATTACTGAATACAACATATAGTAAGCATTCAAATATTACCACGATAATTATTAATCCAGTGTTGTAAAGAGTGCGAAGAAGTTTAACCACACAAACCAAGTGCATAAAAAGATTAAGAAAAGTAATAGGGTAAAAACATATATATTGACTAAATTGGTGTTTTAGAAGTATATTTTTTTGTTTTTTTCAAGAATCTAATATGCCATATTGTGTAGGTATTATATATCGATGAGTAATAGTAGCAATTTAACAAAAATGTAATAAAGACGTTTCAAAGTTACACTTCATAGCTACTTCAAAAAAAGAGAAACAACCTGAATTATATAGTATTTTTCTTAGAGTAATTTTTGGGAGGCTTAAGTTTTAGGATTTAGATATGGAATGTTTACAAATATACATAATAAAATAGAAATTTTTTATATAGAAATTATCAAAAAACTTAGTATATAATGAATTATATTTTCTTGAAAAAATAGCATAATAAATATAAATGTGTATTCCAAAACAGAAAAAAAAAACTATATATGAACAAAATTTTATGCGAAAGAATAATTAAATCCCCAAATAAATATTTTTTTATAAGAATAATTATATAATTAAATCATTCGTATATAATGTTATCATAGATATATTACGATTGGAGAAAATATATAATTTTAAATGTGCATGTGTATTATTGTTATGGATGAAAAACGAAAAAAAAAAAAAAAAATTAGATAAAAAGGTTCATATAAATAATAAATAAATTTTAGCCTTAATGAGACCTTTCAATGCAACGTATTAATTAAAATTAGCATATTGTGCACACTTATTGAACACATGAAATGGTATTTGAAAAAATAATATTAGTGATAAATGATAGGGGCTTATTTAAAAAAAATCTATTCAAATTATACTGAAGTTTATGGTAACGTATCCGTATATTGTTAAGGAAATAATTGATAAACACCTAGTGTAAAAAATAATTATTACTTTTTTTCATAATTTATATTTTTAATTTGTTTAATTATATTATATCGACGCTCCATTTATGGTAGGTATGGTTACCATAAATGTGAGAGTCGAAAAAAAAAATATATATATATATTAAAAAATATAGTAAATGCACTAGTGAACGAATATAAAACACACAATTGTCTCGGTGTACTGGTGTATTGAGTAAAAAACGTAGCTACTATTATTTTAAAAAGAAAAAAATAAAGAACATATAGTAATAAATTTAAAGTATCATTTTAAATCATAAATGATATAATAAAAAAAATACACACATATAAAAATATTATTATTTTATATAATAATAAAAGGTTATATACAATTACATGTACATGTGTACCTTATTCTTAATAAAACTTTATAAATAACAATGTATATATAATATAAAATTATTATTTATTCTTATTAAATCATTCTTAAAAAAATTAATAATGCAAAGCTCTATTAATTTTTAATGTGTGTACCATATTTTCACATATAAGTTTCGATATATTTTATGTTTTTGATATTATTGTTTTTGAATTTTAATGGAATTGATATGGCTATAAAAAGATAAAGATCATATATATATTAAAAATTAGGAAAAATG

**Promoter: *pydd***

**Gene ID: PY17X_0418900**

**Plasmid ID: pSL1020**

**Promoter Sequence (5’ to 3’):** TCTTGGTGTTTAATGTATTTATATGTGCATGTTCGCACTTTCTTTTCTATTAATTTTACTGAGATTTATAATTTCTTGTCAATAAAATATAAAAACATATATACACATGATATATTTTTCCTTTTTATAAATCAAAAAAAACTGTCAAATAAATAATTATAATGTATATATATGTTTTATTTATACAAAATTATGTACATATGTATATTTACATTTAAAACCGTATGAATTTTTATAAAACCCTCATGAAATAAGCTTTTAAATTTAATATAATAAATGAACTTATGAGTTTAAATAAAATGGTAATAATTTTTTAAGTAGTACCTTTTAAGTAGTACCTTTTAAATAGTACCTTTTAAATTATTTCTGCATCTAATATGAATAATATTAAAGCTAATAAAAAATAGGAAAATTTTTTATATATATAAAATGGAGACGTGAAATAAAATTATTATGTTCATATAAAAACTGTGATTCGAAAATGTGGGGATAAACACATTTAAAATAGGTACATATATATTTTATTCCTTTTGGGCATTTATTTTTTGTGTTAGCATATAATTATAAAATTTTGTTTTTTTCAAAGTTTGGAAATAAAATGTTGAGATGTGTTTAGAAGCAATGCTAAGTTCCAACGTGCTACATGATGAAATGTTTTAGAAGCAATAATAGGTTCCAACGTGCTGCATGATGAAATAAGGTATGGGTTTGTCTTAAGACACAGTTAGAAGCAGTTTGAATCGTTTCTAAATAATTAAGAAATGAATGAGAATATGCGATTTATTTACATTTTTGCGTATTCACAATCTTATTTATGCACATTTAAATTTTATTGCTTTGATACTTCAAAGAACTTATTCACTACTACTAGTTAAGAATATATATTTTCTCTTAAGTTTATTATAAATGGGAATTTTCCACATTTAGATGTATAAAAAAAAAAAAAAAAATTGGAGAAAAAAAGGGGGTATAATATGTAATAAAATAAGTAGATGTGTACACATAATACATATTTATATATGTGTGTACGTATATGGGAAAGCTATTTTAAAGAAAATAAATTATAAAATGTTTATGCAAGATAATATAAATAAATGGGCATATAATTTCATATAATATATATATATATATTCATTTTTTTATGTGAAAAAGGTTTTAGTACATATAGATATTTGCAAATATATGGGCGAATATATACATTCAGTGTTAAGAGTATAAGTTATTTGGATATGCAATTTGATGGTACATACTATTGTTATATGTATTTCACACATATAGATGCATGTACATATGAATAGGAAGTTTGATTAAATTCAGAATAGATATAAGAATTAATTATCCAAATGAAAAAAAAATG

**Promoter: *pylap4***

**Gene ID: PY17X_1323300**

**Plasmid ID: pSL1019**

**Promoter Sequence (5’ to 3’):** TTAATCCCAAACTTTGATATGTATTTTATCTCTCTATTACTTTATTAAGCTATTTATATAATCCAAGTATATTTTTAATATAAATACGGGGATTATCAATATATAAACACATATTCAAAATGTGTTATTAATTAATTTTTTAATATTTTATAATTAAAAATGAATTAAATAAATACATATGTTCAGAAAATAATCGAAAGACACTAGTTTTAATCAATTAATTTAGCATTTAATTAATAAAGGGATTTTTATTTAATGTTAGATCATTCTATATATAGACATATTGGATTTATTCCAATTAGGGTATTTAAACTTCAAGGTATTTTTAATCATAATACCCGTTTGATGTATATATTTTAAATATGTCATACTATCATAATAATTGCTATAATATCTTATATATTATACTGAATATGTAGACCTTATGTATGCAACTCATGCATTCTACAATTTTTTTGAAAAATACGAATGTAGACATTTAAAGACGTGTTTAATTCAATATAAAAATACGATAAAAAATTGAGAATAATTATAAAATTGTATTAAATTAATTTGATTTTTCTTTTTTAATAATATTTTTTTATAAATATACAACTCTAATAAGATAGCTATGTTTTTTTTTTGTCTTTAATATGTAAAATTATTGTAATAAATATACAAGGTTAAACAATTAATATTTAACAATATTTATACACATATATTCGTGTATGTATATCTACAGTTGAGTATAATACACTTCCACACGCACACATGAAATTAAGATAATTTTATTTTTCTGGCTAGCCATCTACAAATGTATATATAAAAATATAATAAAAAATAGCAAAAGAAATAAAATAAAAGAATAATGGCAATAATAAAAATACTAAATTGTGTGACAATGAAAAATATACTCTATAATATATAAAAATACATGTTATTATAATTAGACAGTTAAAAAAATGTGTGTAACAATAAGTTGATGATACATATGTATATACATATATTCTCAATTTTTATGTGTTTATTTTTTATGCAAACGGAATTTTTAATCGTTCTTTATATATTATATATAATTTAATTTAATTTATTTTATTTTATTATTATTATTATATTAATTTTATTTTTTGTTACCATTTTATGAATATATTACTTACAACGTTTGTTGTTTCAATGTGTACATTTTAGTAGAATATGTGTATACTTTTATAATATTTAAATTATATTATTATTGATATTAATTTTCCAATTGTTATATAAATATTTTTGTGTATAAAATACCTATTTGTCTTAAACTAACAAACAAATATATATATACATATATATATAATTTTTTTCATTTTTTGTATGCGTATATGCAATAAAGTAATTTAATTATTACACTAATTATTATAGTTCTCACTTATGCTACAGTCATTTCTTCACTGAAGCAATATTATTTTATATTTATGTATTACATTTTTTATTAATAAAAAAAATTATG

**Promoter: *pytrap***

**Gene ID: PY17X_1354800**

**Plasmid ID: pSL1082**

**Promoter Sequence (5’ to 3’):** GTTAAATAGGTTTATACTTAAAAATTGCTTTTTAGATTTGCCCCCTTTTTTGTGTTCAAAAAATATACGAATTTTTATTGAATTATATCAACATAAAAATAATGTACATATTATTTATTCCTACATATATATATATTGAGAGAAAATATAGTGGATGTTATTTATGAGTATATATGTATATGTATACGATTTTTCTCTTTTCTCCTTTCCCTTTTCTCTTTTTTTGCTCATTCGCCAAATATTTACACCACTCTTCCTTATTGTAACATTTATACAAGGACACCTTCAAAAAAAATAAATATATGCAAAATATAAATTGAGATAATGAATAAACCGCCAAAAAAACAAGACACAAAAAATAAGCCTATTTTATACAACAAATATATGTATATAAAAATTGTGTAAATTTTTATTTATTCAATGAAGGGCTATATGTAGTTATTTATAATTAATAAAATTATGCATGCAATTGAATACATGTAAAAAAGAGAAACTCCTCCATTTTATAATTTATATAGCTATAAAAAACAAACAAAAAATGGTTAAATGCTAATTTATATATAATATCTCTTTTATTATTTTTTTGTAGATTAATTTCTCTTTCCATATTGCACACATATTAACTTTCTACATGTACATATAATAAATTTTTTTGGGGTTGTAATAATTAGCTCGATATTTTAAACCCTTAATTATATTATTTTTACATGTAACTCATATTAATATATATAATGTCTTCCACAAAATTGATCAAATATATTTCCTAAATATGTTACAATATATATCCATAAAATACATACATGTAATATATTTAATAATAATTTTCTGATATAAATATAGACAGCTTTTTTATATATATTAAAACCATTTAAAAAAGTAAATTTTATAAATTTTGTTTAATTTTCTTTATATATATAATATATATATACATTTATATATACTCTTGTTCTTTTTATCGATTAAAAAAATATATAATATCCATTATATTTATTTTTTAACAATTAAAAATATATAAAATGTACCCCTTGTGCTTGAAGCAACATTTTTTATATTTAACTGTTGTATCTTTTTTTACATATATTTGTTCACATTCTTTGGGATGATATTAAATAATATAATTTTCGAAGAGAAATATTTTTAAATACTTTTTTTAGTGCTTGCATTATTTTTATGATATATATTAACATTCATAAAATATATATTTGTTGAGTGTTGGTTGCCAGTTTATTGAATTAGCTATATTTTTAAATACTAAATATATTTTTTTAAATTGGTTATGATCATATTCTAATCCGTATTATATTGCGTATGTGTATATATATAACGGAAAAAAAGGAAAACATTTAATTTCCTCAGACGCTATTGAATTAAATTAACTATATATCAGTTTTATATAAGAAAAGGTAACACACTCTCTCTCTATATATATATAATTGCAAACGTGTAGACATTTTTATATATGGCCAAATAGTAAATACAAAATAATTCCTCACTTTTATTCTCTTACATATATTATAATACATACATAGACACATAATTTTACCCATTCCCCATTTCTCTTATAGACAGAAACATG

**Promoter: *pyuis4***

**Gene ID: PY17X_0502200**

**Plasmid ID: pSL1083**

**Promoter Sequence (5’ to 3’):** ATAGGTACCCTTCTTTGAGCAAATACTGAACAATAGGGAATGCTTCTATGTAACTGTGGATATATATGTACGTAATAAAATAATTTTTTGTCCATAAAAATATCTTTAACAGCTACTATACAAAAGCAATGAATTGGTTAATACATTTTTGTCGTAAATAAAATAAATGAATTAATGGTACAAAATTACAATAAAAAGGGAGCAAGTTTATTGTATTTTGTTTAATTTATGTCCTTTCCATTTTATTAATACAATTATGATATTCACTAATTCGTATTAAACATATCTTTTGCATGTACCAAAGGTTTGGGGAATAAGCATATGAATCCCATTAAATTTAATGATATTCTGCGATTTTTCTTGTATTTACTATTAAATATAATGGATTCATTTTTTGATGCATGCAATTTTTTCTTTTAATGTATTAATTAGTGTAATAATTTTGTAAAAACTTTATTCATTTATTTATTTTCCCTATTATATTGGGTTCATAATACCATAATTATTATATAAACCACATTAAATAATTTGTAATTTATTCAAGGGTATTAAAAAAAACATATATAAAATGCATATATCCACATATGGTTCATTATAGGATGAATAAAAATGGGAACAACATATGAATTATTATATCATGAAAATAATGAAAAAACATAAATTATATGGATATATACATATATAAATATATTAAAAAATAATAATAAATAAATAAAATGTATTATAAACCTTATAAAATAAGTGATGTTGTGGATAATCCACGAAATATGCCATAAATAGACACTGAACAAATTAGTGGTTCTTAATATTTTTTTGGATACATGCGGATATTACCATTGACAGATGATTTATTTTTTGTTATTTTTAAATTATACATATTCATAGTTTATATAGTCCTCAAAAAATAGGATGTTTTATTCTTTTATAGCTATATTTTATGGTTGATCCTTTCCTTTTATGGTGTTTCATAAAAATTTTATTGAGCTATATATAATCCAATAAAAAAAGGTGATTGAATTTTGAAATATATTAAACTTTTTTTATAATAAAATAAATATAATTATTTTTAAATAATATATATTATATATATATATTATATATTATATATTTTTTTTTATCTTTACACAGAATTTTTTTTATAGAGTCCAATATATATAATTAGTTATATATATACACCACCATAAATAATTATAAGGAAAATCAGTTATTTAAATTTTAACTGAAGAAATTAAATAAGTATATAAAAAAAGAAAAGAACAAAAAATAAAACGACAACAACCTTAAAAATTTTTTATTATTACATATTTATACATAAAAAAAATAAAATACCTAGACAAAAAATAATAAATATTATAGATCGATATTGACTACATATATACCTTTCAGCACATAATTACATCTGAATAAAATG

**Promoter: *pylisp2***

**Gene ID: PY17X_1004400**

**Plasmid ID: pSL1017**

**Promoter Sequence (5’ to 3’):** GTTAATAACCCATCAACATTTTGTTGAATTGTTTTGGATAAAAATGATTGTCATTGTCCTTATGTGCATATAACATCGTTTGATTTAATATATAAATTTAATATAATTTAAACAAACACATATATACATATAGGTGCATGTCTTATAGTTATTCTATAATTTGTAATTACATAGTCATAATTGTTTATTCTTGAAATATTGGTATTTTTTACATTAAAAAAAATATTATGAAAAAAAGTATGTGTATATATATTGTGTAATTTTCCTCAATTTTGGGGAACATGTAAAAACTCTTGGAATTGTATTTTATAAAAATATTATTTTACATAATTTATACTATTGTATATTATTTTTTTATTTTTTAAGTATTTTCGAAATTTTCACATTATTTTATTATGATATGTATATAAAGGTATGTATATATAAATTGTTAAATAGTTCTCACACAAACGATGTTATCAATTTATTAAAGTATATAATAATAAAAAAAAAAAAAAAGTATGATCAGAATAAAACATTCTTATAAATGTTCTTAAACATAAAAAAATGGAATATAAACATTAAAAATATCACATCTTTTAAGTGTTCATTAGCATATACCATTTTTCGTATGATCTTTAAATCTAGAGGTTCTCTTACACATGTGAATGTATATGTATATAATTTTTTAACGATGTAACAGTGTTGTATATATTATTTATATTAAAAAATAATGTAAACAAAATTATAGGAATAAACTAATTTTGAATCATTTATTTGCTTAAAAATTAACCACATTATATTTAATAAATATAAAATAAAAATATGCCACATCCATTCTATACTATTTATTGGTACATCGACATTCACCACAAAATATTTCGTAGAGTTGCATTATCATCAAAAGGGTATAAAATTATAAAATATAACATAAAATATAACATAATGGTGAAACTAAAAATAACGAAATTCAAGTTGGTTGAATAAAATAAAAACACAACATAAAAGATCAAGGTATTATTTTTTTTTAAATATTCTTTATGAAAAACGATTAGAACAATTTAAAATAATGTATATTATGCTTGTACAATATAGTTAAAATAAAGCGAATAACGATACGATTATTCAATATGCAGATTAATAATATATATGCATGTAAATGTGTATATATTTCTTCATTGTCAAATTGCTTAGGCATATGTAGCATATATGATTTTTGACGATTCTTTAAAAATATACAACTGTAATTCCTAATAAATTAATGCAGTTGTAATCGAAAAAAAGGCATGTGCTCCCGTATCGATTTAACCATTTAGGTATTGATCTATGATCGATCTACATGTGAAGAAAATGTGCATTTTTAAAAACAGTTTCATAATGGTTTATGAATTTTTTAGATCATATATTGTTTAAACAAAAAATAAAACAAATACCTATTGTTTGATATTCGATAAAGTGTGCAAACAAATTTGCATGTATGTATTCTATTATCGTTGGTTTTTTTTACACCACCTTTTTTTTTTATTTATAATGTTTTTCACCACAAAGCAACTATATATATAAAACACACTAAAAATATATAATTAAATTAAAAAATATAATAAAGAAAATAAAAAGCATATGTATTATACATACATGTATATTCTCCTGAATATGTATGTTTTCAGTTCAGATTAAGAACAAAAAAATACAGATAATTAAGCATTTATTTGTTACGTTTATACATGTCAATACCAAAAATATATATGTGCCTACACATGTTACATATAAGTAGATACATATAAATATATCGGAGTAATATTTTTCTTCTTTAATTTTTCACTTTTATTATAATCATTTTACTTTTTTACACATAAAAAATG

**Promoter: *pybip-300***

**Gene ID: PY17X_0822200**

**Plasmid ID: pSL1190**

**Promoter Sequence (5’ to 3’):** GCATAGAAGAATTCATACATTGTGCTTAAAAAGAAATATTTAAGTTTTTGTGAAGCTATTCTAAAAAAAACGGATAAATATATATTTTTGTATATTATATATAAAATATGTATATGTAAATATTAAAAAAAATATATATATTATATATTTTTTTTTTGAAATTAATATTAAAGCTATTGTTAAAAAAAAAAAAAAAAAAAGGGAACTTTTATTGGAAATATACATTTTGAATAATTTTTACCTTAGTTTTGAACAAGAATTAATTACATTATTATTTTTATTAATTAATTTTGTATGCATAAAAATTAAAGCAAATG

**Promoter: *pybip-500***

**Gene ID: PY17X_00822200**

**Plasmid ID: pSL1196**

**Promoter Sequence (5’ to 3’):** GCATATTATATCACATATTTTATGAATGTGCATAATATTTATTGCTTGTTCATACAAATATTTTAATATGTGTTCAAAAGTAAGGGATTAGTGAGAGGTAAAAAAAAATACAATTATTTTGATATTGCTTAAAATATAGATATTTTATATAACATTAAAAAAAATTAATAATAACCCAAAAAAAAATAATATATATGCATAGAAGAATTCATACATTGTGCTTAAAAAGAAATATTTAAGTTTTTGTGAAGCTATTCTAAAAAAAACGGATAAATATATATTTTTGTATATTATATATAAAATATGTATATGTAAATATTAAAAAAAATATATATATTATATATTTTTTTTTGAAATTAATATTAAAGCTATTGTTAAAAAAAAAAAAAAAAAAAGGGAACTTTTATTGGAAATATACATTTTGAATAATTTTTACCTTAGTTTTGAACAAGAATTAATTACATTATTATTTTTATTAATTAATTTTGTATGCATAAAAATTAAAGCAAATG

**Promoter: *pbeef1-alpha***

**Gene ID: PBANKA_1133300**

**Plasmid ID: pSL0489**

**Promoter Sequence (5’ to 3’):** AGCTTAATTCTTTTCGAGCTCTTTATGCTTAAGTTTACAATTTAATATTCATACTTTAAGTATTTTTTGTAGTATCCTAGATATTGTGCTTTAAATGCTCACCCCTCAAAGCACCAGTAATATTTTCATCCACTGAAATACCATTAAATTTTCAAAAAAATACTATGCATATAATGTTATACATATAAACATAAAACGCCATGTAAATCAAAAAATATATAAAAATATGTATAAAAATAAATATGCACTAAATATAAGCTAATTATGCATAAAAATTAAAGTGCCCTTTATTAACTAGctagTCGTAATTATTTATATTTCTATGTTATAAAAAAATCCTCATATAATAATATAATTAATATATGTAATGTTTTTTTTATTTTATAATTTTAATATAAAATAATATGTAAATTAATTCAAAAAATAAATATAATTGTTGTGAAACAAAAAACGTAATTTTTTCATTTGCCTTCAAAATTTAAATTTATTTTAATATTTCCTAAAATATATATACTTTGTGTATAAATATATAAAAATATATATTTGCTTATAAATAAATAAAAATTTTATAAAAATG
