# Supplementary material for: Definition of constitutive and stage-enriched promoters in the rodent malaria parasite, Plasmodium yoelii
Source: Malar J. 2020 Nov 23;19:424. doi: 10.1186/s12936-020-03498-w (PMC7685602; doi:10.1186/s12936-020-03498-w)
Supplement: Supplementary file 8 — Additional File 8: Integration of pylisp2 promoter and GFPmut2 reporter into the p230p safe harbor locus of Plasmodium yoelii [file 12936_2020_3498_MOESM8_ESM.pdf]

Additional File 9 - Bowman and Finger *et al.*

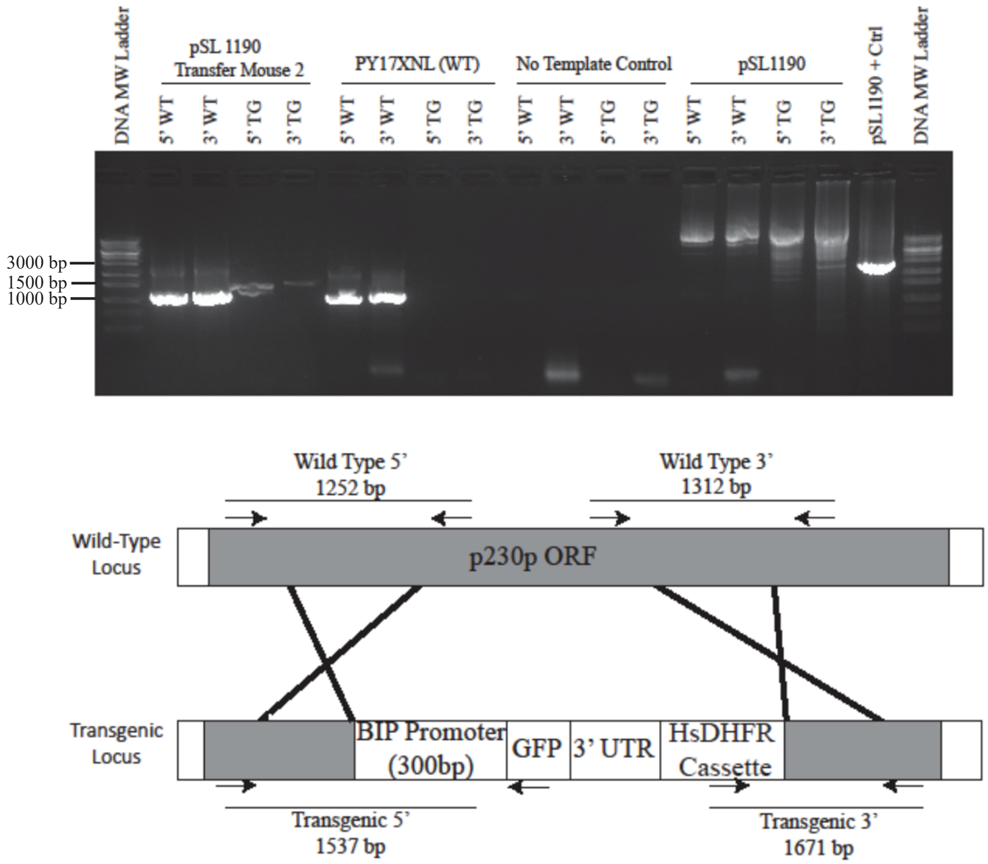

Additional File 9: Integration of a minimally active *pybip* promoter and GFPmut2 reporter into the *p230p* safe harbor locus of *Plasmodium yoelii*. (Top) Genotyping PCR was conducted with one parasite population transfected with the linearized pSL1190 plasmid and wild-type parasites. Primer sets specific to wild-type parasites (WT-5', WT-3') or transgenic parasites (TG-5', TG-3') were used to assess integration on both sides of the *pyp230p* locus. (Bottom) Expected PCR amplicons are noted in the genomic loci schematic.
