# Supplementary figures and images for: Definition of constitutive and stage-enriched promoters in the rodent malaria parasite, Plasmodium yoelii
Source: Malar J. 2020 Nov 23;19:424. doi: 10.1186/s12936-020-03498-w (PMC7685602; doi:10.1186/s12936-020-03498-w)

Additional File 11 - Bowman and Finger *et al.*

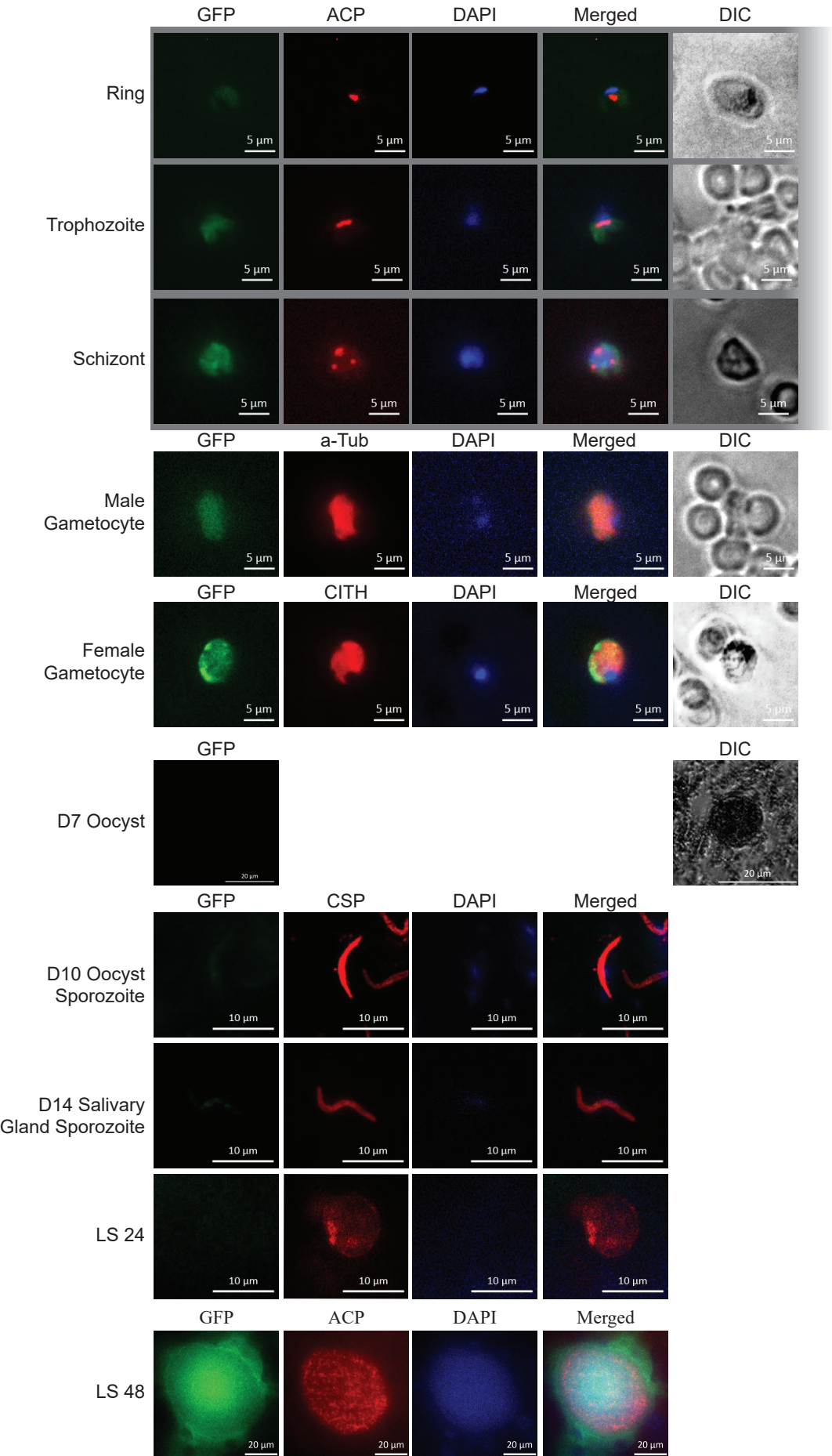

Supplement: Supplementary file 10 — Additional File 10: Integration of a minimal, yet strong, pybip promoter and GFPmut2 reporter into the p230p safe harbor locus of Plasmodium yoelii. [file 12936_2020_3498_MOESM10_ESM.pdf]

Additional File 12 - Bowman and Finger *et al.*

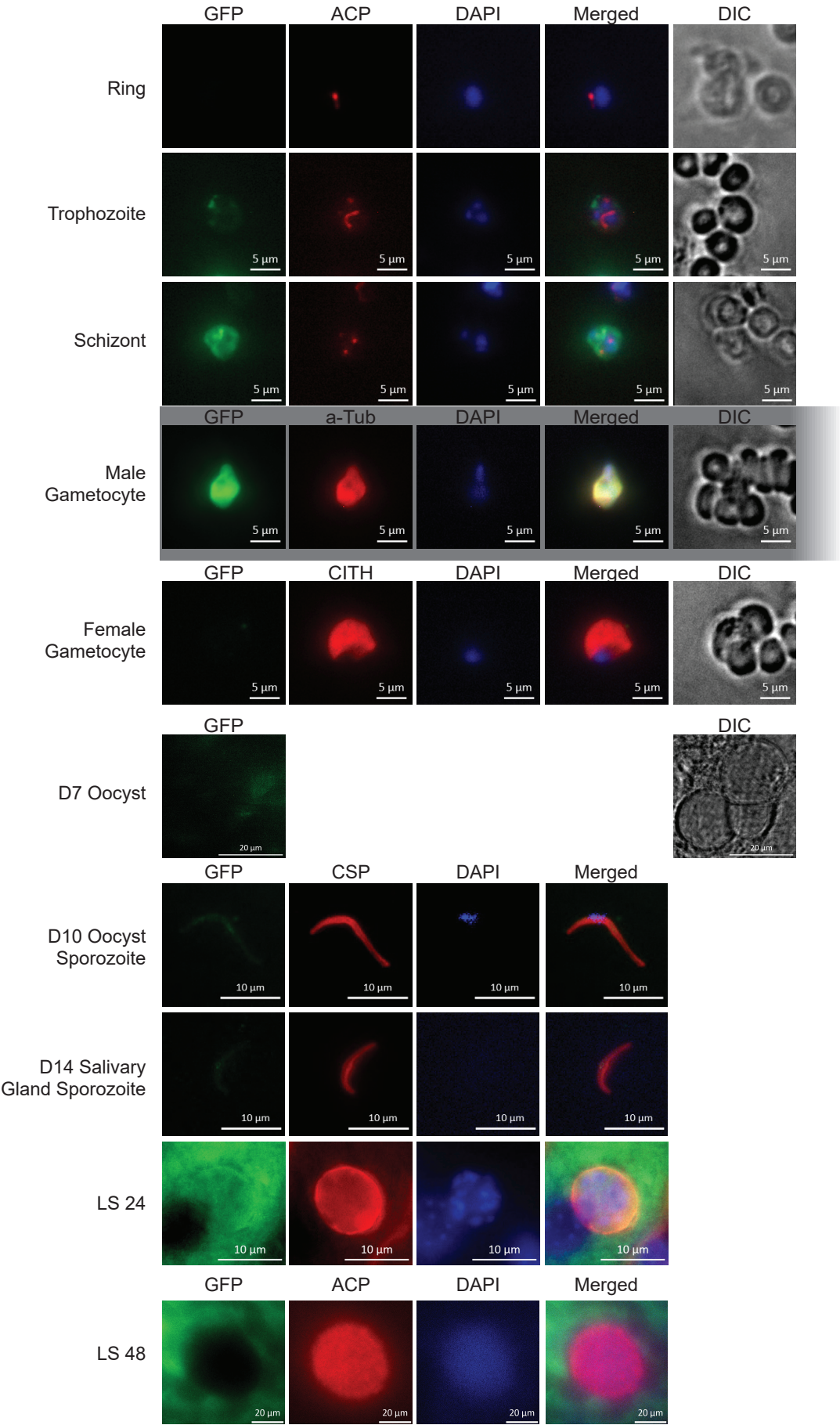

Supplement: Supplementary file 11 — Additional File 11: Complete Live Fluorescence and IFA panels for pyclag-a promoter::GFPmut2 parasites. Panels provide signals attributed to GFP, stage-defining proteins (ACP, alpha-tubulin, CITH, and CSP), or DAPI. DIC images are also provided. Scale bar lengths are defined within each panel. [file 12936_2020_3498_MOESM11_ESM.pdf]

Additional File 13 - Bowman and Finger *et al.*

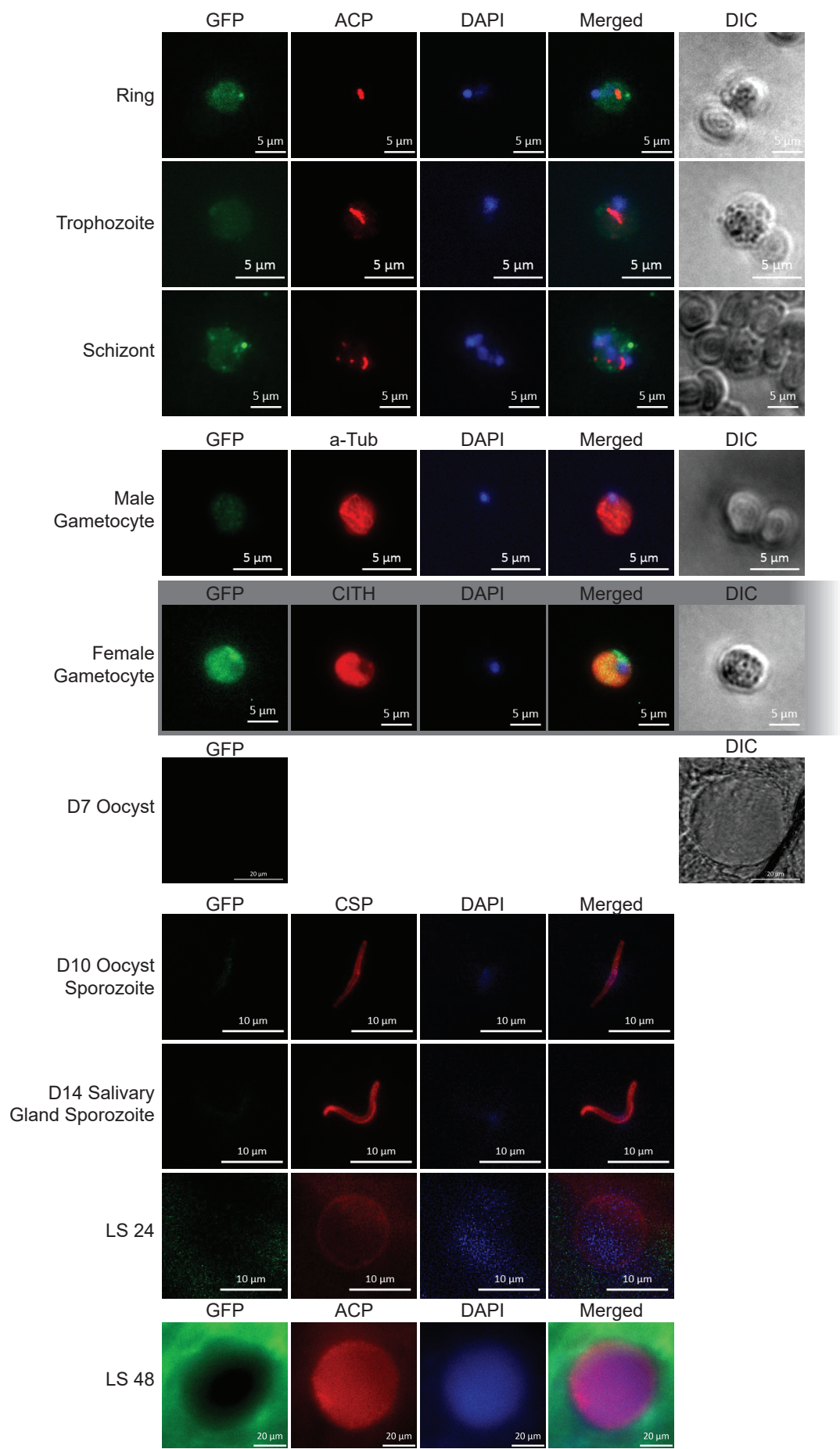

Supplement: Supplementary file 12 — Additional File 12: Complete Live Fluorescence and IFA panels for pydd promoter::GFPmut2 parasites. Panels provide signals attributed to GFP, stage-defining proteins (ACP, alpha-tubulin, CITH, and CSP), or DAPI. DIC images are also provided. Scale bar lengths are defined within each panel [file 12936_2020_3498_MOESM12_ESM.pdf]

Additional File 14 - Bowman and Finger *et al.*

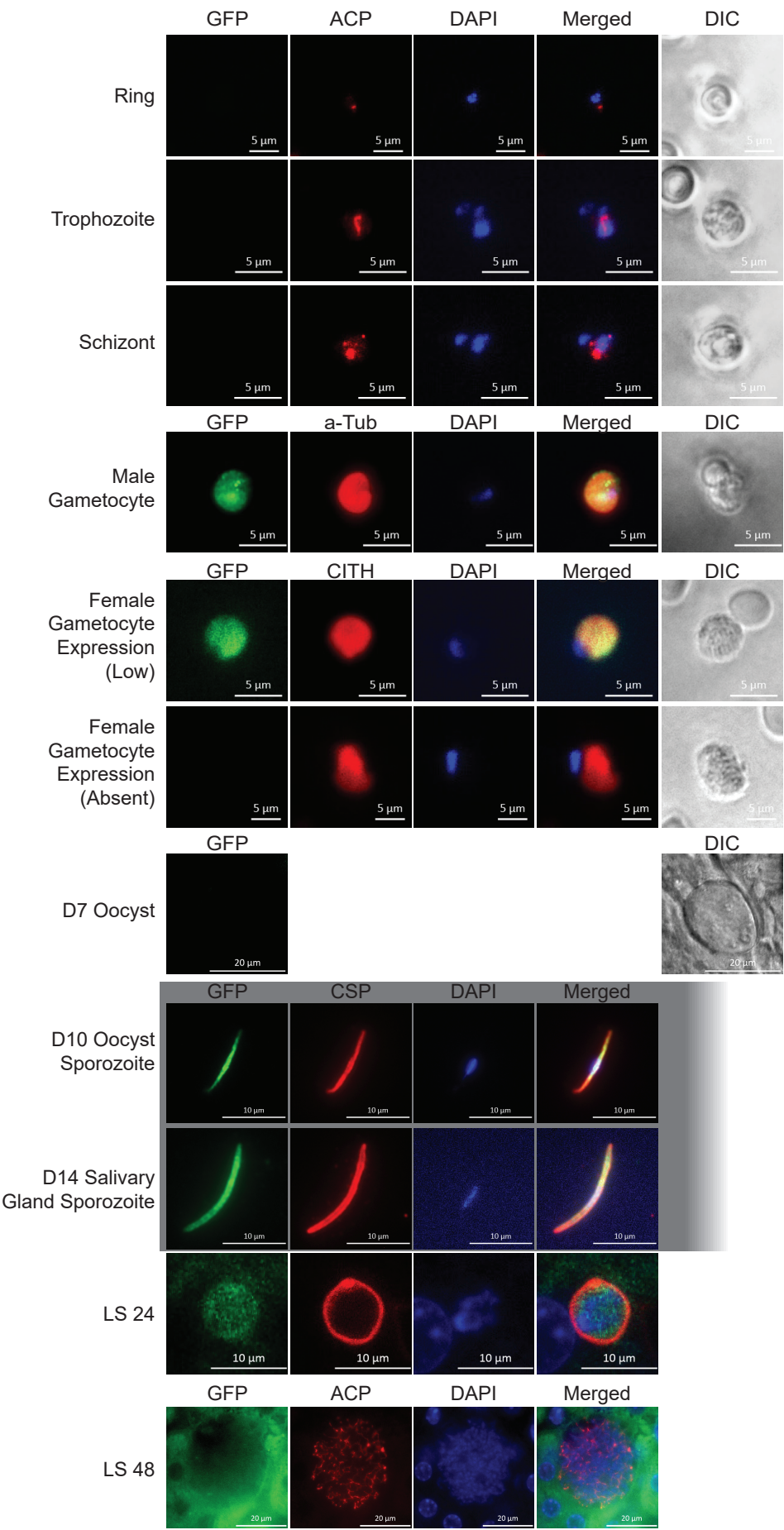

Supplement: Supplementary file 13 — Additional File 13: Complete Live Fluorescence and IFA panels for pylap4 promoter::GFPmut2 parasites. Panels provide signals attributed to GFP, stage-defining proteins (ACP, alpha-tubulin, CITH, and CSP), or DAPI. DIC images are also provided. Scale bar lengths are defined within each panel [file 12936_2020_3498_MOESM13_ESM.pdf]

Additional File 15 - Bowman and Finger *et al.*

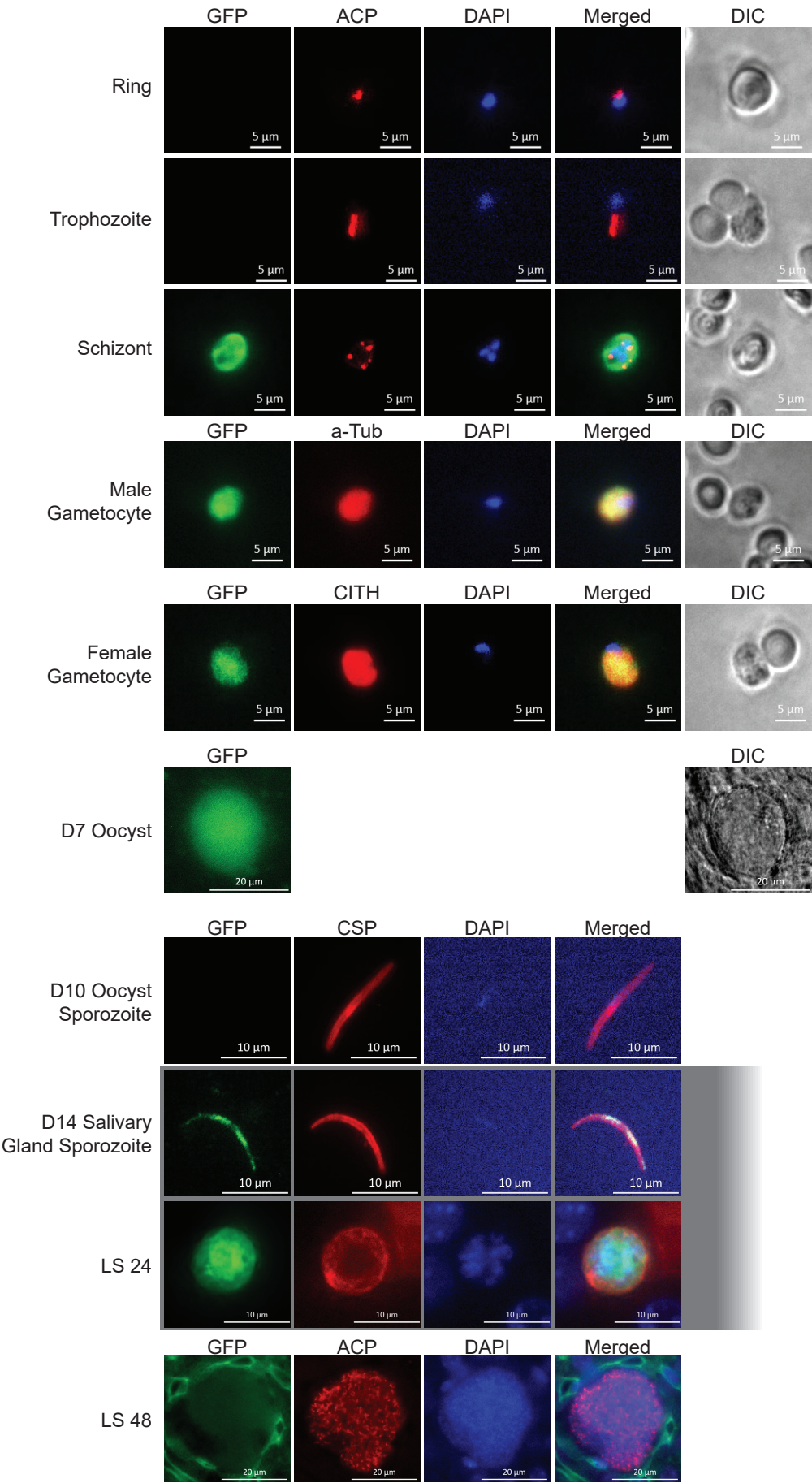

Supplement: Supplementary file 14 — Additional File 14: Complete Live Fluorescence and IFA panels for pytrap promoter::GFPmut2 parasites. Panels provide signals attributed to GFP, stage-defining proteins (ACP, alpha-tubulin, CITH, and CSP), or DAPI. DIC images are also provided. Scale bar lengths are defined within each panel [file 12936_2020_3498_MOESM14_ESM.pdf]

Additional File 16 - Bowman and Finger *et al.*

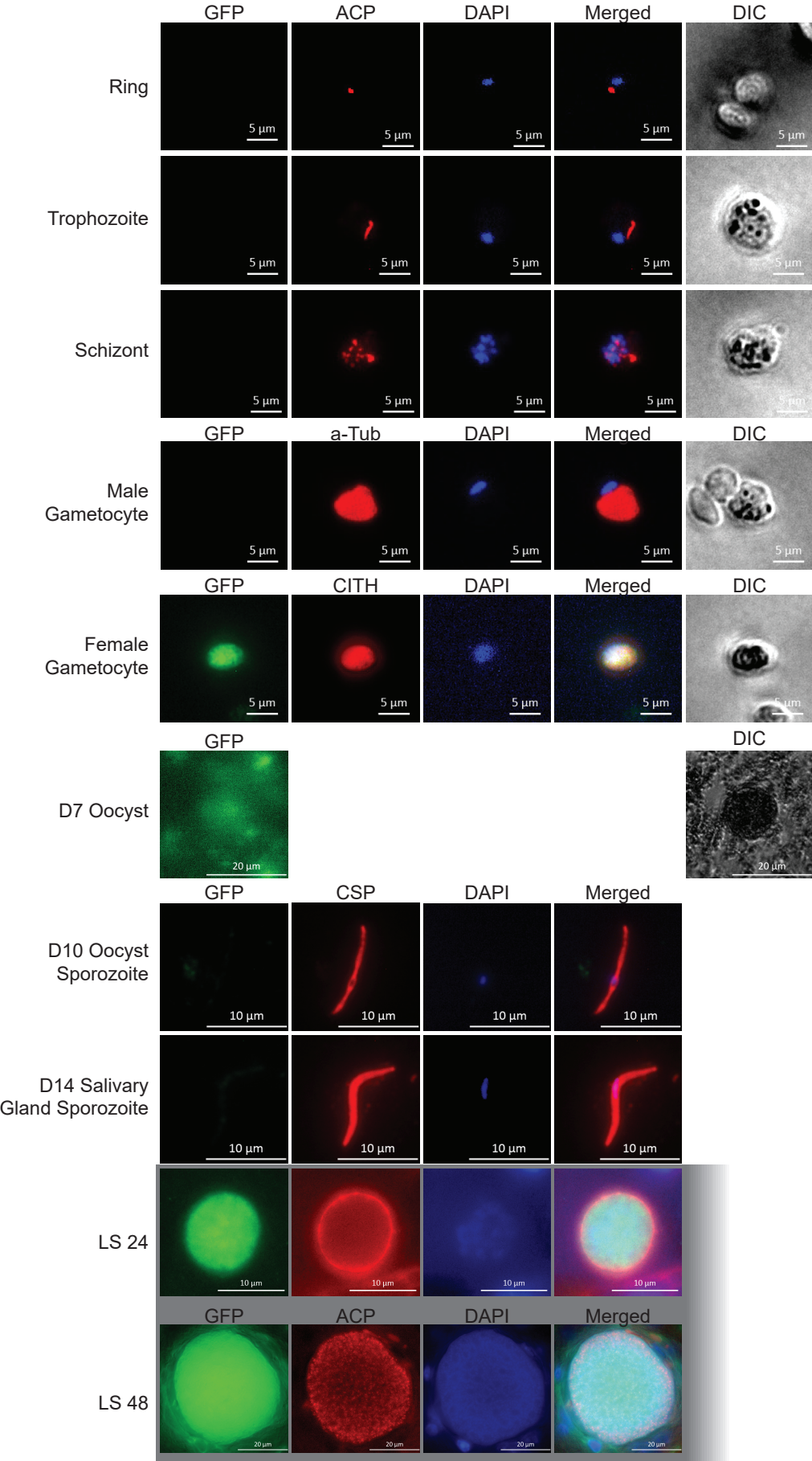

Supplement: Supplementary file 15 — Additional File 15: Complete Live Fluorescence and IFA panels for pyuis4 promoter::GFPmut2 parasites. Panels provide signals attributed to GFP, stage-defining proteins (ACP, alpha-tubulin, CITH, and CSP), or DAPI. DIC images are also provided. Scale bar lengths are defined within each panel [file 12936_2020_3498_MOESM15_ESM.pdf]
