# Supplementary material for: Definition of constitutive and stage-enriched promoters in the rodent malaria parasite, Plasmodium yoelii
Source: Malar J. 2020 Nov 23;19:424. doi: 10.1186/s12936-020-03498-w (PMC7685602; doi:10.1186/s12936-020-03498-w)
Supplement: Supplementary file 16 — Additional File 16: Complete Live Fluorescence and IFA panels for pylisp2 promoter::GFPmut2 parasites. Panels provide signals attributed to GFP, stage-defining proteins (ACP, alpha-tubulin, CITH, and CSP), or DAPI. DIC images are also provided. Scale bar lengths are defined within each panel. [file 12936_2020_3498_MOESM16_ESM.docx]

**Additional File 17: Alignment of Promoter sequences used in this study across
*P. yoelii*, *P. berghei*, and *P. chabaudi***

**Promoter:** *pyclag-a*

**Gene IDs:** PY17X_1402200 (“Query”), PBANKA_1400600, PCHAS_1402500 (all genes are syntenic)

[PbANKA_14_v3](https://plasmodb.org/plasmo/app/record/genomic-sequence/PbANKA_14_v3) | organism=Plasmodium_berghei_ANKA | version=2017-01-09

| length=2549703 | SO=chromosome

Length=2549703

Score = 2495 bits (2766), Expect = 0.0

Identities = 1666/1835 (91%), Gaps = 46/1835 (3%)

[**Link to Genome Browser**](https://plasmodb.org/plasmo/app/jbrowse?data=/plasmo/service/jbrowse/bySequenceId/PbANKA_14_v3/&loc=PbANKA_14_v3:80550-82361&tracks=gene), Strand = Plus/Minus

Query 6 CATGTTTTTACTTGGCTTGTAAACAAATCTTTAGAATGATTAAAGTAGGAAAATATGTTG 65

||||||| ||||||||||| ||| |||||||||||||||||||||||||||||||||||

Sbjct 82361 CATGTTTCTACTTGGCTTGAAAA--AATCTTTAGAATGATTAAAGTAGGAAAATATGTTG 82304

Query 66 TCAAATGCTTTTATAAGGGTTAGGTAAAAAATATGATAAGAAAATATAAGTGTAGTTTCT 125

||||||||||||| |||||||| |||||||||||||||||||||||||||||||||||||

Sbjct 82303 TCAAATGCTTTTAAAAGGGTTATGTAAAAAATATGATAAGAAAATATAAGTGTAGTTTCT 82244

Query 126 TTT-AATCTTTTATTAAAATATATTTTTGTGTTATTAGTTTTTATGGTATAATACAATTT 184

||| ||| ||||||||||||||| |||| |||||||| |||||| || |||||||||||

Sbjct 82243 TTTCAATTTTTTATTAAAATATACTTTTACGTTATTAGCTTTTATTGTGTAATACAATTT 82184

Query 185 TTATTAGAATGATTTATATGCATAACATGGGGAAATTCATTTCTTTAAAATATTACTGAA 244

|||||||||| ||||||||||||||||||||||||| || ||||||||||||| |||||

Sbjct 82183 TTATTAGAATTATTTATATGCATAACATGGGGAAATCCACCTCTTTAAAATATTGCTGAA 82124

Query 245 TACAACATATAGTAA-GCATTCAAATATTACCACGATAATTATTAATCCAGTGTTGTAAA 303

||||| |||| ||| ||||| |||||||| ||||||||||||||||| | |||||||

Sbjct 82123 TACAATATATTGTAGCGCATTAAAATATTATCACGATAATTATTAATCTAACATTGTAAA 82064

Query 304 GAGTGCGAAGAAGTTTAACCACACAAACCAAGTGCATAAAAAGATTAAGAAAAGTAATAG 363

|| | ||| |||||||| ||||||| ||||| | |||||||||||||||| ||||||||

Sbjct 82063 GACTACGAGGAAGTTTAGCCACACAGACCAATTATATAAAAAGATTAAGAACAGTAATAG 82004

Query 364 GGTAAAAACATATATATTGACTAAATTGGTGTTTTAGAAGTATATTTTTTTGTTTTTTTC 423

||||| ||||||||||| | |||||| | | |||||| ||||||||||||||||||||

Sbjct 82003 GGTAAGAACATATATATGGCCTAAATCGCTATTTTAG---TATATTTTTTTGTTTTTTTC 81947

Query 424 AAGAATCTAATATGCCATATTGTGTAGGTA-TTATATATCGATGAGTAATAGTAGCAATT 482

|||||||||||||| |||||||| |||||| |||||||||| | | ||||| ||| |||

Sbjct 81946 AAGAATCTAATATGTCATATTGTATAGGTAGTTATATATCGGTAACTAATATTAGAAATC 81887

Query 483 TAACAAAAATGTAATAAAGACGTTTCAAAGTTACACTTCATAGCTACTTCAAAAAAAGAG 542

| ||||| ||||||||||| |||||||||||||||||||||||||||||||||| | |

Sbjct 81886 GACCAAAA-TGTAATAAAGAAGTTTCAAAGTTACACTTCATAGCTACTTCAAAAAGCGCG 81828

Query 543 AAACAACCTGAATTATATAGTATTTTTCTTAGAGTAATTTTTGGGAGGCTTAAGTTTTAG 602

|| |||||||||||||||||||||||||||| || ||||| | | || ||||||||||

Sbjct 81827 AAGCAACCTGAATTATATAGTATTTTTCTTAAAGCAATTTATTGT--GCGTAAGTTTTAG 81770

Query 603 GATTTAGATATGGAATGTTTACAAATATACATAATAAAATAGAAATTTTTTATATAGAAA 662

| ||||| ||||||||| ||| |||||| ||||||||||||| |||||||||||| ||||

Sbjct 81769 GGTTTAGTTATGGAATGCTTATAAATATGCATAATAAAATAGCAATTTTTTATATTGAAA 81710

Query 663 TTATCAAAAA---ACTTAGTATATAATGAATTATATTTTCTTGAAAAAATAGCATAATAA 719

|||||||||| || |||||| ||||| |||||||||| |||||| | ||| ||| |||

Sbjct 81709 TTATCAAAAACTTACATAGTATGTAATGTATTATATTTTATTGAAATA-TAGGATACTAA 81651

Query 720 ATATAAATGTGTATTCCAAAACAGAAAAAAAAAACTATATATGAACAAAATTTTATGCGA 779

|||||||||||||| ||||||||||||||||| |||||||||||||||||| ||| |

Sbjct 81650 -TATAAATGTGTATTTCAAAACAGAAAAAAAAAT-TATATATGAACAAAATTTCATGTGG 81593

Query 780 AAGAATAATTAAATCCCCAAATAAATATTTTTTTATAAGAATAATTATATAATTAAATCA 839

|| ||||||||||||||||||||||||||||||||||||| ||||||||||||||||||

Sbjct 81592 AAAAATAATTAAATCCCCAAATAAATATTTTTTTATAAGAGCAATTATATAATTAAATCA 81533

Query 840 TTCGTATATAATGTTATCATAGATATATTACGATTGGAGAAAATATATAATTTTAAATGT 899

| ||||||||| |||||||||||||||||||||||||||||||||||| |||||||||||

Sbjct 81532 TCCGTATATAA-GTTATCATAGATATATTACGATTGGAGAAAATATATGATTTTAAATGT 81474

Query 900 GCATGTGTATTATTGTTATGGATGAAAAACGAAAAAAAAAAAAAAAAATTAGATAAAAAG 959

||||||||||||||||||||||||||||||||||||| ||| ||||||||||||||||||

Sbjct 81473 GCATGTGTATTATTGTTATGGATGAAAAACGAAAAAATAAATAAAAAATTAGATAAAAAG 81414

Query 960 GTT-CATATAAATAATAAATAAATTTTAGCCTTAATGAGACCTTTCAATGCAACGTATTA 1018

||| |||| ||||||||||||||||||||||||||||||||||||||||||||| ||||

Sbjct 81413 GTTACATAAGAATAATAAATAAATTTTAGCCTTAATGAGACCTTTCAATGCAACGCATTA 81354

Query 1019 ATTAAAATTAGCATATTGTGCACACTTATTGAACACATGAAATGGTATTTGAAAAAATAA 1078

|||||| ||| ||||||||||||| ||||||||||| ||||||||||||||||||||||

Sbjct 81353 ATTAAACTTACCATATTGTGCACAATTATTGAACACGGGAAATGGTATTTGAAAAAATAA 81294

Query 1079 TATTAGTGATAAATGATAGGGGCTTATTTAAAAAAAATCTATTCAAATTATACTGAAGTT 1138

||||||| |||||| ||||| | ||||| |||||| ||||||||||||||||| ||||||

Sbjct 81293 TATTAGTAATAAATCATAGGAGATTATT-AAAAAAGATCTATTCAAATTATACAGAAGTT 81235

Query 1139 TATGGTAACGTATCCGTATATTGTTAAGGAAATAATTGATAAACACCTAGTGTAAAAAAT 1198

||| |||||||||||| ||||||||||||||| |||||||||||||||||||||||||||

Sbjct 81234 TATAGTAACGTATCCGCATATTGTTAAGGAAAGAATTGATAAACACCTAGTGTAAAAAAT 81175

Query 1199 AATTATTACTTTTTTTCATAATTTATATTTTTAATTTGTTTAATTATATT---------- 1248

|||||||||||||| ||||||||||||||||||| |||||||||||||||

Sbjct 81174 AATTATTACTTTTTCTCATAATTTATATTTTTAACTTGTTTAATTATATTTCGTTTTATA 81115

Query 1249 --ATATCGACGCTCCATTTATGGTAGGTATGGTTACCATAAATGTGAGAGTCGAAAAAAA 1306

||||||||||||||||||||||||| ||||||||||||||||||||||||||||||||

Sbjct 81114 TTATATCGACGCTCCATTTATGGTAGGCATGGTTACCATAAATGTGAGAGTCGAAAAAAA 81055

Query 1307 AA--ATATATATATATATTAAAAAATATAGTAAATGCACTAGTGAACGAATATAAAACAC 1364

|| |||||||||||||||||||| ||||||||||||||||||||| |||||||||||||

Sbjct 81054 AATTATATATATATATATTAAAAACTATAGTAAATGCACTAGTGAAAGAATATAAAACAC 80995

Query 1365 ACAATTGTCTCGGTGTACTGGTGTATTGAGTAAAAAA-CGTAGCTACTATTATTTTAAAA 1423

||||||||||||||||| |||| ||||||| ||||||||||||||||||||||

Sbjct 80994 ACAATTGTCTCGGTGTA--------TTGAATAAAAAAACGTAGCTACTATTATTTTAAAA 80943

Query 1424 AGAAAAAAATAAAGAACATATAGTAATAAATTTAAAGTATCATTTTAAATCATAAATGAT 1483

||||||||||||||||||||||||||||||||||||||||||||||||||||||||||||

Sbjct 80942 AGAAAAAAATAAAGAACATATAGTAATAAATTTAAAGTATCATTTTAAATCATAAATGAT 80883

Query 1484 ATAATAAAAAAAATACACACATATAAAAATATTATTATTTTATATAATAATAAAAGGTTA 1543

|||||||||||| ||||||||||||||||||||||||||||||||||||||||||||||

Sbjct 80882 ATAATAAAAAAAC-ACACACATATAAAAATATTATTATTTTATATAATAATAAAAGGTTA 80824

Query 1544 TAT-ACAATTACATGTACATGTGTACCTTATTCTTAATAAAACTTTATAAATAACAATGT 1602

||| ||| ||| ||||||||||||||||||||||||||||||||||||||||||||||||

Sbjct 80823 TATTACATTTATATGTACATGTGTACCTTATTCTTAATAAAACTTTATAAATAACAATGT 80764

Query 1603 ATATATAATATAAAATTATTATTTATTCTTATTAAATCATTCTTAAAAAAATTAATAATG 1662

||| || |||||||||| |||||||||||||||||| ||||||||| |||||||||||||

Sbjct 80763 ATACATGATATAAAATTCTTATTTATTCTTATTAAACCATTCTTAACAAAATTAATAATG 80704

Query 1663 CAAAGCTCTATTAATTTTTAATGTGTGTACCATATTTTCACATATAAGTTTCGATATATT 1722

|||||| |||||| ||||||||||||||||||||||||||||||||||||||||||||||

Sbjct 80703 CAAAGCCCTATTACTTTTTAATGTGTGTACCATATTTTCACATATAAGTTTCGATATATT 80644

Query 1723 TTATGTTTTTGATATTATTGTTTTTGAATTTTAATGGAATTGATATGGCTATAAAAAGAT 1782

|||||||||||||||||||||||||||||||||||||||||||||||||| | ||| |||

Sbjct 80643 TTATGTTTTTGATATTATTGTTTTTGAATTTTAATGGAATTGATATGGCTGT-AAAGGAT 80585

Query 1783 AAAGATCATATATATATTAAAAATTAGGAAAAATG 1817

|||||| ||||||||||||||||||||||||||||

Sbjct 80584 AAAGATAATATATATATTAAAAATTAGGAAAAATG 80550

> [PCHAS_14_v3](https://plasmodb.org/plasmo/app/record/genomic-sequence/PCHAS_14_v3) | organism=Plasmodium_chabaudi_chabaudi | version=2015-06-18

| length=2516057 | SO=chromosome

Length=2516057

Score = 1817 bits (2014), Expect = 0.0

Identities = 1535/1831 (84%), Gaps = 97/1831 (5%)

[**Link to Genome Browser**](https://plasmodb.org/plasmo/app/jbrowse?data=/plasmo/service/jbrowse/bySequenceId/PCHAS_14_v3/&loc=PCHAS_14_v3:105575-107327&tracks=gene), Strand = Plus/Minus

Query 6 CATGTTTTTACTTGGCTTGTAAACAAATCTTTAGAATGATTAAAGTAGGAAAATATGTTG 65

|| |||||||| | ||||||||| ||| ||||||||||||||| ||||||||||||| ||

Sbjct 107327 CACGTTTTTACATAGCTTGTAAAAAAAACTTTAGAATGATTAA-GTAGGAAAATATGGTG 107269

Query 66 TCAAATGCTTTTATAAGGGTTAGGTAAAAAATATGATAAGAAAATATAAGTGTAGTTTCT 125

||||||| |||||||||||||| ||||||| |||||||||||||||| ||| | |

Sbjct 107268 TCAAATGGTTTTATAAGGGTTATGTAAAAAGTATGATAAGAAAATATTAGTCC----TTT 107213

Query 126 TTTAATCTTTTATTAAAATATATTTTTGTGTTATTAGTTTTTATGGTATAATACAATTTT 185

|||||| ||||| ||| |||| |||| | ||||||||||||||||| |||||| |||||

Sbjct 107212 TTTAATTTTTTACGAAA-TATAATTTTATATTATTAGTTTTTATGGTGTAATACGATTTT 107154

Query 186 TATTAGAATGATTTATATGCATAACATGGGGAAATTCATTTCTTTAAAATATTACTGAAT 245

||||| ||| ||||||||||| ||||||||||||| || ||||||||||||| |||||

Sbjct 107153 TATTAAAATTATTTATATGCACAACATGGGGAAATA-ATATCTTTAAAATATTGATGAAT 107095

Query 246 ACAACATATAGTAA-GCATTCAAATATTACCACGATAATTATTAATCCAGTGTTGTAAAG 304

||| |||||||| | | ||| |||| ||| ||||||||||||||||| || |||||||||

Sbjct 107094 ACAGCATATAGTGATGTATTAAAATGTTATCACGATAATTATTAATCTAGCGTTGTAAAG 107035

Query 305 AGTGCGAAGAAGTTTAACCACACAAACCAAGTGCATAAAAAGATTAAGAAAAGTAATAGG 364

||| |||| |||||| |||||||||| | || |||||||||||| ||||| |||||||

Sbjct 107034 AGTAAGAAGGAGTTTAGCCACACAAACTAGGTATATAAAAAGATTAGGAAAAATAATAGG 106975

Query 365 GTAAAAACATATATATTGACTAAATTGGTGTTTTAGAAGTATATTTTTTTGTTTTTTTCA 424

| | |||||||| || || ||||||| ||||||||| |||||||||||| |||||

Sbjct 106974 GCAGGAACATATAAATGGATTAAATTGCTGTTTTAGATGTATATTTTTTT-----TTTCA 106920

Query 425 AGA-ATCTAATATGCCATATTGTGTAGGTA-TTATATATCGATGAGTAATAGTAGCAATT 482

| |||||||||||| ||||||||| ||| ||||||| ||||||||| || ||||||||

Sbjct 106919 ATCGATCTAATATGCCGTATTGTGTAAGTAGTTATATAACGATGAGTATTA-TAGCAATT 106861

Query 483 TAACAAAAATGTAATAAAGACGTTTCAAAGTTACACTTCATAGCTACTTCAAAAAAAGAG 542

||||||||||||||||||| ||||||||| |||| ||| || ||||||||||||| | |

Sbjct 106860 CAACAAAAATGTAATAAAGAAGTTTCAAAGATACAGTTCGTAACTACTTCAAAAAACGCG 106801

Query 543 AAACAACCTGAATTATATAGTATTTTTCTTAGAGTAATTTTTGGGAGGCTTAAGTTTTAG 602

||| | ||||||||||||||||||||||||||| ||| ||| | || ||||||||||

Sbjct 106800 AAATACCCTGAATTATATAGTATTTTTCTTAGAACAATCTTTTTGTGGTGTAAGTTTTAG 106741

Query 603 GATTTAGATATGGAATGTTTACAAATATACATAATAAAATAGAA---ATTTTTTATATAG 659

|||||| | || |||| |||| |||||| ||||||||| | || |||||||||| |

Sbjct 106740 GATTTA-AAATAGAAT-TTTATAAATAT-GATAATAAAAAAAAATATATTTTTTATACTG 106684

Query 660 AAATTATCAAAAA----ACTTAGTATATAATGAATTATATTTTCTTGAAAAAATAGCATA 715

||||| ||||||| || || ||||||||| |||||||| ||||||||||||| ||||

Sbjct 106683 AAATTGTCAAAAAAAATACATAATATATAATGCATTATATTCTCTTGAAAAAATA-CATA 106625

Query 716 ATAAATATAAATGTGTATTCCAAAACAGAAAAAAAAAACTATATATGAACAAAATTTTAT 775

||||||| ||||| |||||||| ||||||||||||||| || ||||||||||

Sbjct 106624 ATAAATA------CGTATTTCAAAACAG---AAAAAAACTATATATAAATAAAATTTTAT 106574

Query 776 GCGAAAGAATAATTAAATCCCCAAATAAATATTTTTTTATAAGAATAATTATATAATTAA 835

| |||| |||||||||||| ||||||| ||||||||||||||| |||| |||||||||

Sbjct 106573 GGGAAAAAATAATTAAATCGCCAAATATATATTTTTTTATAAGGGCAATT-TATAATTAA 106515

Query 836 ATCATTCGTATATAATGTTATCATAGATATATTACGATTGGAGAAAATATAT-AATTTTA 894

|||||| |||||| || |||| |||||||||||| |||||||||||||||| | |||||

Sbjct 106514 ATCATTTGTATAT-ATATTAT-TTAGATATATTACAATTGGAGAAAATATATGATTTTTA 106457

Query 895 AATGTGCATGTGTATTATTGTTATGGATGAAAAACGAAAAAAAAAAAAAAAAATTAGATA 954

||||||||||||||||| ||||| ||||| ||||| |||||||| || || ||| |||

Sbjct 106456 AATGTGCATGTGTATTA-TGTTA--GATGATAAACG--AAAAAAAATAATAATTTAAATA 106402

Query 955 AAAAGGTTCATATAAATAATAAATAAATTTTAGCCTTAATGAGACCTTTCAATGCAACGT 1014

||||||| | |||||| |||||||||| ||||||||| | |||||||||||||

Sbjct 106401 AAAAGGT-----TTAATAAT-AATAAATTTTTTCCTTAATGA-AATTTTCAATGCAACGC 106349

Query 1015 ATTAATTAAAATTAGCATATTGTGCACACTTATTGAACACATGAAATGGTATTTGAAAAA 1074

|||||||||||||||||||||||||||| |||||||||| || || | ||||||||||

Sbjct 106348 ATTAATTAAAATTAGCATATTGTGCACA-ATATTGAACACGGGAGATAGCATTTGAAAAA 106290

Query 1075 ATAATATTAGTGATAAATGATAGGGGCTTATTTAAAAAAA-ATCTATTCAAATTATACTG 1133

||| |||| ||||||||||||| | ||||||||||||| | ||||||||||| |

Sbjct 106289 ATATTATT-GTGATAAATGATA---GATTATTTAAAAAAAGAGCTATTCAAATTGCCCAT 106234

Query 1134 AAGTTTATGGTAACGTATCCGTATATTGTTAAGGAAATAATTGATAAACACCTAGTGTAA 1193

| |||||| |||||||||| ||| ||||||||||||| |||||| ||||| |||| |

Sbjct 106233 ATGTTTAT-GTAACGTATCTGTA-ATTGTTAAGGAAAAAATTGAAAAACA--GTGTGT-A 106179

Query 1194 AAAATAATTATTACTTTTTTTCATAATTTATATTTTTAATT-TGTTT-AATTATATTATA 1251

||||||||||||| ||||| || |||| | ||||||||||| | ||| ||||||| |||

Sbjct 106178 AAAATAATTATTAGTTTTTCTCGTAATCTGTATTTTTAATTATATTTCTATTATATAATA 106119

Query 1252 TCGACGCT-CCATTTATGGTAGGTATGGTTACCATAAATGTGAGAGTCGAAAAAAAAAAT 1310

|||||||| || || | | |||||| || |||| ||||| | ||||||||||||| |

Sbjct 106118 TCGACGCTAACACTT-TTGAAGGTATACTT-GCATATATGTG-GGGTCGAAAAAAAAATT 106062

Query 1311 ATATATATATATTAAAAAATATAGTAAATGCACTAGTGAACGAATATAAAACACACAATT 1370

|||||| ||||| ||||||||||| |||| |||||||||||||||||||

Sbjct 106061 ATATAT------------ATATACTAAATGCACTAATGAAAGAATATAAAACACACAATT 106014

Query 1371 GTCTCGGTGTACTGGTGTATTGAGTAAAAAACGTAGCTACTATTATTTTAAAAAG-AAAA 1429

|| |||||| | | |||||||| ||||||||||||||||||||||||||||||| ||||

Sbjct 106013 GTTTCGGTGAATGGATGTATTGAATAAAAAACGTAGCTACTATTATTTTAAAAAGAAAAA 105954

Query 1430 AAATAAAGAACATATAGTAATAAATTTAAAGTATCATTTTAAATCATAAATGATATAAT- 1488

|||||||||||| |||||||| ||||||| ||| ||||||| | | |||| ||||||

Sbjct 105953 AAATAAAGAACA-ATAGTAAT-AATTTAA--TATTGTTTTAAAAC-TTAATGTTATAATA 105899

Query 1489 AAAAAAAATACACACATATAAAAATATTATTATTTTATATAATAATAAAAGGTTATA-TA 1547

|||||||||||||||| ||||||||||||||||||||||||||||||||||||||| ||

Sbjct 105898 AAAAAAAATACACACA-CTAAAAATATTATTATTTTATATAATAATAAAAGGTTATATTA 105840

Query 1548 CAATTACATGTACATGTGTACCTTATTCTTAATAAAACTTTATAAATAACAATGTATATA 1607

|||||| |||||||||||||| ||||||||||||||| |||||||||||||||||| |

Sbjct 105839 CAATTATATGTACATGTGTACTATATTCTTAATAAAACCTTATAAATAACAATGTATGCA 105780

Query 1608 TAATATAAAATTATTATT-TATTCTTATTAAATCATTCTTAAAAAAATTAATAATGCAAA 1666

| |||||| ||||||||| ||| || ||||| | || |||||||||||||| ||

Sbjct 105779 TGATATAATATTATTATTAAATTATTCTTAAAAAA-----AATAAAATTAATAATGCGAA 105725

Query 1667 GCTCTATTAATTTTTAATGTGTGTACCATATTTTCACATATAAGTTTCGATATATTTTAT 1726

||||||||| ||||||||||||||| | ||||||||||||||| |||||||| |||||||

Sbjct 105724 GCTCTATTACTTTTTAATGTGTGTATCTTATTTTCACATATAATTTTCGATAGATTTTAT 105665

Query 1727 GTTTTTGATATTATTGTTTTTGAATTTTAATGGAATTGATATGGCTATAAAAAGATAAAG 1786

|||||||| ||||||| ||||| |||||| | |||||||||||| |||||||||||||

Sbjct 105664 GTTTTTGAAATTATTG-TTTTGTATTTTAGTTGAATTGATATGGAAGTAAAAAGATAAAG 105606

Query 1787 ATCATATATATATTAAAAATTAGGAAAAATG 1817

||||||| |||||||||||||||||||||||

Sbjct 105605 ATCATATGTATATTAAAAATTAGGAAAAATG 105575

**Promoter:** *pydd*

**Gene IDs:** PY17X_0418900 (“Query”), PBANKA_0416100, PCHAS_0417000 (all genes are syntenic)

> [PbANKA_04_v3](https://plasmodb.org/plasmo/app/record/genomic-sequence/PbANKA_04_v3) | organism=Plasmodium_berghei_ANKA | version=2017-01-09

| length=712327 | SO=chromosome

Length=712327

Score = 1496 bits (1658), Expect = 0.0

Identities = 1161/1402 (83%), Gaps = 130/1402 (9%)

[**Link to Genome Browser**](https://plasmodb.org/plasmo/app/jbrowse?data=/plasmo/service/jbrowse/bySequenceId/PbANKA_04_v3/&loc=PbANKA_04_v3:577658-578979&tracks=gene), Strand = Plus/Plus

Query 3 TTGGTGTTTAATGTATTTATATGTGCATGTTCGCACTTTCTTTTCTATTAATTTTACTGA 62

||||||||||||||||||||||||||||| | |||| | |||||||||||||||| | ||

Sbjct 577658 TTGGTGTTTAATGTATTTATATGTGCATGCTTGCACCTACTTTTCTATTAATTTTCC-GA 577716

Query 63 GATTTATAATTTCTTGTCAATAAAATATAAAAACATATATACACATGATATATTTTTCCT 122

| |||||||||||||||||||||||||||||||||||||||||||||||| |||||||||

Sbjct 577717 GCTTTATAATTTCTTGTCAATAAAATATAAAAACATATATACACATGATAAATTTTTCCT 577776

Query 123 TTTTATAAATCAAAAAAAACTGTCAAATAAATAATTATAATGTATATATATGTTTTATTT 182

||| ||||||| |||||||||||||||||||||||||||||||||||||||||||||

Sbjct 577777 TTTCATAAATCCTTCAAAACTGTCAAATAAATAATTATAATGTATATATATGTTTTATTT 577836

Query 183 ATACAAAATTATGTACATATGTATATTTACATTTAAAACCGTATGAATTTTTATA-AAAC 241

|||||||||||||| ||||| |||||||||||||||||||| ||||||||||||| ||||

Sbjct 577837 ATACAAAATTATGTGCATATATATATTTACATTTAAAACCGAATGAATTTTTATATAAAC 577896

Query 242 CCTCATGAAATAAGCTTTTAAATTTAATATAATAAATGAACTTATGAGTTTAAATAAAAT 301

|||||| ||||||||||||||||||||||||||||| | ||| |||||| |||||||||

Sbjct 577897 CCTCATAAAATAAGCTTTTAAATTTAATATAATAAAGGGGCTTGTGAGTT-AAATAAAAT 577955

Query 302 GGTAATAATTTTTTAAGTAGTACCTTTTAAGTAGTACCTTTTAAATAGTACCTTTTAAAT 361

||||| | ||||||| ||||||||||||||||

Sbjct 577956 GGTAACACATTTTTAA----------------------------ATAGTACCTTTTAAAT 577987

Query 362 TATTTCTGCATCTAATATGAATAATATTAAAGCTAATAAAAAATAGGAAAATT-TTTTAT 420

|||||||| ||||| ||| ||||||||||||| |||||| ||||||||||| ||||||

Sbjct 577988 TATTTCTGTATCTAGTATAAATAATATTAAAGGTAATAAGAAATAGGAAAAAAATTTTAT 578047

Query 421 ATATATAAAATGGAGACGTGAAATAAAATTATTATGTTCATATAAAAACTGTGATTCGAA 480

||||||||||||| ||||||||||||||||| ||||||||||||||| |||| ||||||

Sbjct 578048 ATATATAAAATGGGGACGTGAAATAAAATTAACATGTTCATATAAAAATTGTGGTTCGAA 578107

Query 481 AATGTGGGGATAAACACATTTAAAATAGGTACATA-TATATTTTATTCCTTTTGGGCATT 539

||| |||| |||||| ||||||||||||||||||| |||||||||||| ||||||| |||

Sbjct 578108 AATTTGGGTATAAACGCATTTAAAATAGGTACATAATATATTTTATTCGTTTTGGGTATT 578167

Query 540 TATTTTTTGT--GTTAGCATATAATTATAAAATTTTGTTTTTT---TCAAAGTTTGGAAA 594

|||||||| ||||| |||||||||||||||| | |||||| |||||||||| ||

Sbjct 578168 TATTTTTTTATGGTTAGTATATAATTATAAAATTGTTTTTTTTCATTCAAAGTTTGTAAG 578227

Query 595 TAAAATGTTGAGATGTGTTTAGAAGCAATGCTAAGTTCCAACGTGCTACATGATGAAATG 654

||||||||||||||||

Sbjct 578228 TAAAATGTTGAGATGT-------------------------------------------- 578243

Query 655 TTTTAGAAGCAATAATAGGTTCCAACGTGCTGCATGATGAAATAAGGTATGGGTTTGTCT 714

||||||| |||||||| | | | | |||| | ||| ||||| | |||||||||||

Sbjct 578244 -TTTAGAAACAATAATAAGCTTCGAAATGCTAAACTTTGAGATAAGATGTGGGTTTGTCT 578302

Query 715 TAAGACACAGTTAGAAGCAGTTTGAATCGTTTCTAAATAATTAAGAAATGAATGAGAATA 774

|||||||| |||||||||||| ||| |||||||||||||||||||||||||||| ||||

Sbjct 578303 TAAGACACGATTAGAAGCAGTTCGAACCGTTTCTAAATAATTAAGAAATGAATGATAATA 578362

Query 775 TGCG-ATTTATTTACATTTTTGCGTATTCACAATCTTATTTATGCACATTTAAATTTTAT 833

|||| |||||||| ||||||||||||||||||||||||||||||||||||||||||||||

Sbjct 578363 TGCGTATTTATTTGCATTTTTGCGTATTCACAATCTTATTTATGCACATTTAAATTTTAT 578422

Query 834 TGCTTTGATACTTCAAAGAACTTATTCACTACTACTAGTTAAGAATATATATTTTCTCTT 893

|| ||||| |||||||||| ||||||||| |||||||||||| ||||||||||||||| |

Sbjct 578423 TGTTTTGAGACTTCAAAGAGCTTATTCACCACTACTAGTTAATAATATATATTTTCTCCT 578482

Query 894 AAGTTTATTATAAATGGGAATTTTCCACATTTAGATGTATAAAAAAAAAAAAAAAAATTG 953

||||||||||||||||||||||||| || |||| |||||| |||||||||||||||

Sbjct 578483 AAGTTTATTATAAATGGGAATTTTCGACTTTTACATGTAT-----AAAAAAAAAAAATTG 578537

Query 954 GAGAAAAAAAGGGGGTATAATATGTAATAAAATAAGTAGATGTGTACACATAATACATAT 1013

|||||||||||||| ||||||||| |||||||||||||||| |||||||||| || ||||

Sbjct 578538 GAGAAAAAAAGGGGATATAATATGCAATAAAATAAGTAGATATGTACACATACTATATAT 578597

Query 1014 TTATATATGTGTGTACGTATATGGGAAAGCTATTTTAAAGAAAATAAATTATAAAATGTT 1073

||||||||||||||||||||||||||||| ||| |||||||||||| |||||||||||||

Sbjct 578598 TTATATATGTGTGTACGTATATGGGAAAGTTATATTAAAGAAAATATATTATAAAATGTT 578657

Query 1074 TATGCAAGAT------------AATATAAATAAATGGGCATATAATTTCATAT------- 1114

|||||||||| ||||||||||||||| ||||| ||||||||

Sbjct 578658 TATGCAAGATAATATAAATATAAATATAAATAAATGGATATATATTTTCATATGACAGTA 578717

Query 1115 ------AATATATATATATATATTCATTTTTTTATGTGAAAAAGG--------------- 1153

|||||||||||||||||||||||| |||| ||||||||

Sbjct 578718 TATATATATATATATATATATATTCATTTTTATATGCGAAAAAGGGGTTTGTGCATAAGG 578777

Query 1154 -TTTTAGTACATATAGATATTTGCAAATATATGGGCGAATATATACATTCAGTGTTAAGA 1212

|| ||||| |||||||||||||||||||||||| | || ||||| || ||||||||||

Sbjct 578778 TTTATAGTATATATAGATATTTGCAAATATATGGACAAACATATATATGCAGTGTTAAGG 578837

Query 1213 GTATAAGTTATTTGGATATGCAATTTGATGGTACATACTATTGTTATATGTATTTCACAC 1272

| | |||||||||||||||| |||||| ||||| ||| || | |||||||||||||| ||

Sbjct 578838 GCACAAGTTATTTGGATATGTAATTTGGTGGTATATAGTAGTATTATATGTATTTCATAC 578897

Query 1273 ATATAGATGCATGTACATATGAATAGGAAGTTTGATTAAATTCAGAATAGATATAAGAAT 1332

||||||||||||||||||||||||||||||||||||| ||||||||||| ||||||||||

Sbjct 578898 ATATAGATGCATGTACATATGAATAGGAAGTTTGATTTAATTCAGAATAAATATAAGAAT 578957

Query 1333 TAATTATCCAAATGAAAAAAAAATG 1357

||||||||||||||| ||||| |||

Sbjct 578958 TAATTATCCAAATGATAAAAA-ATG 578982

> [PCHAS_04_v3](https://plasmodb.org/plasmo/app/record/genomic-sequence/PCHAS_04_v3) | organism=Plasmodium_chabaudi_chabaudi | version=2015-06-18

| length=805816 | SO=chromosome

Length=805816

Score = 839 bits (930), Expect = 0.0

Identities = 1021/1396 (73%), Gaps = 168/1396 (12%)

[**Link to Genome Browser**](https://plasmodb.org/plasmo/app/jbrowse?data=/plasmo/service/jbrowse/bySequenceId/PCHAS_04_v3/&loc=PCHAS_04_v3:608303-609589&tracks=gene), Strand = Plus/Plus

Query 2 CTTGGTGTTTAATGTATTTATAT-GTGCATGTTCGCACTTTCTTTTCTATTAATTTTACT 60

||| |||| ||||||||||||| ||||||| | |||| | ||||||||| ||||||||

Sbjct 608303 CTTAGTGTGTAATGTATTTATAAAGTGCATGATTGCACCTATTTTTCTATTTATTTTACT 608362

Query 61 GAGATTT-ATAATTTCTTGTCAATAAAATATAAAAACATATATACACATGATATATTTTT 119

| ||| || |||||||| |||||||| |||| ||||||||| | |||| ||||||

Sbjct 608363 ATGGTTTTATGTTTTCTTGTTAATAAAATGTAAATACATATATATATGTGATGAATTTTT 608422

Query 120 CCTTTTTATAAATCAAAAAAAACTGTCAAATAAATAATTATAATGTATATATATGTTTTA 179

| || | |||||| | ||||||||||||||||| ||||||||| |||||||||| |||

Sbjct 608423 CATTAT-ATAAAT-ACTAAAAACTGTCAAATAAACAATTATAAT--ATATATATGTCTTA 608478

Query 180 TTTATACAAAATTATGTACATATGTATATTTACATTTAAAACCGTATGAATTTTTATAAA 239

||||| ||||||||||||||||| ||||| |||||||||| || |||||||||||| |

Sbjct 608479 TTTATGCAAAATTATGTACATATATATAT--ACATTTAAAATCGAGTGAATTTTTATATA 608536

Query 240 A-CCCTCATGAAATAAGCTTTTAAATTTAATATAATAAATGAACTTATGAGTTTAAATAA 298

| |||| || |||||| |||||||||||||| ||||||| ||| |||| ||| ||||||

Sbjct 608537 AGCCCTTATAAAATAATCTTTTAAATTTAATGTAATAAAGGAAATTATCCGTT-AAATAA 608595

Query 299 AATGGTAATAATTTTTTAAGTAGTACCTTTTAAGTAGTACCTTTTAAATAGTACCTTTTA 358

||||| ||| || ||| |||||||| |||||| |

Sbjct 608596 AATGGGAAT----------------CCATTT------------TTAAATAGGACCTTTAA 608627

Query 359 AATTATTTCTGCATCTAATATGAATAATATTAAAGCTAATAAAAAATAGGAAAATTTTTT 418

||| |||||||||||| |||||||||||||||| | || || ||| ||||||||||

Sbjct 608628 AATCGTTTCTGCATCTAGTATGAATAATATTAAACATTATGAAG--TAGAAAAATTTTTT 608685

Query 419 ----------ATATATATAAAATGGAGACGTGAAATAAAATTATTATGTTCATATAAAAA 468

||||| ||||| ||||||||||||||||||| ||| |||||||||||

Sbjct 608686 TTCTCTTTATATATACATAAAGTGGAGACGTGAAATAAAATATGCATGGTCATATAAAAA 608745

Query 469 CTGTGATTCGAAAATGTGGGGATAAACACATTTAAAATAGGTACATATATATTTTATTCC 528

||||| |||||||| | || |||| || ||||||||||||| |||||||||||||||

Sbjct 608746 CTGTGGTTCGAAAAATTAGGTATAAGCATGTTTAAAATAGGTATATATATATTTTATTCG 608805

Query 529 TTTTGGGCATTTATTTTTTGTGTTAGCATATAATTATAAA--ATTTTGTTTTTTTCAAAG 586

|||||| ||| ||||||| | | | ||||| ||| ||||||

Sbjct 608806 TTTTGGC----------------TAGTATATAATAAAATAGCATTTTTTTTCCTTCAAAA 608849

Query 587 TTTGGAAATAAAATGTTGAGATGTGTTTAGAAGCAATGCTAAGTTCCAACGTGCTACATG 646

|| | ||||| ||| |||||

Sbjct 608850 TTGAGTTATAAA---------------------------TAA-----AACGT-------- 608869

Query 647 ATGAAATGTTTTAGAAGCAATAATAGGTTCCAACGTGCTGCATGATGAAATAAGGTATGG 706

|||||| ||||||||||||||||| || || ||||| | ||| ||| |||||||

Sbjct 608870 -TGAAATATTTTAGAAGCAATAATAAATTTTAAAGTGCTAACTAGTGAGATAGGGTATGG 608928

Query 707 GTTTGTCTTAAGACACAGTTAGAAGCAGTTTGAATCGTTTCTAAATAATTAAGAA----- 761

|||||||||||||||| ||||||||||||| ||| ||||||||||| ||| ||

Sbjct 608929 GTTTGTCTTAAGACACGGTTAGAAGCAGTTCCAATAGTTTCTAAATAGGTAAAAATAAAT 608988

Query 762 --------------------ATGAA--TGAG--AATATGCG-ATTTATTTACATTTTTGC 796

| ||| | || |||||||| | |||||| |||||| ||

Sbjct 608989 AAAAATAAATAAAAATAAACAAGAAATTAAGGCAATATGCGTACTTATTTGCATTTTCGC 609048

Query 797 GTATTCACAATCTTATTTATGCACATTTAAATTTTATTGCTTTGATACTTCAAAGAACTT 856

|||| || ||| |||||| ||||||||||||||||| |||| | |||||||||| ||

Sbjct 609049 GTATACATAATATTATTTGCGCACATTTAAATTTTATCTCTTTAAGACTTCAAAGAGGTT 609108

Query 857 ATTCACTACTACTAGTTAAGAATATATATTTTCTCTTAAGTTTATTATAAATGGGAATTT 916

| ||||||||||||| ||| |||| |||||||||| |||||||||||||||||| || ||

Sbjct 609109 ACTCACTACTACTAGCTAAAAATAAATATTTTCTCCTAAGTTTATTATAAATGGAAAATT 609168

Query 917 TCCACATTTA-GATGTATAAAAAAAAAAAAAAAAATTGGAGAAAAAAAGGGGGTATAATA 975

| |||||||| |||||| ||||||||||||||||| | || ||| ||||||

Sbjct 609169 TTCACATTTACTATGTAT-------AAAAAAAAAATTGGAGAGAGAACGGGCAAATAATA 609221

Query 976 TGTAATAAAATAAGTAGATGTGTACACATAA-----TACATATTTATATATGTGTGTACG 1030

|| |||| ||||||| || || || ||||| |||||||||||||||||||||||

Sbjct 609222 TGCAATAGGATAAGTAAATATGCACGCATAAATATGTACATATTTATATATGTGTGTACT 609281

Query 1031 TATATGGGAAAGCTATTTTAAAGAAAATAAATTATAAAATGTTTATGCAAGATAATATAA 1090

||||||| |||| |||||||||| |||| |||||||||||||||||||||||| |||||

Sbjct 609282 TATATGGAAAAGACATTTTAAAGAGAATATATTATAAAATGTTTATGCAAGATAGTATAA 609341

Query 1091 A------TAAATGGGCATATAATTTCATATAATATATATATATATATTCATTTTTTTATG 1144

| ||||||| ||||| |||||||| ||| ||| |||||||||||||||| ||||

Sbjct 609342 ATATATGTAAATGGATATATATTTTCATATGATA-ATA-ATATATATTCATTTTTATATG 609399

Query 1145 TGAAAAAGGTTTTAGTACATATAGATATTTGCAAATATA--TGGGCGAATATATACATTC 1202

| |||||||| | | | ||| | | || | ||||| | ||| ||||| || |

Sbjct 609400 TAAAAAAGGTACT--TGCGTATGAGTTTATGTATATATAGGTATTTGAAAATATATATGC 609457

Query 1203 AGTGTTAAGAGTATAAGTTATTTGGATATGCAATTTGATGGTACATACTATTGTTATATG 1262

||||||| |||||| |||| | | || || |||| ||||| |||||| | |||| ||

Sbjct 609458 AGTGTTA--AGTATATATTATGTAGGTACCCATTTTGGTGGTATATACTAGTATTATTTG 609515

Query 1263 TATTTCACACATATAGATGCATGTACATATGAATAGGAAGTTTGATTAAATTCAGAATAG 1322

|||| | ||||| ||| | | |||||||| || || ||| ||| ||||| | |

Sbjct 609516 TATT--GTATATATAAATGTACGGACATATGAGTAAGAGTTTTTATTTAATTCTAAGAAA 609573

Query 1323 ATATAAGAATTAATTA 1338

| ||| ||||||||||

Sbjct 609574 ACATACGAATTAATTA 609589

**Promoter:** *pylap4*

**Gene ID:** PY17X_1323300 (“Query”), PBANKA_1319500, PCHAS_1322800 (all genes are syntenic)

> [PbANKA_13_v3](https://plasmodb.org/plasmo/app/record/genomic-sequence/PbANKA_13_v3) | organism=Plasmodium_berghei_ANKA | version=2017-01-09

| length=2521873 | SO=chromosome

Length=2521873

Score = 2113 bits (2342), Expect = 0.0

Identities = 1353/1467 (92%), Gaps = 48/1467 (3%)

[**Link to Genome Browser**](https://plasmodb.org/plasmo/app/jbrowse?data=/plasmo/service/jbrowse/bySequenceId/PbANKA_13_v3/&loc=PbANKA_13_v3:805036-806460&tracks=gene), Strand = Plus/Plus

Query 2 TAATCCCAAACTTTGATATGTATTTTATCTCTCTATTACTTTATTAAGCTATTTATATAA 61

|||| |||||||||| |||| |||||||||||||||||||||||||| || |||||||||

Sbjct 805036 TAATTCCAAACTTTGGTATGCATTTTATCTCTCTATTACTTTATTAATCTTTTTATATAA 805095

Query 62 TCCAAGTATATTTTTAATATAAATACGGGGATTATCAATATATAAACACATATTCAAAAT 121

| ||||||||||||||||||||||||||||||||||||||||||| ||||||||||||||

Sbjct 805096 TGCAAGTATATTTTTAATATAAATACGGGGATTATCAATATATAACCACATATTCAAAAT 805155

Query 122 GTGTTATTAATTAATTTTTTAATATTTTATAATTAAAAATGAATTAAATAAATACATATG 181

|||||||||||||||||||||||||||| |||||||||||||||||||||||||||||||

Sbjct 805156 GTGTTATTAATTAATTTTTTAATATTTTGTAATTAAAAATGAATTAAATAAATACATATG 805215

Query 182 TTCAGAAAATAATCGAAAGACACTAGTTTTAATCAATTAATTTAGCATTTAATTAATAAA 241

|| |||||||||| || |||||||||||||||| ||||||||||||||||||||||||||

Sbjct 805216 TTAAGAAAATAATGGATAGACACTAGTTTTAATGAATTAATTTAGCATTTAATTAATAAA 805275

Query 242 GGGATTTTTATTTAATGTTAGATCATTCTATATATAGACATATTGGATTTATTCCAATTA 301

|||||||||||||||||||||||||||| ||||||| ||||||| ||||||||||||||

Sbjct 805276 GGGATTTTTATTTAATGTTAGATCATTC--TATATAGGCATATTGAATTTATTCCAATTA 805333

Query 302 GGGTATTTAAACTTCAAGGTATTTTTAATCATAATACCCGTTTGATGTATATATTTTAAA 361

||||||||||||||| ||||||||||||||||||||||| |||| | |||||||||||||

Sbjct 805334 GGGTATTTAAACTTCGAGGTATTTTTAATCATAATACCCATTTGGTATATATATTTTAAA 805393

Query 362 TATGTCATACTATCATAATAATTGCTATAATATCTTATATATTATACTGAATATGTAGAC 421

|| | || ||||||| |||||||||||||||||||||||||||||||||||||| |||||

Sbjct 805394 TACGCCACACTATCAAAATAATTGCTATAATATCTTATATATTATACTGAATATATAGAC 805453

Query 422 CTTATGTATGCAACTCATGCATTCTACAATTTTTTTGAAAAATACGAATGTAGACATTTA 481

|||| |||||||||||||||||||| ||||||||||||| ||||||||||||||||||

Sbjct 805454 CTTA--TATGCAACTCATGCATTCTA-TATTTTTTTGAAAATTACGAATGTAGACATTTA 805510

Query 482 AAGACGTGTTTAATTCAATATAAAAATACGATAAAAAATTGAGAATAATTATAAAATTGT 541

||||| ||||||||||||||||||||||||||||||||||||||||||||||||||||||

Sbjct 805511 AAGACATGTTTAATTCAATATAAAAATACGATAAAAAATTGAGAATAATTATAAAATTGT 805570

Query 542 ATTAAATTAATTTGATTTTTCTTTTTTAATAATATTTTTTTATAAATATACAACTCTAAT 601

||||||||||||||||||| ||||||||||||||||||||||||||||||||||||||||

Sbjct 805571 ATTAAATTAATTTGATTTTCCTTTTTTAATAATATTTTTTTATAAATATACAACTCTAAT 805630

Query 602 AAGATAGCTATGTTTTTTTTTTGTCTTTAATATGTAAAATTATTGTAATAAATATACAAG 661

|||||||||||| ||||||||||||||||||||||||||||||||||||||||||||||

Sbjct 805631 AAGATAGCTATG--TTTTTTTTGTCTTTAATATGTAAAATTATTGTAATAAATATACAAG 805688

Query 662 GTTAAACAATTAATATTTAACAATATTTATACACATATATTCGTGTA--TGTATATCTAC 719

|||||||||||| ||||||||||||||||||||||||||||||||| |||||||||||

Sbjct 805689 GTTAAACAATTAGCATTTAACAATATTTATACACATATATTCGTGTATTTGTATATCTAC 805748

Query 720 AGTTGAGTATAATACACTTCCACACGCACACATGAAATTAAGATAATTTTATTTTTCTGG 779

| ||||||||||||||||| ||||| |||||||||||||||||||||||| |||||||||

Sbjct 805749 ATTTGAGTATAATACACTTGCACACACACACATGAAATTAAGATAATTTTTTTTTTCTGG 805808

Query 780 CTAGCCATCTACAAATGTATATATAAAAATATAATAAAAAATAGCAAAAGAAATAAAATA 839

|||| ||||||||||||||||||||||||||||||||||||||||||| |||||||||||

Sbjct 805809 CTAGTCATCTACAAATGTATATATAAAAATATAATAAAAAATAGCAAAGGAAATAAAATA 805868

Query 840 AAAGAATAATGGCAATAATAAAAATACTAAATTGTGTGACAATGAAAAATATACTCTATA 899

|||||||||| |||||||||||||| |||||| | ||||||||||||||||||| ||||

Sbjct 805869 AAAGAATAATCTCAATAATAAAAATATTAAATTTTATGACAATGAAAAATATACTTTATA 805928

Query 900 ATATATAAAAATACATGTTATTATAATTAGACAGTTAAAAAAATGTGTGTAACAATAAGT 959

|||||||||||| |||||| |||||||||||||||| ||||| |||||||||||||| |

Sbjct 805929 ATATATAAAAATCCATGTT-TTATAATTAGACAGTTTAAAAATAGTGTGTAACAATAAAT 805987

Query 960 TGATGATACATATGTATATACATATATTCTCAATTTTTATGTGTTTATTTTTTATGCAAA 1019

||| |||||||||||||||||||||||||| ||||||||||| |||||||||||||||||

Sbjct 805988 TGAAGATACATATGTATATACATATATTCTTAATTTTTATGTTTTTATTTTTTATGCAAA 806047

Query 1020 CGGAATTTTTAATCGTTCTTTATATATTATATATAATTTAATTTAATTTATTTTATTTTA 1079

|||| |||||||||||||||||||||| ||| |||||||||

Sbjct 806048 TGGAA-TTTTAATCGTTCTTTATATATT-TATTTAATTTAAT------------------ 806087

Query 1080 TTATTATTATTATATTAATTTTATTTTTTGTTACCATTTTATGAATATATTACTTACAAC 1139

||||||||||||||||||| ||| |||||||||| |||||||| || ||||| |||||||

Sbjct 806088 TTATTATTATTATATTAATATTA-TTTTTGTTACAATTTTATGGATGTATTATTTACAAC 806146

Query 1140 GT---TTGTTGTTTCAATGTGTACATTTTAGTAGAATATGTGTATACTTTTATAATATTT 1196

|| |||||||||||||||||||| ||||||||| ||||||||||||||||||||||||

Sbjct 806147 GTTTGTTGTTGTTTCAATGTGTACAATTTAGTAGAGTATGTGTATACTTTTATAATATTT 806206

Query 1197 AAATTATATTATTATTGATATTAATTTTCCAATTGTTATATAAATATTTTTGTGTAT-AA 1255

||||||||||||||||||||||||||||||||||||||| ||||||||||||| ||| ||

Sbjct 806207 AAATTATATTATTATTGATATTAATTTTCCAATTGTTATGTAAATATTTTTGTATATAAA 806266

Query 1256 AATACCTATTTGTCTTAAACTAACAAACAAATATATATATACATATATATATAATTTTTT 1315

||||||||||||||||||||||| |||||| ||||||||||||||||||

Sbjct 806267 AATACCTATTTGTCTTAAACTAATAAACAA------------ATATATATATAATTTTTT 806314

Query 1316 TCATTTTTTGTATGCGTATATGCAATAAAGTAATTTAATTATTACACTAATTATTATAGT 1375

||||||||| |||||||||||||||||||||||||||||||||||| ||||||||||||

Sbjct 806315 TCATTTTTT-TATGCGTATATGCAATAAAGTAATTTAATTATTACATTAATTATTATAGC 806373

Query 1376 TCTCACTTATGCTACAGTCATTTCTTCACTGAAGCAATATTATTTTATATTTATGTATTA 1435

|||||||||||||||||||||||||||||||||| |||||||||||||| |||||||||

Sbjct 806374 TCTCACTTATGCTACAGTCATTTCTTCACTGAAGAAATATTATTTTATACTTATGTATTC 806433

Query 1436 CATTTTTTATTAATAAAAAAAATTATG 1462

|||||||||||||||||||||||||||

Sbjct 806434 CATTTTTTATTAATAAAAAAAATTATG 806460

> [PCHAS_13_v3](https://plasmodb.org/plasmo/app/record/genomic-sequence/PCHAS_13_v3) | organism=Plasmodium_chabaudi_chabaudi | version=2015-06-18

| length=2626858 | SO=chromosome

Length=2626858

Score = 1539 bits (1706), Expect = 0.0

Identities = 1121/1289 (87%), Gaps = 53/1289 (4%)

[**Link to Genome Browser**](https://plasmodb.org/plasmo/app/jbrowse?data=/plasmo/service/jbrowse/bySequenceId/PCHAS_13_v3/&loc=PCHAS_13_v3:879477-880720&tracks=gene), Strand = Plus/Plus

Query 182 TTCAGAAAATAATCGAAAGACACTAGTTTTAATCAATTAATTTAGCATTTAATTAATAAA 241

||||||||||||| |||||||||| |||| ||| |||| |||||||||||||||||||||

Sbjct 879477 TTCAGAAAATAATGGAAAGACACTTGTTTGAATGAATTTATTTAGCATTTAATTAATAAA 879536

Query 242 GGGATTTTTATTTAATGTTAGATCATTCTATATATAGACATATTGGATTTATTCCAATTA 301

||| ||| ||||||||| |||||||||||||||| ||||||||| ||||||||||||||

Sbjct 879537 GGG-TTTGTATTTAATGCTAGATCATTCTATATA--GACATATTGAATTTATTCCAATTA 879593

Query 302 GGGTATTTAAACTTCAAGGTATTTTTAATCATAATACCCGTTTGATGTATATATTTTAAA 361

|||| |||||||||| ||| | |||||| ||||||||| ||||| |||||||||||||||

Sbjct 879594 GGGTGTTTAAACTTCGAGGGACTTTTAACCATAATACCTGTTTGGTGTATATATTTTAAA 879653

Query 362 TATGTCATACTATCATAATAATTGCTATAATATCTTATATATTATACTGAATATGTAGAC 421

||||||||||| ||||||||| || |||||||||||||| ||||||||||||||||||||

Sbjct 879654 TATGTCATACTCTCATAATAAATGTTATAATATCTTATACATTATACTGAATATGTAGAC 879713

Query 422 CTTATGTATGCAAC--TCATGCATTCTACAATTTTTTTGAAAAATACGAATGTAGACATT 479

|||| |||||||| |||||||||||| ||||||||||||||||||||||||||||||

Sbjct 879714 TTTATATATGCAACTGTCATGCATTCTA-TATTTTTTTGAAAAATACGAATGTAGACATT 879772

Query 480 TAAAGACGTGTTTAATTCAATATAAAAATACGATAAAAAATTGAGAATAATTATAAAATT 539

| || ||| ||||||||||||||||||||||| |||||||||||| ||||||||||||||

Sbjct 879773 TTAAAACGCGTTTAATTCAATATAAAAATACGTTAAAAAATTGAGGATAATTATAAAATT 879832

Query 540 GTATTAAATTAATTTGATTTTTCTTTTTTAATAATATTTTTTTATAAATATACAACTCTA 599

|||||||||||||||| |||| ||||||||||| || |||||||||||||||||||||||

Sbjct 879833 GTATTAAATTAATTTGCTTTTCCTTTTTTAATAGTA-TTTTTTATAAATATACAACTCTA 879891

Query 600 ATAAGATAGCTATGTTTTTTTTTTGTCTTTAATATGTAAAATTATTGTAATAAATATACA 659

| || ||||||| ||||||||| |||||||| ||||||||||||||||||||||||

Sbjct 879892 ACAATATAGCTA-----TTTTTTTGTTTTTAATATTTAAAATTATTGTAATAAATATACA 879946

Query 660 AGGTTAAACAATTAATATTTAACAATATTTATACACATATATTCGTGTATGTATATCTAC 719

||| |||||||||||||| |||||||||||||||||||||| | ||||||| ||||| |

Sbjct 879947 AGGGTAAACAATTAATATATAACAATATTTATACACATATACTTGTGTATGCATATCAAT 880006

Query 720 AGTTGAGTATAATACACTTCCACACGCACACATGAAATTAAGATAATTTTATTTTTCTGG 779

| |||||||||||||||| ||||| ||||||| |||||| ||| ||||| |||||||||

Sbjct 880007 ATTTGAGTATAATACACTCACACACACACACAT-AAATTATGAT-ATTTTTTTTTTCTGG 880064

Query 780 CTAGCCATCTACAAATGTATATATAAAAATATAATAAAAAATAGCAAAAGAAATAAAATA 839

||||||| ||||||||||| ||||||||||||||||||||||||| || |||||||||||

Sbjct 880065 CTAGCCAACTACAAATGTACATATAAAAATATAATAAAAAATAGCTAACGAAATAAAATA 880124

Query 840 AAAGAATAATGGCAATAATAAAAATACTAAATTGTGTGACAATGAAAAATATACTCTATA 899

|||||||||| |||||||||| ||| |||||| | ||||||||||| ||||||| ||||

Sbjct 880125 AAAGAATAATTGCAATAATAAGGATATTAAATTTTATGACAATGAAATATATACTTTATA 880184

Query 900 ATATATAAAAATACATGTTATTATAATTAGACAGTTAAAAAAATGTGTGTAACAATAAGT 959

||||||| |||||| ||||||||||| ||||| ||| ||||||||||| |

Sbjct 880185 ATATATA----------TTATTACAATTAGACAGTGTAAAAATTGTATGTAACAATAATT 880234

Query 960 TGATGATACATATGTATATACATATATTCTCAATTTT--TATGTGTTTATTTTTTATGCA 1017

||| |||| ||||||||||||||||||||| |||||| |||| || |||||||||||||

Sbjct 880235 TGAGGATAGATATGTATATACATATATTCTTAATTTTTATATGCGTGTATTTTTTATGCA 880294

Query 1018 AACGGAATTTTTAATCGTTCTTTATATATTATATATAATTTAATTTAATTTATTTTATTT 1077

|| | |||||| | |||||| || |||| |||||||||||| |||

Sbjct 880295 AATAGCATTTTTTACTGTTCTTAATTTATT-TATATAATTTAAGTTA------------- 880340

Query 1078 TATTATTATTATTATATTAATTTTATTTTTTGTTACCATTTTATGAATATATTACTTACA 1137

| ||||||||| ||||||||| ||||||||||||||||||||||| | ||||| |||||

Sbjct 880341 TTTTATTATTAATATATTAATATTATTTTTTGTTACCATTTTATGGAAGTATTATTTACA 880400

Query 1138 ACGT---TTGTTGTTTCAATGTGTACATTTTAGTAGAATATGTGTATACTTTTATAATAT 1194

|||| |||||||||||||||||||||||||| || ||| ||||||||||||||||||

Sbjct 880401 ACGTTTGTTGTTGTTTCAATGTGTACATTTTAGCAGGGTATATGTATACTTTTATAATAT 880460

Query 1195 TTAAATTATATTATTATTGATATTAATTTTCCAATTGTTATATAAATATTTTTGTGTAT- 1253

|||||||||||||||| | |||||||||||||||||||||| |||||| ||||||||||

Sbjct 880461 TTAAATTATATTATTACTAATATTAATTTTCCAATTGTTATGTAAATA-TTTTGTGTATA 880519

Query 1254 AAAATACCTATTTGTCTTAAACTAACAAACAAATATATATATACATATATATATAATTTT 1313

|||||||||||||||||||| || | ||| ||| | ||||||| ||| | ||||

Sbjct 880520 AAAATACCTATTTGTCTTAAGCTGATAAATAAACAAATATATATATA-------ATTTTT 880572

Query 1314 TTTCATTTTTTGTATGCGTATATGCAATAAAGTAATTTAATTATTACACTAATTATTATA 1373

|||||||||||||||||||||||||||||| || ||||||||||||| |||||||||||

Sbjct 880573 TTTCATTTTTTGTATGCGTATATGCAATAATATATTTTAATTATTACATTAATTATTATA 880632

Query 1374 GTTCTCACTTATGCTACAGTCATTTCTTCACTGAAGCAATATTATTTTATATTTATGTAT 1433

| ||||||||||||||||| ||||||||||||||||| |||||||| ||||| ||| |||

Sbjct 880633 GCTCTCACTTATGCTACAGGCATTTCTTCACTGAAGCGATATTATTCTATATATATATAT 880692

Query 1434 TACATTTTTTATTAATAAAAAAAATTATG 1462

| |||||| |||||| |||||||||||

Sbjct 880693 TTCATTTTCTATTAA-CTAAAAAATTATG 880720

**Promoter:** *pytrap*

**Gene ID:** PY17X_1354800 (“Query”), PBANKA_1349800, PCHAS_1354400 (all genes are syntenic)

> [PbANKA_13_v3](https://plasmodb.org/plasmo/app/record/genomic-sequence/PbANKA_13_v3) | organism=Plasmodium_berghei_ANKA | version=2017-01-09

| length=2521873 | SO=chromosome

Length=2521873

Score = 1817 bits (2014), Expect = 0.0

Identities = 1396/1662 (84%), Gaps = 130/1662 (8%)

[**Link to Genome Browser**](https://plasmodb.org/plasmo/app/jbrowse?data=/plasmo/service/jbrowse/bySequenceId/PbANKA_13_v3/&loc=PbANKA_13_v3:1911005-1912622&tracks=gene), Strand = Plus/Minus

Query 1 GTTAAATAGGTTTATACTTAAAAATTGCTTTTT-AGATTTGCCCCCTTTTTTGTGTTCAA 59

|||||||||||||| ||| ||||||| ||||| | | ||||||||||||||||||||||

Sbjct 1912622 GTTAAATAGGTTTA--CTTGAAAATTGTTTTTTTAAAATTGCCCCCTTTTTTGTGTTCAA 1912565

Query 60 AAAATATACGAATTTTTATTGAATTATATCAACATAAAAATAATGTACATATTATTTAT- 118

||||||||| ||||||||||||||||||| |||||||||||||| || ||||||||||

Sbjct 1912564 AAAATATACAAATTTTTATTGAATTATATGAACATAAAAATAATTTATATATTATTTAAA 1912505

Query 119 ------------------TCCTACATATAT---------ATATATTGAGAGAAAATATAG 151

|| ||| |||| ||||||||| ||||||| | |

Sbjct 1912504 TATGGGTAGGGGAAATTGTCTTACCCATATTATTCCTACATATATTGAAAGAAAATGTGG 1912445

Query 152 TGGATGTTATTTATGAGTATATATGTAT----ATGTATACGATTTTTCTCTTTTCTCCTT 207

||||| |||||||||||||||||||||| ||| |||| |||||| | |||||||

Sbjct 1912444 TGGATATTATTTATGAGTATATATGTATGTATATGCATACAATTTTTATTTTTTCTC--- 1912388

Query 208 TCCCTTTTCTCTTTTTTTGCTCATTCGCCAAATATTTACACCACTCTTCCTTATTGTAAC 267

||||||| |||||| |||||| ||||| ||||||||||| |||||||

Sbjct 1912387 -----------TTTTTTTACTCATTAACCAAATTTTTACGTCACTCTTCCTTGTTGTAAC 1912339

Query 268 ATTTATACAAGGACACCTTCAAAAAAAATAAATATATGCAAAATATAAATTGAGATAAT- 326

|||||||||||| ||||||||||||| || |||||| |||||||||||||||||||||

Sbjct 1912338 ATTTATACAAGGTAACCTTCAAAAAAA-TATATATATACAAAATATAAATTGAGATAATA 1912280

Query 327 -------GAATAAACCGCCAAAAAAACAAGACACAAAAAATAAGCCTATTTTATACAACA 379

|||||||||| ||| ||| || | |||||| |||||||||||||||||||||

Sbjct 1912279 AATAAATGAATAAACCGAAAAAGAAATAAAATACAAAATATAAGCCTATTTTATACAACA 1912220

Query 380 AATATATGTATATAAA-----------------------------------------AAT 398

|||||||||||||||| |||

Sbjct 1912219 AATATATGTATATAAATATTGTTTATTTATGTAATAATAAAATTAAATTGCCATAAAAAT 1912160

Query 399 TGTGTAAATTTTTATTTATTCAATGAAGGGCTATATGTAGTTATTTATAATTAATAAAAT 458

||||||||||| ||||||||||||| ||| ||||||| || |||||||| ||||||||||

Sbjct 1912159 TGTGTAAATTTATATTTATTCAATGGAGGCCTATATGCAGGTATTTATATTTAATAAAAT 1912100

Query 459 TATGCATGC-AATTGAATACATGTAAAAAAGAGAAACTCCTCCATTTTATAATTTATATA 517

||||||||| |||||||||||||||||||||||||| |||| | |||||||||||||| |

Sbjct 1912099 TATGCATGCTAATTGAATACATGTAAAAAAGAGAAATTCCTTCGTTTTATAATTTATAAA 1912040

Query 518 GCTATAAAAAACAAACAAAAAATGGTTAAATGCTAATTTATATATAATATCTCTTTTATT 577

||||||||||| |||||||||||||||||||| |||||||||||||| ||||||||||||

Sbjct 1912039 GCTATAAAAAATAAACAAAAAATGGTTAAATGATAATTTATATATAACATCTCTTTTATT 1911980

Query 578 ATTTTTTTGTAGATTAATTTCTCTTTCCATATTGCACACATATTAACTTTCTACATGTAC 637

||||||||||| ||| |||| | |||||||| | |||| |||||| |||| ||||||| |

Sbjct 1911979 ATTTTTTTGTATATTCATTTATTTTTCCATACTACACATATATTA-CTTTTTACATGTGC 1911921

Query 638 ATATAATAAATTTTTTTGGGGTTGTAATAATTAGCTCGATATTTTAAACCCTTAATTATA 697

|||||||||| ||| |||| ||||||||||||||||||||||||||||| ||||||||

Sbjct 1911920 ATATAATAAAATTTGTTGG--TTGTAATAATTAGCTCGATATTTTAAACCTTTAATTATT 1911863

Query 698 TTATTTTTACATGTAACTCATATTAATATATATAATGTCTTCCACAAAATTGATCAAATA 757

| ||||||||||||| |||||||||||| | |||||||||||||||||||||||| ||||

Sbjct 1911862 TAATTTTTACATGTAGCTCATATTAATACACATAATGTCTTCCACAAAATTGATCTAATA 1911803

Query 758 TATTTCCTAAATATGTTACAATATATATCCATAAAATACATACATGTAATATATTTAATA 817

||||||||||||||||||||| ||||||||||||||| ||||||||| |||||||||||

Sbjct 1911802 TATTTCCTAAATATGTTACAACATATATCCATAAAATCCATACATGTGGTATATTTAATA 1911743

Query 818 ATAATTTTCTGATATAAATATAGACAGCTTTTTTATATATATTAAAACCATTTAAAAAAG 877

||||||| ||||||||||| ||||| ||||||||||||| |||| |||||||||||||

Sbjct 1911742 ATAATTT-CTGATATAAATCCAGACAACTTTTTTATATAT--TAAAGCCATTTAAAAAAG 1911686

Query 878 TAAATTTTATAAATTTTGTTT-AATTTTCTTTATATATATA-ATATATATATACATTTAT 935

||||||||||||||| ||||| |||||||| |||||| ||| ||||||||||| || |||

Sbjct 1911685 TAAATTTTATAAATTATGTTTGAATTTTCTCTATATACATATATATATATATATATATAT 1911626

Query 936 ATATACTCTTGTTCTTTTTATCGATTAAAAAAATATATAATATCCATTATATTTATTTTT 995

|||||| |||| | |||||||||||||||||| |||||| | ||||||||||||||||

Sbjct 1911625 ATATACACTTGCT---TTTATCGATTAAAAAAATTTATAATGTTCATTATATTTATTTTT 1911569

Query 996 TAACAATTAAAAATATATAAAATGTACCCCTTGTGCTTGAAGCAACATTTTTTATATTTA 1055

||||||||||||||||||||||||||| ||||||||||||||| |||||||||||||||

Sbjct 1911568 TAACAATTAAAAATATATAAAATGTACATCTTGTGCTTGAAGCATCATTTTTTATATTTA 1911509

Query 1056 ACTGTTGTATCTTTTTTTACATATATTTGTTCACATTCTTTGGGATGATATTAAATAATA 1115

|||||||||||||||||||||||||||||||||||| | |||| ||||||||||||||||

Sbjct 1911508 ACTGTTGTATCTTTTTTTACATATATTTGTTCACATCCATTGGAATGATATTAAATAATA 1911449

Query 1116 TAATTTTCGAAGAGAAATATTTTTAAATACTTTTTTTAGTGCTTGCATTATTTTTATGAT 1175

|||||||||||||||| |||| |||||| |||||||||||| ||||||| ||||||||||

Sbjct 1911448 TAATTTTCGAAGAGAAGTATTCTTAAATTCTTTTTTTAGTGATTGCATTGTTTTTATGAT 1911389

Query 1176 ATATATTAACATTCATAAAATATATATTTGTTGAGTGTTGGTTGCCAGTTTATTGAATTA 1235

||||||||||| |||||||||||||||||||||||| |||||||||| | || ||

Sbjct 1911388 ATATATTAACACTCATAAAATATATATTTGTTGAGT----GTTGCCAGTTGGATAAACTA 1911333

Query 1236 GCTATATTTTTAAATACTAAATATA-TTTTTTTAAATTGGTTATGATCATATTCTAATCC 1294

||||||||||||||| ||||||||| |||||||||||||||||| || ||| ||| |

Sbjct 1911332 GCTATATTTTTAAATGCTAAATATATTTTTTTTAAATTGGTTATTATAATACCACAATTC 1911273

Query 1295 GTATTATATTGCGTATGTGTATATATATAACGGAAAAAAAGGAAAACATTTAATTTCCTC 1354

|||||||| |||||||||||||||||||||||||||||||||||||||||||||||||||

Sbjct 1911272 GTATTATA-TGCGTATGTGTATATATATAACGGAAAAAAAGGAAAACATTTAATTTCCTC 1911214

Query 1355 AGACGCTATTGAATTAAATTAACTATATATCAGTTTTATATAAGAAAAGGTAACACACTC 1414

||||| ||||||| |||||||||||||||| | |||| |||||||||||||||

Sbjct 1911213 AGACGTTATTGAAGTAAATTAACTATATATAAATTTTGTATAAGAAAAGGTAA------- 1911161

Query 1415 TCTCTCTATATATATATAATTGCAAACGTGTAGACATTTTTATATATGGCCAAATAGTAA 1474

||||||||| ||||||||||||| ||||||| ||||||||||||||||||||||

Sbjct 1911160 -----CTATATATACATAATTGCAAACGCATAGACATGTTTATATATGGCCAAATAGTAA 1911106

Query 1475 ATACAAAATAATTCCTCACTTTTATTCTCTTACATATATTATAATACATACATAGACACA 1534

||| ||||||| | ||||| ||||| || ||||||||||||||||||||||||||||||

Sbjct 1911105 ATATAAAATAACTTTTCACTATTATTTTCATACATATATTATAATACATACATAGACACA 1911046

Query 1535 TAATTTTACCCATTCCCCATTTCTCTTATAGACAGAAACATG 1576

||| ||| |||||| || |||| |||||| | ||||| |||

Sbjct 1911045 TAA-TTTGCCCATTTTCCCTTTCCCTTATAAAAAGAAATATG 1911005

> [PCHAS_13_v3](https://plasmodb.org/plasmo/app/record/genomic-sequence/PCHAS_13_v3) | organism=Plasmodium_chabaudi_chabaudi | version=2015-06-18

| length=2626858 | SO=chromosome

Length=2626858

Score = 1200 bits (1330), Expect = 0.0

Identities = 1003/1211 (83%), Gaps = 78/1211 (6%)

[**Link to Genome Browser**](https://plasmodb.org/plasmo/app/jbrowse?data=/plasmo/service/jbrowse/bySequenceId/PCHAS_13_v3/&loc=PCHAS_13_v3:1990207-1991364&tracks=gene), Strand = Plus/Minus

Query 391 ATAAAAATTGTGTAAATTTTTATTTATTCAATGAAGGGCTATATGTAGTTATTTATAATT 450

||||||||||||| |||||||||||||||||||||||||||||| || ||||||||||

Sbjct 1991364 ATAAAAATTGTGTTAATTTTTATTTATTCAATGAAGGGCTATATACAGACATTTATAATT 1991305

Query 451 AATAAAATTATGCATGC-AATTGAATACATGTAAAAAAGAGAAACTCCTCCATTTTATAA 509

||||||||||||||||| |||| ||||||||||||||||||||| ||| | |||| |||

Sbjct 1991304 AATAAAATTATGCATGCTAATTAAATACATGTAAAAAAGAGAAAATCCCTCGTTTTGTAA 1991245

Query 510 TTTATATAGCTATAAAAAACAAACAA-----------AAAATGGTTAAATGCTAATTTAT 558

|||||| |||||||||||| ||| || |||||||||||| | ||||||||

Sbjct 1991244 TTTATAAAGCTATAAAAAATAAATAATAAATAAATTAAAAATGGTTAAAGGATAATTTAT 1991185

Query 559 ATATAATATCTCTTTTATTATTTTTTTGTAGATTAATTTCTCTTTCCATATTGCACACAT 618

|||||| | ||| ||||||||||||||| ||| | ||||||||||| | | |||||||

Sbjct 1991184 ATATAACAATTCTCTTATTATTTTTTTGTTTATTCAATTCTCTTTCCACACTACACACAT 1991125

Query 619 ATTAACTTTCTACATGTACATATAATAAATTTTTTTGGGGTTGTAATAATTAGCTCGATA 678

|||| |||||||||||||||||| |||| |||||| |||||||||| | |||||| |

Sbjct 1991124 ATTA-CTTTCTACATGTACATATCATAATTTTTTT--GGGTTGTAATGAGCAGCTCGCCA 1991068

Query 679 TTTTAAACCCTTAATTATATTATTTTTACATGTAACTCATATTAATATA--TATAATGTC 736

||| || || |||| |||||||||||||||||| ||||| |||||| | |||||||||

Sbjct 1991067 TTT-AAGCCTTTAAAAATATTATTTTTACATGTAGCTCATGTTAATACACATATAATGTC 1991009

Query 737 TTCCACAAAATTGATCAAATATATTTCCTAAATATGTTACAATATATATCCATAAAATAC 796

|| | ||||||||||||||||||| ||||||| ||| ||| |||| |||||||||

Sbjct 1991008 TTTTATGAAATTGATCAAATATATTTACTAAATACTTTATAATTGATATGGATAAAATAC 1990949

Query 797 ATACATGTAATATATTTAATAATAATTTTCTGATATAAATATAGACAGCTTTTTTATATA 856

|||||||| |||||||||||||| || |||| |||| ||||||||||||||||||

Sbjct 1990948 ATACATGTTATATATTTAATAATTCTT----GATACAAATTCAGACAGCTTTTTTATATA 1990893

Query 857 TATTAAAACCATTTAAAAAAGTAAATTTTATAAATTTTGTTT-AATTTTCTTTATATATA 915

| || ||||||||||||| |||| ||||||||||| | ||| |||||||| | |||| |

Sbjct 1990892 T--TACAACCATTTAAAAA-GTAA-TTTTATAAATTATTTTTGAATTTTCTCTCTATACA 1990837

Query 916 TAATATATATATACATTTATATATACTCTTGTTCTTTTTATCGATTAAAAAAATATATAA 975

|| ||||||||||||||| ||||||| ||||| |||||||||||||||||| |||||||

Sbjct 1990836 TA-TATATATATACATTTGTATATACGTTTGTTGTTTTTATCGATTAAAAAATTATATAA 1990778

Query 976 TATCCATTATATTTATTTTTTAACAATTAAAAATATATAAAATGTACCCCTTGTGCTTGA 1035

| | ||||||||||||||||||||||| ||||||||||||||||||| |||||||||||

Sbjct 1990777 TGCCAATTATATTTATTTTTTAACAATTCAAAATATATAAAATGTACCACTTGTGCTTGA 1990718

Query 1036 AGCAACATTTTTTATATTTAACTGTTGTATCTTTTTTTACATATATTTGTTCACATTCTT 1095

|| || |||||| |||||||||||| |||||||||||||||| |||||| | || |||

Sbjct 1990717 AGTCTCAATTTTTACATTTAACTGTTGGATCTTTTTTTACATATTTTTGTTTATATCCTT 1990658

Query 1096 TGGGATGATATTAAATAATATAATTTTCGAAGAGAAATATTTTTAAATACTTTTTTTAGT 1155

||||||||||||||||||||||||||||||||||||||||||||||||| ||||||||||

Sbjct 1990657 TGGGATGATATTAAATAATATAATTTTCGAAGAGAAATATTTTTAAATATTTTTTTTAGT 1990598

Query 1156 GCTTGCATTATTTTTATGATATATATTAACATTCATAAAATATATATTTGTTGAGTGTTG 1215

|||||||| |||||||||||||||||||| |||||||||| ||||||| ||||||

Sbjct 1990597 CCTTGCATTGTTTTTATGATATATATTAACGTTCATAAAATTTATATTTTTTGAGT---- 1990542

Query 1216 GTTGCCAGTTTATTGAATTAGCTATATTTTTAAATACTAAATATATTTTTTTAAATTGGT 1275

| ||| | || | ||| ||||||||| |||||||||| ||||||||||||||||||||||

Sbjct 1990541 GCTGCTAATTGAATGAGTTAGCTATACTTTTAAATACGAAATATATTTTTTTAAATTGGT 1990482

Query 1276 TATGATCATATTCTAATCCGTATTATATTGCGTATGTGTATATATATAACGGAAAAAAAG 1335

||| || | ||||||||||||| |||| ||||||||||||||||||| |||||||

Sbjct 1990481 TAT--TC----GCCAATCCGTATTATA-TGCGCATGTGTATATATATAACGG-AAAAAAG 1990430

Query 1336 GAAAACATTTAATTTCCTCAGACGCTATTGAATTAAATTAACTATATATCAG-TTTTATA 1394

||||| ||||||||||||||||||||||| || ||||||||||||||||| |||| ||

Sbjct 1990429 GAAAATATTTAATTTCCTCAGACGCTATTAAAGCCAATTAACTATATATCAGTTTTTGTA 1990370

Query 1395 TAAG-AAAAGGTAAC-------ACAC-TCTCTCTCTATATATATATAATTGCAAACGTGT 1445

|||| |||||||||| | || | | |||||||||| ||||||||||||| |

Sbjct 1990369 TAAGAAAAAGGTAACTCTATATATACAACCATATCTATATATACATAATTGCAAACGCAT 1990310

Query 1446 AGACATTTTTATATATGGCCAAATAGTAAATACAAAATAATTCCTCACTTTTATTCTCTT 1505

| |||||||||||||||| ||||||| |||||| | ||| ||||| || |||

Sbjct 1990309 ACACATTTTTATATATGG-------GTAAATAGAAAATACCTTCTCTCTTTTCTT-TCT- 1990259

Query 1506 ACATATATTATAATACATACATAGACACATAATTTTACCCATTCCCCATTTCTCTTATAG 1565

| ||||||||||||||||||||| || ||| |||| |||||

Sbjct 1990258 -------------TGCATACATAGACACATAATTTT-CCTGTTC-----TTCTATTATAA 1990218

Query 1566 ACAGAAACATG 1576

|||||| |||

Sbjct 1990217 GCAGAAATATG 1990207

**Promoter:** *pyuis4*

**Gene ID:** PY17X_0502200 (“Query”), PBANKA_0501200, PCHAS_0501300 (all genes are syntenic)

> [PbANKA_05_v3](https://plasmodb.org/plasmo/app/record/genomic-sequence/PbANKA_05_v3) | organism=Plasmodium_berghei_ANKA | version=2017-01-09

| length=931174 | SO=chromosome

Length=931174

Score = 2012 bits (2230), Expect = 0.0

Identities = 1301/1423 (91%), Gaps = 50/1423 (4%)

[**Link to Genome Browser**](https://plasmodb.org/plasmo/app/jbrowse?data=/plasmo/service/jbrowse/bySequenceId/PbANKA_05_v3/&loc=PbANKA_05_v3:50472-51865&tracks=gene), Strand = Plus/Minus

Query 10 CTTCTTTGAGCAAATACTGAACAATAGGGAATGCTTCTATGTAACTGTGGATATATATGT 69

|||||||||||||||||||||||||| |||||||||||||| | | |||||| |||||||

Sbjct 51865 CTTCTTTGAGCAAATACTGAACAATAAGGAATGCTTCTATGCATCCGTGGATGTATATGT 51806

Query 70 ACGTAATAAAATAATTTTTTGTCCATAAAAATATCTTTAACAGCTACTATACAAAAGCAA 129

||||||||||||||||||||||||||||||||||||||||||||| ||||||||||||

Sbjct 51805 ACGTAATAAAATAATTTTTTGTCCATAAAAATATCTTTAACAGCTGTTATACAAAAGCAT 51746

Query 130 TGAATTGGTTAATACATTTTTGTCGTAAATAAAATAAATGAATTAATGGTACAAAATTAC 189

|||||||| | |||||||||||||||||||||||||||||||||||||||||||||||||

Sbjct 51745 TGAATTGGCTGATACATTTTTGTCGTAAATAAAATAAATGAATTAATGGTACAAAATTAC 51686

Query 190 AATAAAAAGGGAGCAAGTTTATTGTATTTTGTTTAATTTATGTCCTTTCCATTTTATTAA 249

|||||||||| ||| | ||||||| |||||||||||||||||||||||||||||||||||

Sbjct 51685 AATAAAAAGGAAGCGAATTTATTGCATTTTGTTTAATTTATGTCCTTTCCATTTTATTAA 51626

Query 250 TACAATTATGATATTCACTAATTCGTATTAAACATATCTTTTGCATGTACCAAAGGTTTG 309

|||||||||| || | | ||||||||||||||||||| |||||||||||||||| |||||

Sbjct 51625 TACAATTATGGTACT-AGTAATTCGTATTAAACATATATTTTGCATGTACCAAATGTTTG 51567

Query 310 GGGAATAAGCATATGAATCCCATTAAATTTAATGATATTCTGCGATTTTTCTTGTATTTA 369

|| |||||||||||| |||||| |||||||||| ||||||||||||||||||| ||||||

Sbjct 51566 GGAAATAAGCATATGGATCCCACTAAATTTAATAATATTCTGCGATTTTTCTTATATTTA 51507

Query 370 CTATTAAATATAATGGATTCATTTTTTGATGCATGCAATTTTTTCTTTTAATGTATTAAT 429

||||||||||||||||||||||||||||||||||||||||||||||||||||||||||||

Sbjct 51506 CTATTAAATATAATGGATTCATTTTTTGATGCATGCAATTTTTTCTTTTAATGTATTAAT 51447

Query 430 TAGTGTAATAATTTTGTAAAAACTTTATTCATTTATTTATTTTCCCTATTATATTGGGTT 489

|||||| | ||||||| |||||||||| ||||||||||||||||| ||||||||||| ||

Sbjct 51446 TAGTGTGACAATTTTG-AAAAACTTTACTCATTTATTTATTTTCCTTATTATATTGGATT 51388

Query 490 CATAATACCATAATTATTATATAAACCACATTAAATAATTTGTAATTTATTCAAGGGTAT 549

||||||||| |||||||||||||||||||||||||||||||||||||||| |||||||

Sbjct 51387 TATAATACCAGTATTATTATATAAACCACATTAAATAATTTGTAATTTATTCGAGGGTAT 51328

Query 550 TAAAAAAAACATATATAAAATGCATATATCCACATATGGTTCATTATAGGATGAATAAAA 609

|||||||||||||| ||||||||||||||||||||||| |||||||||| |||||||||

Sbjct 51327 CAAAAAAAACATATACAAAATGCATATATCCACATATGGCTCATTATAGGGTGAATAAAA 51268

Query 610 ATGGGAACAACATATGAATTATTATATCATGAAAATAATGAAAAAACATAAATTATATGG 669

||||||||| || || |||||||||||||||||| ||||||||||| ||||||||||||

Sbjct 51267 ATGGGAACAGCAGATTAATTATTATATCATGAAAGTAATGAAAAAATTTAAATTATATGG 51208

Query 670 ATATATACATATATAAATATATTAAAAAATAATAATAAATAAATAAAATGTATTATAAAC 729

|||||||||||||| ||||| ||||||||||||||||||||||||||||||||||||||

Sbjct 51207 ATATATACATATAT-AATATGTTAAAAAATAATAATAAATAAATAAAATGTATTATAAAT 51149

Query 730 CTTATAAAATAAGTGATGTTGTGGATAATCCACGAAATATGCCATAAATAGACACTGAAC 789

|||||||||||||| ||||||| ||||||||||||||||||||||||||||||||||||

Sbjct 51148 CTTATAAAATAAGTTTTGTTGTGAATAATCCACGAAATATGCCATAAATAGACACTGAAC 51089

Query 790 AAATTAGTGGTTCTTAATATTTTTTTGGATACATGCGGATATTACCATTGACAGATGATT 849

||||||||||||||||||||| |||||||||||||||||||||| ||||| |||||||||

Sbjct 51088 AAATTAGTGGTTCTTAATATTATTTTGGATACATGCGGATATTATCATTGTCAGATGATT 51029

Query 850 TATTTTTTGTTATTTTTAAATTATACATATTCATAGTTTATATAGTCCTCAAAAAATAGG 909

||||||||||||||||||||||||| ||||||||| || ||||||||||||||||||||

Sbjct 51028 TATTTTTTGTTATTTTTAAATTATAAATATTCATA--TTTTATAGTCCTCAAAAAATAGG 50971

Query 910 ATGTTTTATTCTTTTATAGCTATATTTTATGGTTGATCCTTTCCTTTTATGGTGTTTCAT 969

|||||||||| |||||||||||||||||||||||||||||||||||||||||||||||||

Sbjct 50970 ATGTTTTATTTTTTTATAGCTATATTTTATGGTTGATCCTTTCCTTTTATGGTGTTTCAT 50911

Query 970 AAAAATTTTATTGAGCTATATATAATCCAATAAAAAAAGGTGATTGAATTTTGAAATATA 1029

|||||||||||||||||||||| ||||||||||||||||| ||||||||||||||||||

Sbjct 50910 AAAAATTTTATTGAGCTATATACAATCCAATAAAAAAAGGCAATTGAATTTTGAAATATA 50851

Query 1030 TTAAACTTTTTTTATAATAAAATAAATATAATTATTTTTAAATAATATATATTATATATA 1089

||||||| |||||||||||||||||||||| ||||||||||||| |

Sbjct 50850 TTAAACTATTTTTATAATAAAATAAATATA---ATTTTTAAATAATGT------------ 50806

Query 1090 TATATTATATATTATATATTTTTTTTTATCTTTACACAGAATTTTTTTTATAGAGTCCAA 1149

|||||||||||| | ||||||||||||||||||| ||||||| ||||||||||

Sbjct 50805 TATATTATATATAA--------TTTTTATCTTTACACAGAAATTTTTTTTTAGAGTCCAA 50754

Query 1150 TATATATAATTAGTTATATAT-----------------ATACACCACCATAAATAATTAT 1192

||||||||||||||||||||| ||||||||||||||||||||||

Sbjct 50753 TATATATAATTAGTTATATATATAGAGATATACACTACATACACCACCATAAATAATTAT 50694

Query 1193 AAGGAAAATCAGTTATTTAAATTTTAACTGAAGAAATTAAATAAGTATATAAAAAAAGAA 1252

|||||||| ||||||||| ||||||||||||||||||||||||| ||||| |||||||||

Sbjct 50693 AAGGAAAACCAGTTATTTTAATTTTAACTGAAGAAATTAAATAAATATAT-AAAAAAGAA 50635

Query 1253 AAGAACAAAAAATAAAACGACAACAACCTTAAAAATTTTTTATTATTACATATTTATACA 1312

|||||||||||||||||||||| ||||||||||| ||| |||||||||||||||||||||

Sbjct 50634 AAGAACAAAAAATAAAACGACAGCAACCTTAAAATTTTATTATTATTACATATTTATACA 50575

Query 1313 TAAAAAAAATAAAATACCTAGACAAAAAATAATAAATATTATAGATCGATATT-GACTAC 1371

||||||||||||||||| ||||||||||||||||||||||||||||||||||| | |||

Sbjct 50574 TAAAAAAAATAAAATACTTAGACAAAAAATAATAAATATTATAGATCGATATTAGCATAC 50515

Query 1372 ATATATACCTTTCAGCACATA---ATTACATCTGAATAAAATG 1411

||||||||||||||||||||| ||||| |||||||||||||

Sbjct 50514 ATATATACCTTTCAGCACATAATTATTACGTCTGAATAAAATG 50472

> [PCHAS_05_v3](https://plasmodb.org/plasmo/app/record/genomic-sequence/PCHAS_05_v3) | organism=Plasmodium_chabaudi_chabaudi | version=2015-06-18

| length=950652 | SO=chromosome

Length=950652

Score = 1954 bits (2166), Expect = 0.0

Identities = 1294/1426 (91%), Gaps = 39/1426 (3%)

[**Link to Genome Browser**](https://plasmodb.org/plasmo/app/jbrowse?data=/plasmo/service/jbrowse/bySequenceId/PCHAS_05_v3/&loc=PCHAS_05_v3:58662-60073&tracks=gene), Strand = Plus/Minus

Query 11 TTCTTTGAGCAAATACTGAACAATAGGGAATGCTTCTATGTAACTGTGGATATATATGTA 70

|||| |||||||||||||||||||||| ||||||| ||| ||| ||| | ||||||| |

Sbjct 60073 TTCTATGAGCAAATACTGAACAATAGGAAATGCTTGTATACAACCGTGAACATATATGCA 60014

Query 71 CGTAATAAAATAATTTTTTGTCCATAAAAATATCTTTAACAGCTACTATACAAAAGCAAT 130

|||||||||||||||||||||||||||||||||||||||||||||| ||||||||||| |

Sbjct 60013 CGTAATAAAATAATTTTTTGTCCATAAAAATATCTTTAACAGCTAC-ATACAAAAGCATT 59955

Query 131 GAATTGGTTAATACATTTTTGTCGTAAATAAAATAAATGAATTAATGGTACAAAATTACA 190

|||||| || ||| |||||||| || ||| |||||||||||||| || ||| ||||||||

Sbjct 59954 GAATTGATTGATATATTTTTGTTGTGAATCAAATAAATGAATTAGTGATACCAAATTACA 59895

Query 191 ATAAAAAGGGAGCAAGTTTATTGTATTTTGTTTAATTTATGTCCTTTCCATTTTATTAAT 250

||||||| | || | | |||||| ||| || |||||||||||||||| |||||||||| |

Sbjct 59894 ATAAAAAAGAAGTAGGCTTATTGCATTCTGCTTAATTTATGTCCTTTTCATTTTATTAGT 59835

Query 251 ACAATTATGATATTCACTAATTCGTATTAAACATATCTTTTGCATGTACCAAAGGTTTGG 310

||||||||| || ||||||||||||||||||||||||||||||||||||||||| |||||

Sbjct 59834 ACAATTATGGTACTCACTAATTCGTATTAAACATATCTTTTGCATGTACCAAAGATTTGG 59775

Query 311 GGAATAAGCATATGAATCCCATTAAATTTAATGATATTCTGCGATTTTTCTTGTATTTAC 370

| |||||||||||||||||||||||||||||| ||||| || ||||||| |||||||||

Sbjct 59774 GAAATAAGCATATGAATCCCATTAAATTTAATAATATTTTGA-ATTTTTCATGTATTTAC 59716

Query 371 TATTAAATATAATGGATTCATTTTTTGATGCATGCAATTTTTTCTTTTAATGTATTAATT 430

||||||||||||||||||||||||||||||||||||||||||||||||||||||||||||

Sbjct 59715 TATTAAATATAATGGATTCATTTTTTGATGCATGCAATTTTTTCTTTTAATGTATTAATT 59656

Query 431 AGTGTAATAATTTTGTAAAAACTTTATTCATTTATTTATTTTCCCTATTATATTGGGTTC 490

|||| | |||||||||||||||||| || ||||||||||||||||||||||||| |||

Sbjct 59655 GGTGTGACAATTTTGTAAAAACTTTACTCGCTTATTTATTTTCCCTATTATATTGGTTTC 59596

Query 491 ATAATACCATAATTATTATATAAACCACATTAAATAATTTGTAATTTATTCAAGGGTATT 550

|||||| |||||||||||||||||||||||||||||||||||||||||||| ||||

Sbjct 59595 ATAATATATGCATTATTATATAAACCACATTAAATAATTTGTAATTTATTCAAGGATATT 59536

Query 551 AAAAAAAACATATATAAAATGCATATATCCACATATGGTTCATTATAGGATGAATAAAAA 610

|||||||||||||||| |||||||| ||||||||||||||||||||||| ||||||||||

Sbjct 59535 AAAAAAAACATATATATAATGCATACATCCACATATGGTTCATTATAGGGTGAATAAAAA 59476

Query 611 TGGGAACAACATATGAATTATTATATCATGAAAATAATGAAAAAACATAAATTATATGGA 670

||||||||||| |||||||||||||||||||||||||||||||||| |||||||||||||

Sbjct 59475 TGGGAACAACAGATGAATTATTATATCATGAAAATAATGAAAAAACGTAAATTATATGGA 59416

Query 671 TATATACATATATAAATATATTAAAAAATAATAATAAATAAATAAAATGTATTATAAACC 730

||||||||||||||||||| |||||||||||||||||||||||||||| |||||||||

Sbjct 59415 TATATACATATATAAATATGTTAAAAAATAATAATAAATAAATAAAATTTATTATAAATT 59356

Query 731 TTATAAAATAAGTGATGTTGTGGATAATCCACGAAATATGCCATAAATAGACACTGAACA 790

| |||||||||||| ||||||||||||||||||||||||||||||||||||||||||||

Sbjct 59355 TCATAAAATAAGTGTTGTTGTGGATAATCCACGAAATATGCCATAAATAGACACTGAACG 59296

Query 791 AATTAGTGGTTCTTAATATTTTTTTGGATACATGCGGATATTACCATTGACAGATGATTT 850

|||||||||||||||||||| |||||||||||||| || |||| ||||| ||||||||||

Sbjct 59295 AATTAGTGGTTCTTAATATTATTTTGGATACATGCTGAAATTATCATTGTCAGATGATTT 59236

Query 851 ATTTTTTGTTATTTTTAAATTATACATATTCATAGTTTATATAGTCCTCAAAAAATAGGA 910

|||||||||||||||| |||||||||||||||||||||||||||||||||||| ||||||

Sbjct 59235 ATTTTTTGTTATTTTTTAATTATACATATTCATAGTTTATATAGTCCTCAAAACATAGGA 59176

Query 911 TGTTTTATTCTTTTATAGCTATATTTTATGGTTGATCCTTTCCTTTTATGGTGTTTCATA 970

||||||||||||||||||||||||||||||||||||||||||||||||||||||||||||

Sbjct 59175 TGTTTTATTCTTTTATAGCTATATTTTATGGTTGATCCTTTCCTTTTATGGTGTTTCATA 59116

Query 971 AAAATTTTATTGAGC--TATATATAATCCAATAAAAAAAGGTGATTGAATTTTGAAATAT 1028

|||||||| |||||| |||||||||||||||||||||||| || |||||||||||||||

Sbjct 59115 AAAATTTTGTTGAGCTATATATATAATCCAATAAAAAAAGGGGACTGAATTTTGAAATAT 59056

Query 1029 ATTAAACTTTTTT----------TATAATAAAATAAATATAATTATTTTTAAATAATATA 1078

|||||||| |||| |||||||||||||||| ||||||||||||||| |

Sbjct 59055 ATTAAACTCTTTTTATAAAAAAATATAATAAAATAAATACAATTATTTTTAAATA----A 59000

Query 1079 TATTATATATATATATTATATATTATATATTTTTTTTTATCTTTACACAG--AATTTTTT 1136

|||||||| |||||||| ||||||| |||||||||||||||||||| || |||

Sbjct 58999 TATTATAT-TATATATT-----TTATATA-TTTTTTTTATCTTTACACAGAAAAAAATTT 58947

Query 1137 TTATAGAGTCCAATATATATAATTAGTTATAT--------ATATACACCACCATAAATAA 1188

|||||||||||||||||||||||||||||||| |||||||||| |||||||||

Sbjct 58946 TTATAGAGTCCAATATATATAATTAGTTATATATATAGAGATATACACCATCATAAATAA 58887

Query 1189 TTATAAGGAAAATCAGTTATTTAAATTTTAACTG--AAGAAATTAAATAAGTATATAAAA 1246

|||||||||||||||||||||||||||||||||| || ||||||||||||||||| ||

Sbjct 58886 TTATAAGGAAAATCAGTTATTTAAATTTTAACTGAAAAAAAATTAAATAAGTATAT-AAG 58828

Query 1247 AAAGAAAAGAACAAAAAATAAAACGACAACAACCTTAAAAATTTTTTATTATTACATATT 1306

||||||| |||||||||||||||| ||| |||||||||| || ||||||||||||||||

Sbjct 58827 AAAGAAACGAACAAAAAATAAAACCACAGCAACCTTAAATTTTGTTTATTATTACATATT 58768

Query 1307 TATACATAAAAAAAATAAAATACCTAGACAAAAAATAATAAATATTATAGATCGATATT- 1365

||||||||||| |||||||||||||||||||||||||||||||| ||||||||||||||

Sbjct 58767 TATACATAAAAGAAATAAAATACCTAGACAAAAAATAATAAATAATATAGATCGATATTA 58708

Query 1366 GACTACATATATACCTTTCAGCACATAATTACATCTGAATAAAATG 1411

| || |||||||||||||||| ||||||||| |||||||||||||

Sbjct 58707 GCATAGATATATACCTTTCAGCGCATAATTACGTCTGAATAAAATG 58662

**Promoter:** *pylisp2*

**Gene ID:** PY17X_1004400 (“Query”), PBANKA_1003000, PCHAS_1003900, (all genes are syntenic)

> [PbANKA_10_v3](https://plasmodb.org/plasmo/app/record/genomic-sequence/PbANKA_10_v3) | organism=Plasmodium_berghei_ANKA | version=2017-01-09

| length=1640193 | SO=chromosome

Length=1640193

Score = 2664 bits (2954), Expect = 0.0

Identities = 1726/1886 (92%), Gaps = 62/1886 (3%)

[**Link to Genome Browser**](https://plasmodb.org/plasmo/app/jbrowse?data=/plasmo/service/jbrowse/bySequenceId/PbANKA_10_v3/&loc=PbANKA_10_v3:190308-192177&tracks=gene), Strand = Plus/Minus

Query 2 TTAATAACCCATCAACATTTTGTTGAATTGTTTTGGATAAAAATGATTGTCATTGTCCTT 61

||||||| ||||||||||||| ||||||| ||||| ||||||||||||||| ||||||||

Sbjct 192177 TTAATAAACCATCAACATTTTTTTGAATTATTTTGAATAAAAATGATTGTCTTTGTCCTT 192118

Query 62 ATGTGCATATAACATCGTTTGATTTAATATATAAATTTAATATAATTTAAACAAACACAT 121

||| ||||||||||||||||||||||||||||||||||||||||||||||||||| ||||

Sbjct 192117 ATGGGCATATAACATCGTTTGATTTAATATATAAATTTAATATAATTTAAACAAAAACAT 192058

Query 122 ATATACATATAGGTGCATGTCTTATAGTTATTCTATAATTTGTAATTACATAGTCATAAT 181

|||||||||||||||||| |||||||||||||||||| | ||||||||||| |||||||

Sbjct 192057 ATATACATATAGGTGCATACCTTATAGTTATTCTATAAATCGTAATTACATAATCATAAT 191998

Query 182 TGTTTATTCTTGAAATATTGGTATTTTTTACATTAAAAAAAATATTATGAAAAAAAGTAT 241

||||||||||||||||| ||||||||||||||||||||||| ||||||||||||||||||

Sbjct 191997 TGTTTATTCTTGAAATACTGGTATTTTTTACATTAAAAAAA-TATTATGAAAAAAAGTAT 191939

Query 242 GTGTATATATAT----TGTGTAATTTTCCTCAATTTTGGGGAACATGTAAAAACTCTTGG 297

|||||||||||| |||||| |||||||| |||||||||||||||||||| ||||| |

Sbjct 191938 GTGTATATATATATATTGTGTACTTTTCCTCTATTTTGGGGAACATGTAAAA-CTCTTAG 191880

Query 298 AATTGTATTTTATAAAAATATTATTTTACATAATTTATACTATTGTATATTATTTTTTTA 357

|||||||||||||||||||||||||||| |||||||||||||||||||||||||||||||

Sbjct 191879 AATTGTATTTTATAAAAATATTATTTTATATAATTTATACTATTGTATATTATTTTTTTA 191820

Query 358 TTTTTTAAGTATTTTCGAAATTTTCACATTATTTTATTATGATATGTATATAAAGGTATG 417

||||||||||||||| |||| ||||| ||||||| |||||||||| ||||||||||||||

Sbjct 191819 TTTTTTAAGTATTTTTGAAACTTTCATATTATTTCATTATGATATATATATAAAGGTATG 191760

Query 418 TATATATAAATTGTTAAATAGTTCTCACACAAACGATGTTATCAATTTATTAAAGTATAT 477

||||||||||| ||||||| ||| ||||| |||||||| |||||||||||||||| ||||

Sbjct 191759 TATATATAAATGGTTAAATCGTTTTCACATAAACGATGCTATCAATTTATTAAAGCATAT 191700

Query 478 AATAATAAAAAAAAAAAAAAAGTATGATCAGAATAAAACATTCTTATAAATGTTCTTAAA 537

||||||| ||||||||| ||||||||| ||||||||| | |||||||||||||||||

Sbjct 191699 AATAATATAAAAAAAAA----GTATGATCAAAATAAAACAATATTATAAATGTTCTTAAA 191644

Query 538 CATAAAAAAATGGAATATAAACATTAAAAATAT-CACATCTTTTAAGTGTTCATTAGCAT 596

||| ||||||||||||||| ||||||||||| | ||||||||||||||||||||||||||

Sbjct 191643 CATCAAAAAATGGAATATATACATTAAAAATGTGCACATCTTTTAAGTGTTCATTAGCAT 191584

Query 597 ATACCATTTTTCGTATGATCTTTAAATCTAGAGGTTCTCTTACACATGTGAATGTATATG 656

||| |||||||||||||||||||||||||||| || ||| ||||||||||||||||||||

Sbjct 191583 ATATCATTTTTCGTATGATCTTTAAATCTAGAAGTCCTCCTACACATGTGAATGTATATG 191524

Query 657 TATATAATTTTTTAACGATGTAACAGTGTTGTATATATTATT----------------TA 700

|||||||||||||||||||||||||||||||||||||||||| ||

Sbjct 191523 TATATAATTTTTTAACGATGTAACAGTGTTGTATATATTATTGAAGATATACATTATTTA 191464

Query 701 TATTAAAAAATAATGTAAACAAAATTATAGGAATAAACTAATTTTGAATCATTTATTTGC 760

| ||||||||| ||||||||||||||||||||||||||||||||||||||||||||||||

Sbjct 191463 TGTTAAAAAATCATGTAAACAAAATTATAGGAATAAACTAATTTTGAATCATTTATTTGC 191404

Query 761 TTAAAAATTAACCACATTATATTTAATAAATATAAAATAAAAATATGCCACATCCATTCT 820

||||||||||||||||||||||||||||||||||||||||||||||||||||||||||||

Sbjct 191403 TTAAAAATTAACCACATTATATTTAATAAATATAAAATAAAAATATGCCACATCCATTCT 191344

Query 821 ATACTATTTATTGGTACATCGACATTCACCACAAAATATTTCGTAGAGTTGCATTATCAT 880

||||||||||||||||||||||||||| ||| |||||||||| ||||||||||||||| |

Sbjct 191343 ATACTATTTATTGGTACATCGACATTCGCCATAAAATATTTCATAGAGTTGCATTATCGT 191284

Query 881 CAAAAGGGTATAAAATTATAAAATATA---ACATAAAATATAACATAATGGTGAAACTAA 937

|||||| |||||||||||||||||| | ||||||||| | ||||||||||||||||

Sbjct 191283 CAAAAGTGTATAAAATTATAAAATAGAGCAACATAAAATGGATAATAATGGTGAAACTAA 191224

Query 938 AAATAACGAAATTCAAGTTGGTTGAATAAAATAAAAACACAACATAAAAGATCAAGGTAT 997

|||||| ||||| ||||||| | |||||||||||||||||||| |||||||||||

Sbjct 191223 AAATAATAAAATTAAAGTTGGGT-----AAATAAAAACACAACATAAAGGATCAAGGTAT 191169

Query 998 TATTTTTTTTTAAATATTCTTTATGAAAAACGATTAGAACAATTTAAAATAATGTATATT 1057

||||||||| |||| || |||||||||||| |||||||||||||||||||||| |||||

Sbjct 191168 TATTTTTTT--AAATTTTTTTTATGAAAAACTATTAGAACAATTTAAAATAATGCATATT 191111

Query 1058 ATGCTT-----GTACAATATAGTTAAAATAAAGCGAATAACGATACGATTATTCAATATG 1112

|||||| |||||||||| |||||||||| ||||||||||| ||||||||||||||

Sbjct 191110 ATGCTTTATTTGTACAATATAATTAAAATAAAACGAATAACGATGTGATTATTCAATATG 191051

Query 1113 CAGATTAATAATATATATGCATGTAAATGTGTATATATTTCTTCATTGTCAAATTGCTTA 1172

||||||||||||||||| ||||||||||| |||||| ||||||||||||||||| ||||

Sbjct 191050 CAGATTAATAATATATACACATGTAAATGTATATATACTTCTTCATTGTCAAATTCCTTA 190991

Query 1173 GGCATATGTAGCATATATGATTTTTGACGATTCTTTAAAAATATACAACTGTAATTCCTA 1232

||||||||||||||||||| ||||||||| ||||||||||||||||||||||||| ||||

Sbjct 190990 GGCATATGTAGCATATATGGTTTTTGACGGTTCTTTAAAAATATACAACTGTAATCCCTA 190931

Query 1233 ATAAATTAATGCAGTTGTAATCGAAAAAAAGGCATGTGCTCCCGTATCGATTTAACCATT 1292

|||||| |||||||||| ||||||| ||| ||| |||| |||||||||||||||||||

Sbjct 190930 ATAAATGAATGCAGTTGAAATCGAACAAATC-CATATGCTTCCGTATCGATTTAACCATT 190872

Query 1293 TAGGTATTGATCTATGATCGATCTACATGTGAAGAAAATGTGCAT-TTTTAAAAACAGTT 1351

|||||| |||||||||||||||||||||||||||||||| |||| |||||||||| ||

Sbjct 190871 TAGGTAGTGATCTATGATCGATCTACATGTGAAGAAAATTGGCATCTTTTAAAAACTCTT 190812

Query 1352 TCATAATGGTTTATGAATTTTTTAGATCATATATTGTTTAAACAAAAAATAAAACAAAT- 1410

|||||||||||||||| || ||||||||||||||| |||||||||||||||||| ||||

Sbjct 190811 TCATAATGGTTTATGAGTTCTTTAGATCATATATTATTTAAACAAAAAATAAAATAAATC 190752

Query 1411 ACCTATTGTTTGATATTCGATAAAGTGTGCAAACAAATTTGCATGTATGTATTCTATTAT 1470

||||||| ||| ||||||||||||| ||||||||||||||||||||||||||||||||||

Sbjct 190751 ACCTATTTTTTTATATTCGATAAAGCGTGCAAACAAATTTGCATGTATGTATTCTATTAT 190692

Query 1471 CGTTGGTTTTTTTTACACCACCTTTTTTTTTTATTTATAATGTTTTTCACCACAAAGCAA 1530

|||||||||||||||||||||||||||||| ||||||||||||||||||||||||||||

Sbjct 190691 CGTTGGTTTTTTTTACACCACCTTTTTTTT--ATTTATAATGTTTTTCACCACAAAGCAA 190634

Query 1531 CTATATATATAAAACACACTAAAAATATATAATTAAATTAAAAAATA-TAATAAAGAAAA 1589

||||||||| ||||||||||||||||||||||||||||||||||||| ||||||||||||

Sbjct 190633 CTATATATAAAAAACACACTAAAAATATATAATTAAATTAAAAAATAATAATAAAGAAAA 190574

Query 1590 TAAAAA--------------GCATATGTATTATACATACATGTATATTCTCCTGAATATG 1635

|||||| ||||||||||||||||| ||||||||||| |||| |||||

Sbjct 190573 TAAAAAATAAAAAATAAAAAGCATATGTATTATACATGCATGTATATTCCCCTGCATATG 190514

Query 1636 TATGTTTTCAGTTCAGATTAAGAACAAAAAAATACAGATAATTAAGCATTTATTTGTTAC 1695

||||||||||||||||||||||||||||||||||||||||||||||||||||||||||||

Sbjct 190513 TATGTTTTCAGTTCAGATTAAGAACAAAAAAATACAGATAATTAAGCATTTATTTGTTAC 190454

Query 1696 GTTTATACATGTCAATACCAAAAATATATATGTGCCTACACATGTTACATATAAGTAGAT 1755

||||||||||||||||||||||||||||||||||||||||||||||||||||||||||||

Sbjct 190453 GTTTATACATGTCAATACCAAAAATATATATGTGCCTACACATGTTACATATAAGTAGAT 190394

Query 1756 ACATATAAATATATCGGAGTAATATTTTTCTTCTTTAATTTTTCACTTTTATTATAATCA 1815

|||||||||||||||||||||||||| ||||||||| ||||||||||| |||||||||||

Sbjct 190393 ACATATAAATATATCGGAGTAATATTCTTCTTCTTTCATTTTTCACTTCTATTATAATCA 190334

Query 1816 TTTTACTTTTTTACACATAAAAAATG 1841

||||||||||||||||||||||||||

Sbjct 190333 TTTTACTTTTTTACACATAAAAAATG 190308

> [PCHAS_10_v3](https://plasmodb.org/plasmo/app/record/genomic-sequence/PCHAS_10_v3) | organism=Plasmodium_chabaudi_chabaudi | version=2015-06-18

| length=1632254 | SO=chromosome

Length=1632254

Score = 2064 bits (2288), Expect = 0.0

Identities = 1591/1882 (85%), Gaps = 109/1882 (6%)

[**Link to Genome Browser**](https://plasmodb.org/plasmo/app/jbrowse?data=/plasmo/service/jbrowse/bySequenceId/PCHAS_10_v3/&loc=PCHAS_10_v3:183975-185789&tracks=gene), Strand = Plus/Minus

Query 2 TTAATAACCCATCAACATTTTGTTGAATTGTTTTGGATAAAAATGATTGTCATTGTCCTT 61

||||||||| |||| |||| ||||||| ||||| ||||||||||||||| ||||||||

Sbjct 185789 TTAATAACC-ATCAGTATTTCTTTGAATTATTTTGAATAAAAATGATTGTCTTTGTCCTT 185731

Query 62 ATGTGCATATAACATCGTTTGATTTAATATATAAATTTAATATAATTTAAACAAACACAT 121

|||||||||| ||||||||||||| |||||||||||||| |||| |||| ||| |||||

Sbjct 185730 GTGTGCATATAGCATCGTTTGATTTTATATATAAATTTAACATAAATTAAGCAAGCACAT 185671

Query 122 ATATACATATAGGTGCATGTCTTATAGTTATTCTATAATTTGTAATTACATAGTCATAAT 181

|||||||||||||| || |||||||||||||||||||| ||||||||||||| | |||||

Sbjct 185670 ATATACATATAGGTACACGTCTTATAGTTATTCTATAAATTGTAATTACATAATTATAAT 185611

Query 182 TGTTTATTCTTGAAATATTGGTATTTTTTACATTAAAAAAAATATTATGAAAAAAAGTAT 241

|| ||||||||||||| ||||||||||||| || |||||||||||||||||||||||||

Sbjct 185610 TGCTTATTCTTGAAATGCTGGTATTTTTTACGTT-AAAAAAATATTATGAAAAAAAGTAT 185552

Query 242 GTGTATATATATTGTGTAATTTTCCTCAATTTTGGGGAACATGTAAAAACTCTTGGAATT 301

||||||||||||||||||||||| || ||||||||| |||||||||| || ||| |||

Sbjct 185551 GTGTATATATATTGTGTAATTTTATTCTATTTTGGGG-GCATGTAAAAATTCATGGCATT 185493

Query 302 GTATTTTATAAAAATATTATTTTACATAATTTATACTATTGTATATTATTTT-TTTATTT 360

|||||||| |||||||||||||||||| |||||||| |||||||

Sbjct 185492 ATATTTTAT----------------ATAATTTATACTATTGTACATTATTTTATTTATTT 185449

Query 361 TTTAAGTATTTTCGAAATTTTCACATTATTTTATTATGATATGTATATAAAGGTATGTAT 420

|||||| |||||| || ||| | |||||||||||||| |||| ||

Sbjct 185448 TTTAAGGATTTTCAAATTTTGTATTTTATTTTATTATGA--------------TATGCAT 185403

Query 421 ATATAAATTGTTAAATAGTTCTCACACAAACGATGTTATCAATTTATTAAAGTATATAAT 480

|||||||| ||||||||||| ||||| ||| ||||||||||||||||||| || ||

Sbjct 185402 ATATAAATGGTTAAATAGTTTTCACAAAAATGATGTTATCAATTTATTAAGCCATGCAAC 185343

Query 481 AATAAAAAAAAAAAAAAAGTATGATCAGAATAAAACATTCTTATAAATGTTCTTAAACAT 540

|||| ||||| ||| ||| |||||||| |||||||| | |||||||||||

Sbjct 185342 AATATAAAAA----------ATGTTCAAAATAAAACGATCTTATAATTATTCTTAAACAT 185293

Query 541 AAAAAAATGGAATATAAACATTAAAAATAT-CACATCTTTTAAGTGTTCATTAGCATATA 599

| ||||||||||||| ||||||||||| | || ||| |||||||| |||||||||||||

Sbjct 185292 CAGAAAATGGAATATACACATTAAAAATGTGCATATCCTTTAAGTGCTCATTAGCATATA 185233

Query 600 CCATTTTTCGTATGATCTTTAAATCTAGAGGTTCTCTTACACATGTGAATGTATATGTAT 659

||||||| |||||||||||||| || || |||| || ||| ||||| |||

Sbjct 185232 TCATTTTTTGTATGATCTTTAAACCTTGA------ACTACAAATTTGAGTGTATGCATAT 185179

Query 660 ATAATTTTTTAACGATGTAACAGTGTTGTATATATT----------------ATTTATAT 703

|||| ||||||| |||| |||||||||||||||||| |||||| |

Sbjct 185178 ATAAATTTTTAAAGATGCAACAGTGTTGTATATATTATTCGAAGATAATATGATTTATGT 185119

Query 704 TAAAAAATAATGTAAACAAAATTATAGGAATAAACTAATTTTGAATCATTTATTTGCTTA 763

|||||||||||||||||||| ||||||||||||||||||||||||||||||||||||||

Sbjct 185118 AAAAAAATAATGTAAACAAAACTATAGGAATAAACTAATTTTGAATCATTTATTTGCTTA 185059

Query 764 AAAATTAACCACATTATATTTAATAAATATAAAATAAAAATAT-GCCACATCCATTCTAT 822

|||||||||||||||||| |||||||||||||||||||||||| ||||||||||||||||

Sbjct 185058 AAAATTAACCACATTATACTTAATAAATATAAAATAAAAATATAGCCACATCCATTCTAT 184999

Query 823 ACTATTTATTGGTACATCGACATTCACCACAAAATATTTCGTAGAGTTGCATTATCATCA 882

||||||||||| | ||||||||||| |||||||||||||| |||||||| |||| | |||

Sbjct 184998 ACTATTTATTGCTGCATCGACATTCGCCACAAAATATTTCATAGAGTTGTATTA-CGTCA 184940

Query 883 AAAGGGTATAAAATTATAAAAT---ATAACATAAAATATAACATAATGGTGAAACTAAAA 939

|||| |||||||| |||||||| |||||||||||| | ||| ||||||||||||||

Sbjct 184939 AAAGTGTATAAAAGTATAAAATAGAATAACATAAAATGGATAATAGTGGTGAAACTAAAA 184880

Query 940 ATAACGAAATTCAAGTTGGTTGAATAAAATAAAAACACAACATAAAAGATCAAGGTATTA 999

||||| ||||| ||| ||| | |||||||||||||||||| ||||| ||||| || |

Sbjct 184879 ATAACAAAATTAAAGGTGGCTAAATAAAATAAAAACACAATATAAAGGATCATGG---CA 184823

Query 1000 TTTTTTTTTAAATATTCTTTATGAAAAACGATTAGAACAATTTAAAATAATGTATATTAT 1059

||||||||||||||||||||||||||||||||||||||| || ||||| ||| | |||||

Sbjct 184822 TTTTTTTTTAAATATTCTTTATGAAAAACGATTAGAACATTTAAAAATGATGCACATTAT 184763

Query 1060 GC-----TTGTACAATATAGTTAAAATAAAGCGAATAACGATACGATTATTCAATATGCA 1114

| || ||||||||||||||||| || ||||||| | |||||||||||||||| |

Sbjct 184762 TCTTTAATTATACAATATAGTTAAAATGAAACGAATAAAAACACGATTATTCAATATGTA 184703

Query 1115 GATTAATAATATATATGCATGTAAATGTGTATATATTTCTTCATTGTCAAATTGCTTAGG 1174

|||||||||||||||||||||||| |||||||||| |||||||| |||||||||||||

Sbjct 184702 GATTAATAATATATATGCATGTAAGTGTGTATATA---CTTCATTGCCAAATTGCTTAGG 184646

Query 1175 CATATGTAGCATATATGATTTTTGACGATTCTTTAAAAATATACAACTGTAATTCCTAAT 1234

||||||||||||||||| ||||| | | |||||||||| ||||||||||| | ||||||

Sbjct 184645 CATATGTAGCATATATGTTTTTTAATGGTTCTTTAAAATCATACAACTGTAGTCCCTAAT 184586

Query 1235 AAATTAATGCAGTTGTAATCGAAAAAAAGGCATGTGCTCCCGTATCGATTTAACCATTTA 1294

||||||||||||||| ||||| ||||| ||||| || | |||||||||||| |||||||

Sbjct 184585 AAATTAATGCAGTTGCAATCG-AAAAAGGGCATATGATATCGTATCGATTTAGCCATTTA 184527

Query 1295 GGTATT-----------GATCTATGATCGATCTACATGTGAAGAAAAT--GTGCATTTTT 1341

|||||| |||||||||||||||||||||||||||| | || || |||

Sbjct 184526 GGTATTGATCTATTATCGATCTATGATCGATCTACATGTGAAGAATGTTGGTACA-ATTT 184468

Query 1342 AAAAACAGTTTCATAATGGTTTATGAATTTTTTAGATCATATATTGTTTAAACAAAAAAT 1401

||||||||||| ||||||||||||||||| |||||||||||||| | ||||||| |||||

Sbjct 184467 AAAAACAGTTTTATAATGGTTTATGAATTATTTAGATCATATATCGCTTAAACACAAAAT 184408

Query 1402 AAAACAAAT-ACCTATTGTTTGATATTCGATAAAGTGTGCAAACAAATTTGCATGTATGT 1460

||||||||| | |||| |||||||||||||||| |||||||| | ||||||||||||||

Sbjct 184407 AAAACAAATCATTTATTTTTTGATATTCGATAAAATGTGCAAAAATATTTGCATGTATGT 184348

Query 1461 ATTCTATTATCGTTGGTTTTTTTTACACCACCTTTTTTTTTTATTTATAATGTTTTTCAC 1520

|||||||||||||||||||||||| |||||||||||||||| ||||||||||||||||||

Sbjct 184347 ATTCTATTATCGTTGGTTTTTTTTGCACCACCTTTTTTTTTAATTTATAATGTTTTTCAC 184288

Query 1521 CACAAAGCAACTATATATATAAAACACACTAAAAATATATAATTAAATTAAAAAAT-ATA 1579

||||||| |||||| |||| |||||||||||||||||||||||||||||||||||| |||

Sbjct 184287 CACAAAGAAACTATGTATAAAAAACACACTAAAAATATATAATTAAATTAAAAAATAATA 184228

Query 1580 ATAAAGAAAATAAAAAGCATATGTATTATACATACATGTATATTCTCCTGAATATGTATG 1639

|||||||||||||||| ||| | || | | ||| | |||| | |||||||||

Sbjct 184227 ATAAAGAAAATAAAAA--ATAAAAAATA-AAAAGCATATGTATTAT-----ATATGTATG 184176

Query 1640 TTTTCAGTTCAGATTAAGAACAAAAAAATACAGATAATTAAGCATTTATTTGTTACGTTT 1699

|||||||||||||||||||||||||||||||||||| ||||||||||| ||||||||||

Sbjct 184175 TTTTCAGTTCAGATTAAGAACAAAAAAATACAGATAGTTAAGCATTTAGTTGTTACGTTA 184116

Query 1700 ATACATGTCAATACCAAAAATATATATGTGCCTACACATGTTACATATAAGTAGATACAT 1759

|| |||| |||||||||||||||||||||||||||||||| |||||||||||||||||||

Sbjct 184115 ATTCATGCCAATACCAAAAATATATATGTGCCTACACATGATACATATAAGTAGATACAT 184056

Query 1760 ATAAATATATCGGAGTAATATTTTTCTTCTTTAATTTTTCACTTTTATTATAATCATTTT 1819

|||||||||||| | || |||||| |||||| ||||||| | || ||||||| ||| |||

Sbjct 184055 ATAAATATATCGAAATAGTATTTTACTTCTTGAATTTTTTATTTCTATTATAGTCA-TTT 183997

Query 1820 ACTTTTTTACACATAAAAAATG 1841

| || |||||||||||||||||

Sbjct 183996 ATTTATTTACACATAAAAAATG 183975

**Promoter:** *pybip*

**Gene ID:** PY17X_0822200 (“Query”), PBANKA_0818900, PCHAS_0819200 (all genes are syntenic)

> [PbANKA_08_v3](https://plasmodb.org/plasmo/app/record/genomic-sequence/PbANKA_08_v3) | organism=Plasmodium_berghei_ANKA | version=2017-01-09

| length=1420537 | SO=chromosome

Length=1420537

Score = 744 bits (824), Expect = 0.0

Identities = 479/516 (93%), Gaps = 23/516 (4%)

[**Link to Genome Browser**](https://plasmodb.org/plasmo/app/jbrowse?data=/plasmo/service/jbrowse/bySequenceId/PbANKA_08_v3/&loc=PbANKA_08_v3:754914-755410&tracks=gene), Strand = Plus/Plus

Query 1 GCATATTATATCACATATTTTATGAATGTGCATAATATTTATTGCTTGTTCATACAAATA 60

|||||||||||||||||||| |||||||||||||||||||||||||||||||||||||||

Sbjct 754914 GCATATTATATCACATATTT-ATGAATGTGCATAATATTTATTGCTTGTTCATACAAATA 754972

Query 61 TTTTAATATGTGTTCAAAAGTAAGGGATTAGTGAGAGGTAAAAAAAAATACAATTATTTT 120

|||||||||||||||||||||||||||||| ||||||| |||||||||||| ||||||||

Sbjct 754973 TTTTAATATGTGTTCAAAAGTAAGGGATTAATGAGAGGAAAAAAAAAATACGATTATTTT 755032

Query 121 GATATTGCTTAAAATATAGATATTTTATATAACATTAAAAAAAATTAATAATAACCCAAA 180

|||||||||||||||||||||| |||||||||||||||||||||||||| | || ||

Sbjct 755033 GATATTGCTTAAAATATAGATA-TTTATATAACATTAAAAAAAATTAAT----AGCCCAA 755087

Query 181 AAAAAATAATATATATGCATAGAAGAATTCATACATTGTGCTTAAAAAGAAATATTTAAG 240

|||||||||||||||||||||||||||||||||||||||||||||||| |||||||||||

Sbjct 755088 AAAAAATAATATATATGCATAGAAGAATTCATACATTGTGCTTAAAAATAAATATTTAAG 755147

Query 241 TTTTTGTGAAGCTATTCTAAAAAAAACGGATAAATATATATTTT-TGTATATTATATATA 299

||||||| |||||||||||||||||||| ||||||||||||||| |||||||||||||||

Sbjct 755148 TTTTTGTAAAGCTATTCTAAAAAAAACGCATAAATATATATTTTATGTATATTATATATA 755207

Query 300 AAATATGTATATGTAAATATTAAAAAAAATATATATATTATATATTTTTTTTTGAAATTA 359

||||||||||||||||||||||||||||||||||| ||||||||| ||||||| ||||||

Sbjct 755208 AAATATGTATATGTAAATATTAAAAAAAATATATA-ATTATATATATTTTTTT-AAATTA 755265

Query 360 ATATTAAAGCTATTGTTAAAAAAAAAAAAAAAAAAAGGGAACTTTTATTGGAAATATACA 419

||||||||| ||||||| ||||||||||||||||||||||||||||||||

Sbjct 755266 ATATTAAAGTTATTGTT----------TAAAAAAAAGGGAACTTTTATTGGAAATATACA 755315

Query 420 TTTTGAATAATTTTTACCTTAGTTTTGAACAAGAATTAATTACATTATTATTTTTATTAA 479

||||||||| ||||||||||||||||||||||||| ||||||||||||||||| ||||||

Sbjct 755316 TTTTGAATATTTTTTACCTTAGTTTTGAACAAGAA-TAATTACATTATTATTTATATTAA 755374

Query 480 TTAATTTTGTATGCAT---AAAAATTAAAGCAAATG 512

|| ||||||||||||| |||||||||||||||||

Sbjct 755375 TTTATTTTGTATGCATAAAAAAAATTAAAGCAAATG 755410

> [PCHAS_08_v3](https://plasmodb.org/plasmo/app/record/genomic-sequence/PCHAS_08_v3) | organism=Plasmodium_chabaudi_chabaudi | version=2015-06-18

| length=1414978 | SO=chromosome

Length=1414978

Score = 603 bits (668), Expect = 6e-172

Identities = 459/529 (87%), Gaps = 40/529 (8%)

[**Link to Genome Browser**](https://plasmodb.org/plasmo/app/jbrowse?data=/plasmo/service/jbrowse/bySequenceId/PCHAS_08_v3/&loc=PCHAS_08_v3:783059-783564&tracks=gene), Strand = Plus/Plus

Query 1 GCATATTATATCACATATTTTATGAATGTGCATAATATTTATTGCTTGTTCATACAAATA 60

|||| |||||| | |||||| ||||||||||||||||||||||||||||||||||||| |

Sbjct 783059 GCATTTTATATAATATATTT-ATGAATGTGCATAATATTTATTGCTTGTTCATACAAAAA 783117

Query 61 TTTTAATATGTGTTCAAAAGT-AAGGGATTAGTGAGAGGTAAAAAAAAATACAATTATTT 119

| ||||||||||||||||||| ||||||||||||||||| ||||||||| ||| |

Sbjct 783118 TGTTAATATGTGTTCAAAAGTTAAGGGATTAGTGAGAGGAAAAAAAAAAGTG---TATAT 783174

Query 120 TGATATTGCTTAAAATATAGATATTTTATATAACATTAAAAAAAA-TTAATAATAACCCA 178

| ||||| |||||| |||||||||||||||||||||||||||||| ||||||||||||||

Sbjct 783175 TAATATTTCTTAAA-TATAGATATTTTATATAACATTAAAAAAAAATTAATAATAACCCA 783233

Query 179 AAAAAAAATAATAT-ATATGCATAGAAGAATTC-ATACATTGTGCTTAAAAAGAAATATT 236

|||||| |||||| ||||||||||||||||| || || |||||||||||||||||||

Sbjct 783234 AAAAAA--TAATATTATATGCATAGAAGAATTTTATGCAACGTGCTTAAAAAGAAATATT 783291

Query 237 TAAGTTTTTGTGA---AGCTATTCTAAAAAAAACGGATAAATATATATTTT-TGTATATT 292

||||||||||||| ||||||||||||||| | | ||||||||||||||| ||||| ||

Sbjct 783292 TAAGTTTTTGTGACGAAGCTATTCTAAAAAATAAGCATAAATATATATTTTATGTAT-TT 783350

Query 293 ATATATAAAATATGTATATGTAAATATTAAAAAAAATATATATAT------TATATATTT 346

|||||||||||| |||| ||||||| | |||||||||||||||| |||||||||

Sbjct 783351 GTATATAAAATATATATA-GTAAATAATTAAAAAAATATATATATATATGTTATATATTT 783409

Query 347 TTTTTTGAAATTAATATTAAAGCTATTGTTAAAAAAAAAAAAAAAAAAAGGGAACTTTTA 406

|||||| ||||||||||||||||||||||||||||| ||||| || ||

Sbjct 783410 TTTTTT-AAATTAATATTAAAGCTATTGTTAAAAAA-------------GGGAATTTATA 783455

Query 407 TTGGAAATATACATTTTGAATAATTTTTACCTTAGTTTTGAACAAGAATTAATTACATTA 466

|||||||||||| |||||||||||||| | |||||||||||||| ||||||||||||||

Sbjct 783456 TTGGAAATATACGTTTTGAATAATTTTCATTTTAGTTTTGAACAATAATTAATTACATTA 783515

Query 467 TTATTTTTATTAATTAATTTTGTATGCAT---AAAAATTAAAGCAAATG 512

||||||||||||||||||||||||||||| |||||||||||||||||

Sbjct 783516 TTATTTTTATTAATTAATTTTGTATGCATAAAAAAAATTAAAGCAAATG 783564
